# Supplementary material for: An Integrated Analytical Approach Reveals Trichome Acylsugar Metabolite Diversity in the Wild Tomato Solanum pennellii
Source: Metabolites. 2020 Oct 9;10(10):401. doi: 10.3390/metabo10100401 (PMC7599763; doi:10.3390/metabo10100401)
Supplement: Supplementary file 1 [file metabolites-10-00401-s001.pdf]

Table S1 Plant growth metadata

Species	*Solanum pennellii*
Genotypes	LA0716<br>LA1272<br>LA1340<br>LA1376<br>LA1523<br>LA1656<br>LA1674<br>LA1693<br>LA1773<br>LA1809<br>LA1941<br>LA1946<br>LA2560<br>LA2657<br>LA2719<br>LA2963
Organ	Leaf
Organ specification	Leaflets; leaflets were collected from youngest fully expanded leaves
Cell type	Extraction procedure selectively extracts metabolites from glandular trichomes
Biosource amount	Single leaflet per biological replicate; six biological replicates per genotype
Growth location	Michigan State University Growth Chamber Facility chamber 10
Seedling establishment	
Date of plant establishment	12 August 2019
Plant growth stage	Cotyledon stage
Growth substrate	Peat pots (Hummert, Earth City, MO)
Light	16 h/8 h light/dark; 190 µmol m^{-2} s^{-1} photosynthetic photon flux density (PPFD) (cool white fluorescent)
Humidity	75% (measured)
Temperature	21°C
Watering regime	Twice weekly; de-ionized water by bottom watering
Nutritional regime	Once weekly; half-strength Hoagland's solution by bottom watering [1]
Transplant	
Date of transplant	9 September 2019
Plant growth stage	1st pair of true leaves

Growth substrate	Peat-based propagation mix (SunGro, Agawam, MA) in 9-cm pots
Light	12 h/12 h light/dark; 600 µmol m^{-2} s^{-1} PPFD (high pressure sodium/metal halide)
Humidity	50% (chamber setpoint)
Temperature	28°C/12°C day/night
Watering regime	Once weekly; de-ionized water by bottom watering
Nutritional regime	Once weekly; half-strength Hoagland's solution by bottom watering [1]
Harvest date, time	9 December 2019; between 1400 and 1700
Plant growth stage	Mature flowering (16 weeks)

1. Hoagland, D.; Arnon, D. *The water-culture method for growing plants without soil*; Berkeley, Calif. : College of Agriculture, University of California, 1950.

Table S2 Untargeted metabolomics LC-MS metadata.

Facility supervisor	Prof. A. D. Jones
Analyst	Daniel B. Lybrand
LC system	Waters Acquity UPLC
Autosampler	Waters 2777C
Column	Waters BEH C18 UPLC (2.1 x 100 mm; 1.7 µm)
Injection volume	5 µL
Flow rate	0.4 mL/min
Mobile phases	
A	10 mM ammonium formate in water with 5 mL/L 85% formic acid (final pH 2.8)
B	10 mM ammonium formate in 90% acetonitrile with 5 mL/L 85% formic acid
Gradient profile	0% B at 0-1 min, 55% B at 1.01 min, 100% B at 16-18 min, 0% B at 18.01-20 min.
Column oven temperature	40°C
Autosampler temperature	10°C
Mass spectrometer	Waters G2-XS QToF
Software	MassLynx v4.2
Ionization source	Electrospray ionization
Data acquisition	Sensitivity mode, continuum
Polarity	Positive
Mass range	*m/z* 50-1500
Scan time	0.5 s
Capillary voltage	3.00 kV

Sampling cone voltage	35 V
Source offset	80 V
Source temperature	100°C
Desolvation temperature	350°C
Cone gas flow	50.0 L/h
Desolvation gas flow	600 L/h
Collision energy	
Function 1	6 eV
Function 2	15-40 eV
Lockmass reference	Leucine enkephalin (*m/z* 556.2766)
Data correction	Not applied

Table S3 Sugar core quantification LC-MS metadata.

Facility supervisor	Prof. A. D. Jones
Analyst	Daniel B. Lybrand
LC system	Waters Acquity UPLC
Autosampler	Waters 2777C
Column	Waters BEH Amide UPLC (2.1 x 100 mm; 1.7 µm)
Injection volume	5 µL
Flow rate	0.5 mL/min
Mobile phases	
A	10 mM ammonium bicarbonate in 50% acetonitrile (100 mM ammonium bicarbonate, pH 8.0 stock solution diluted with H_2O and acetonitrile)
B	10 mM ammonium bicarbonate in 90% acetonitrile (100 mM ammonium bicarbonate, pH 8.0 stock solution diluted with acetonitrile)
Gradient profile	100% B at 0 min, 0% B at 5 min, 100% B at 5.01-10 min.
Column oven temperature	40°C
Autosampler temperature	10°C
Mass spectrometer	Waters TQD
Software	MassLynx v4.2
Ionization source	Electrospray ionization
Data acquisition	Multiple Reaction Monitoring (MRM)
Polarity	Negative
Mass transitions	

Glucose	*m/z* 179 > 89
Dwell time	0.077 s
Cone voltage	16 V
Collision potential	10 V
$^{13}C_6$-glucose	*m/z* 185 > 92
Dwell time	0.077 s
Cone voltage	16 V
Collision potential	10 V
Sucrose	*m/z* 341 > 89
Dwell time	0.077 s
Cone voltage	40 V
Collision potential	22 V
$^{13}C_6$-sucrose	*m/z* 353 > 92
Dwell time	0.077 s
Cone voltage	40 V
Collision potential	22 V

Table S4 Oligonucleotide primers.

Primer name	Oligonucleotide sequence	Efficiency (%)
RT_ASFF_F	CTACGCAGGCAGATGTAGAAA	99
RT_ASFF_R	ATCACTAGAAGGCAAGTGTAAGG	
RT_EF-1a_F	TGCTGCTGTAACAAGATGGA	85
RT_EF-1a_R	AGGGGATTTTGTCAGGGTTG	
RT_actin_F	GGTCGTACCACTGGTATTGT	98
RT_actin_R	AAACGAAGAATGGCATGTGG	
RT_ubiquitin_F	TCGTAAGGAGTGCCCTAATGCTGA	101
RT_ubiquitin_R	CAATCGCCTCCAGCCTTGTTGTAA	
gDNA_EF-1a_F	GTTTGCTTTAATTCGTAGATGGAATTAATT	N/A
gDNA_EF-1a_R	CCA GTA GGG CCA AAG GTC ACA	

Table S5 NMR metadata.

Analysis description	
**Supervisor**	Dr. Daniel Holmes
**Operator**	Dr. Thilani Anthony
**Institution**	Michigan State University
**Data and time of data acquisition**	October 2019 - December 2019
**Sample description**	
**Field frequency lock**	Chloroform-d_1
**Additional solute**	None
**Solvent**	$CDCl_3$ (600 μL 99.96 atom % D, Sigma-Aldrich)
**Chemical shift standard**	$CDCl_3$
**Concentration standard**	None
**Instrument description**	
**Agilent DirectDrive2 500 MHz NMR**	
**Geographic location of the instrument**	42.7288, -84.4745
**Magnet**	499.70 MHz
**Probe**	OneNMR Probe with Protune accessory for hands-off tuning
**Autosampler**	7600AS 96 sample autosamplers
**Acquisition software**	VnmrJ 3.2A

Table S5 (cont'd)

Acquisition parameters	
**Agilent DirectDrive2 500 MHz NMR**	
**a) Acquisition parameters file reference**	^{1}H: VnmrJ/ Experiment Selector/ Common/ PROTON<br>^{13}C: VnmrJ/ Experiment Selector/ Common/ CARBON<br>HSQC: VnmrJ/ Experiment Selector/ Common/ (HC)HSQCAD<br>HMBC: VnmrJ/ Experiment Selector/ Common/ (HC)gHMBCAD<br>COSY: VnmrJ/ Experiment Selector/ Common/ (HH)gCOSY<br>J-resolved: VnmrJ/ Experiment Selector/ Liquid/ JSpectra/ HOMO2DJ
**b) Sample details**	Tube: Kontes NMR tube, 8 in<br>Temperature: 25 °C
**c) Instrument operation details**	Radiation frequency:<br>^{1}H: 499.90<br>^{13}C: 125.71<br>HSQC: 499.90, 125.71<br>HMBC: 499.90, 125.71<br>COSY: 499.90, 499.90<br>J-resolved: 499.90<br>Acquisition nucleus:<br>^{1}H: 90° = 7.9 μs, ^{13}C: 90° = 10.20 μs
**d) Number of data points acquired**	^{1}H: 16384<br>^{13}C: 32768<br>HSQC: 1202, 128<br>HMBC: 1202,200<br>COSY: 674, 200<br>J-resolved: 2810, 64
**e) Data acquisition details**	^{1}H: number of scans: 32<br>^{13}C: number of scans: 256<br>HSQC: t1 increments: 400; scan per t1 increment: 4<br>HMBC: t1 increments: 512; scan per t1 increment: 4<br>COSY: t1 increments: 512; scan per t1 increment: 4-16<br>J-resolved: t1 increments: 128; scan per t1 increment: 16

Table S5 (cont'd)

Spectral processing parameters	
**Agilent DirectDrive2 500 MHz NMR**	
**a) Software**	VnmrJ 3.2 A
**b) Process weighting**	^{1}H: LineBroaden<br>^{13}C: LineBroaden<br>HSQC: gaussian (F2); gaussian (F1)<br>HMBC: sqsinebell (F2); gaussian (F1)<br>COSY: sqsinebell (F2); sqsinebell (F1)<br>J-resolved: sinebell (F2); sinebell (F1)

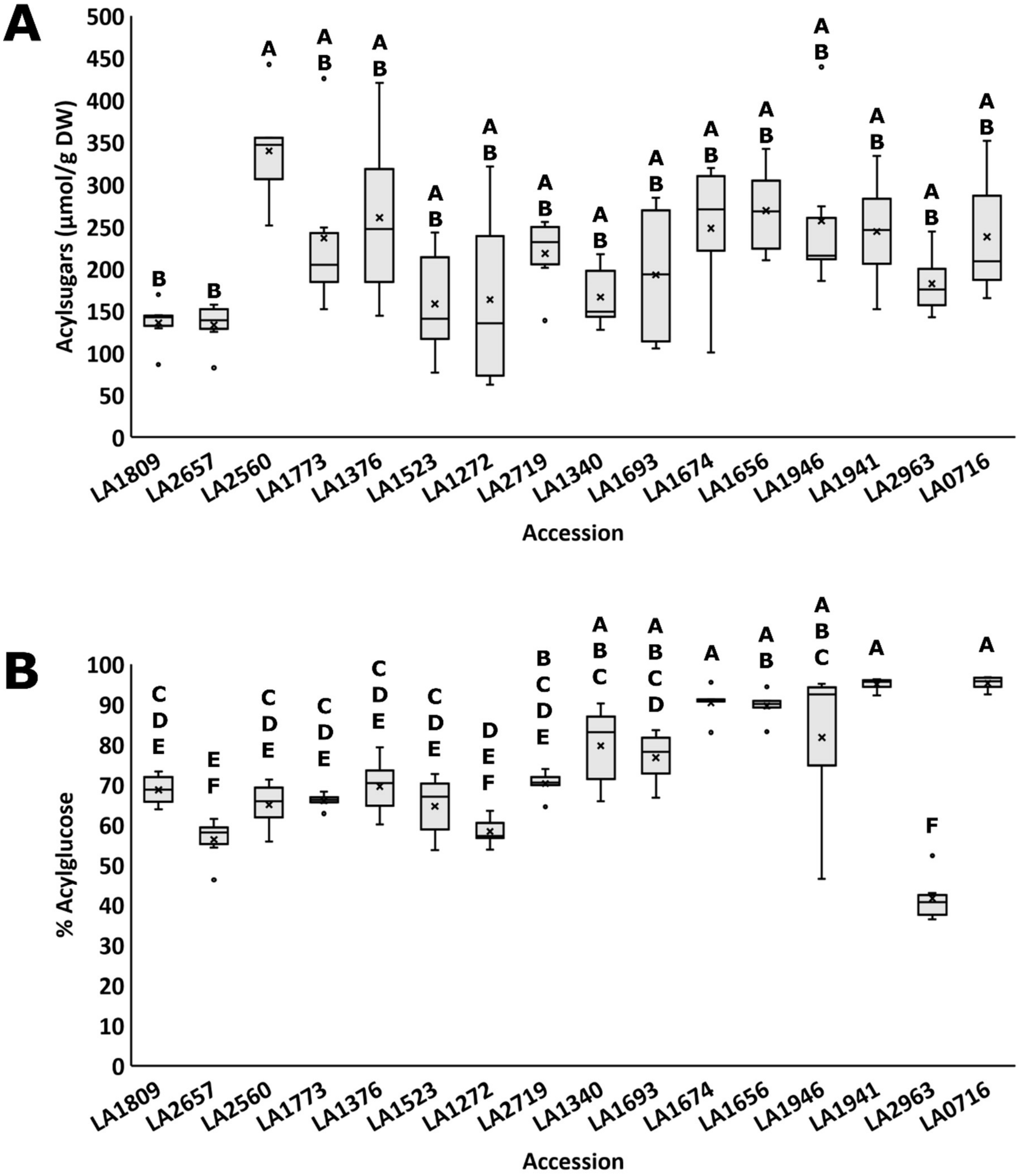

Figure S1 Quantification of acylsugars in 16 accessions of *S. pennellii*. Accessions are arranged left to right by latitude from north to south. (A) Total acylsugars. (B) Percent acylglucose accumulation. Results of ANOVA and Tukey's mean-separation test are indicated by letters; accessions that do not share at least one letter are significantly different from one another ($p < 0.001$, $n = 6$ for all accessions).

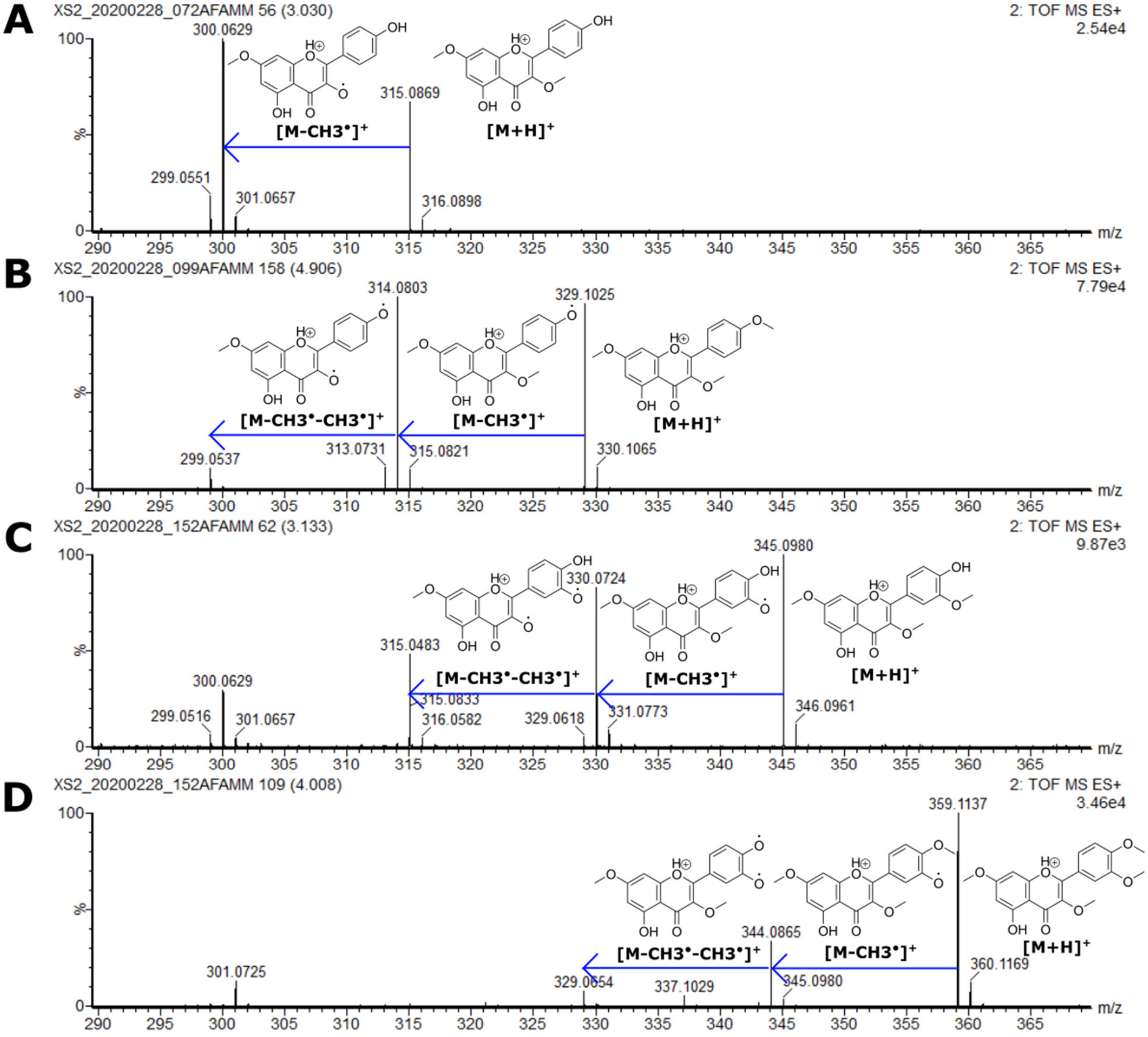

Figure S2 CID mass spectra of flavonoids extracted from *S. pennellii* analyzed by ES+ UHPLC-HR-MS. (A) Flavonoid A; (B) flavonoid B; (C) flavonoid C; (D) flavonoid D. See Table 4.3 for additional details.

Table S6 NMR chemical shifts for S3:12(4,4,4) Purified from *S. pennellii* LA0716.

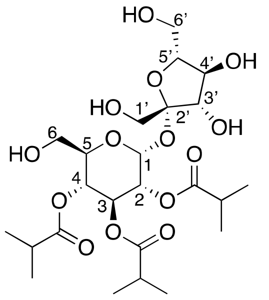

S3:12(4,4,4)

Purified from *S. pennellii* LA0716

Chemical Formula: $C_{24}H_{40}O_{14}$

HRMS: (ESI) *m/z* calculated for $C_{24}H_{40}O_{14}$ ($[M+NH_4]^+$): 570.2756

Experimental *m/z*: 570.2778

InChI Key: LHNYRVYQFVCMPK-NPCJRIMBSA-N

NMR (500 MHz, $CDCl_3$)

Sample mass: 2 mg

**Carbon #<br>(group)**	**^{1}H (ppm)**	**^{13}C (ppm)**<br>(from HSQC and HMBC)
**1** (CH)	5.76 (d, *J* = 4.0 Hz, 1H)	88.78
**2** (CH)	4.86 (dd, *J* = 10.3, 4.0 Hz, 1H)	70.74
- 1 (CO)	-	176.72
- 2 (CH)	2.46 (hept, *J* = 7.1 Hz, 1H)	33.88
- 3,4 (CH_3)	1.09 (d, *J* = 7.0 Hz, 6H)	18.87
**3** (CH)	5.55 (t, *J* = 9.9 Hz, 1H)	69.10
- 1 (CO)	-	176.56
- 2 (CH)	2.53 (hept, *J* = 7.0 Hz, 1H)	33.85
- 3,4 (CH_3)	1.13 (d, *J* = 7.0 Hz, 6H)	18.86
**4** (CH)	4.93 (t, *J* = 10.0 Hz, 1H)	68.44
- 1 (CO)	-	176.16
- 2 (CH)	2.53 (hept, *J* = 7.0 Hz, 1H)	33.85
- 3,4 (CH_3)	1.13 (d, *J* = 7.0 Hz, 6H)	18.86
**5** (CH)	4.23 (m, 1H)	71.86
**6** (CH_2)	3.60 (m, 2H)	61.50
**1′** (CH_2)	3.61 (m, 1H), 3.51 (d, *J* = 11.9 Hz, 1H)	64.41
**2′** (C)	-	104.44
**3′** (CH)	4.27 (m, 1H)	77.89
**4′** (CH)	4.27 (m, 1H)	73.10
**5′** (CH)	3.76 (m, 1H)	81.34
**6′** (CH_2)	3.88 (d, *J* = 13.0 Hz, 1H), 3.75 (m, 1H)<br>-	60.13

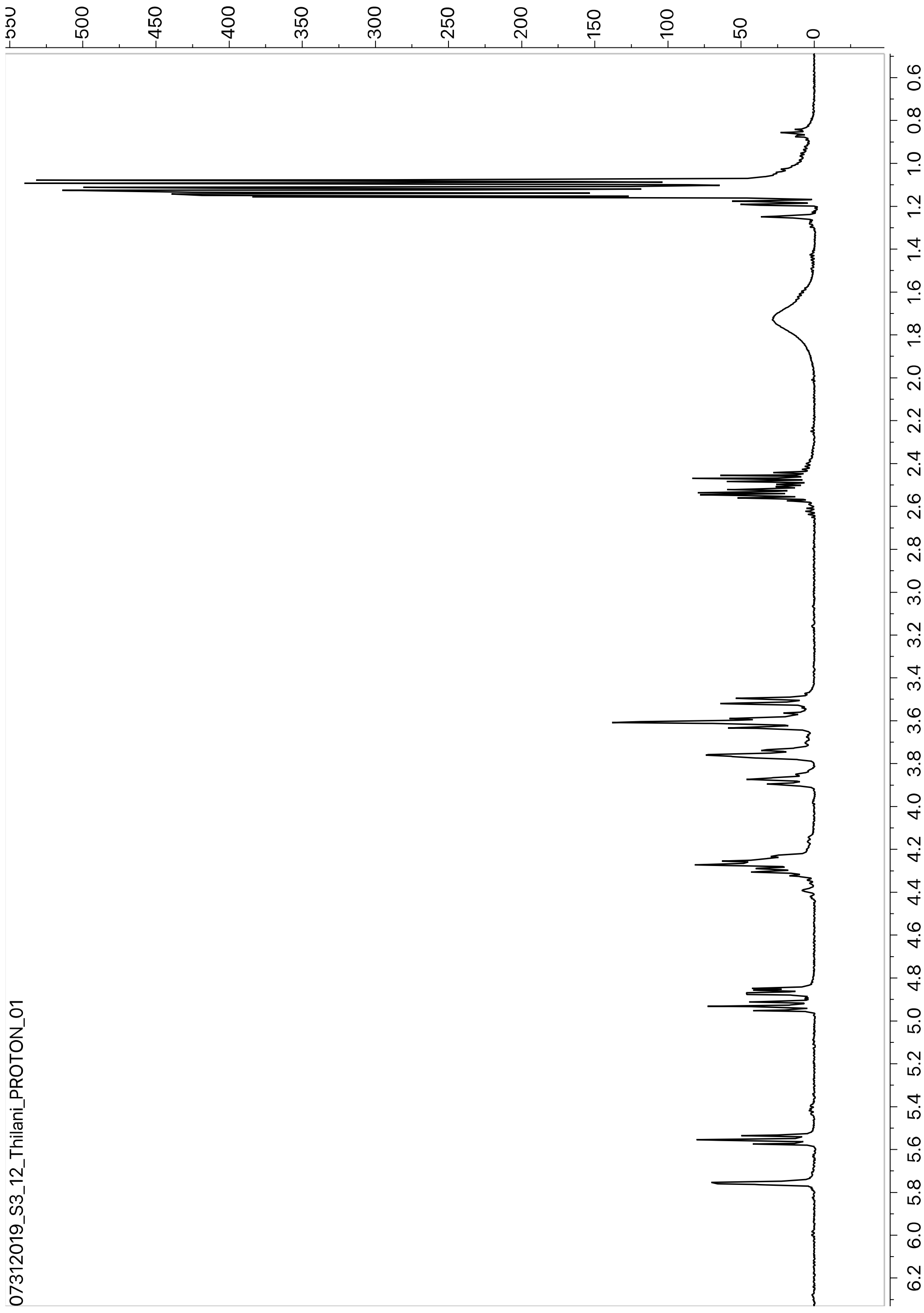

Figure S3 ^{1}H NMR spectrum for S3:12(4,4,4) purified from *S. pennellii* LA0716.

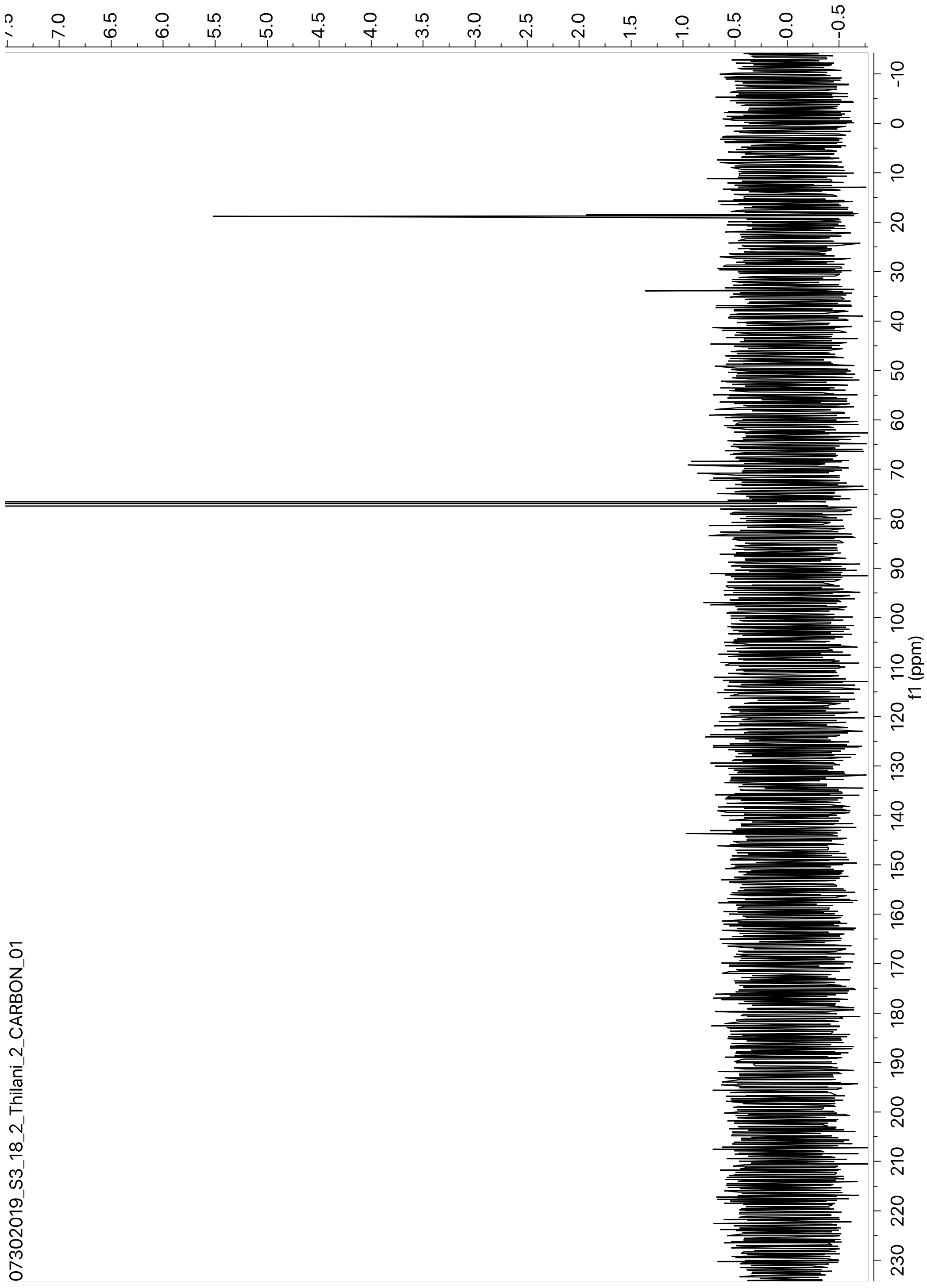

Figure S4 ^{13}C NMR spectrum for S3:12(4,4,4) purified from *S. pennellii* LA0716.

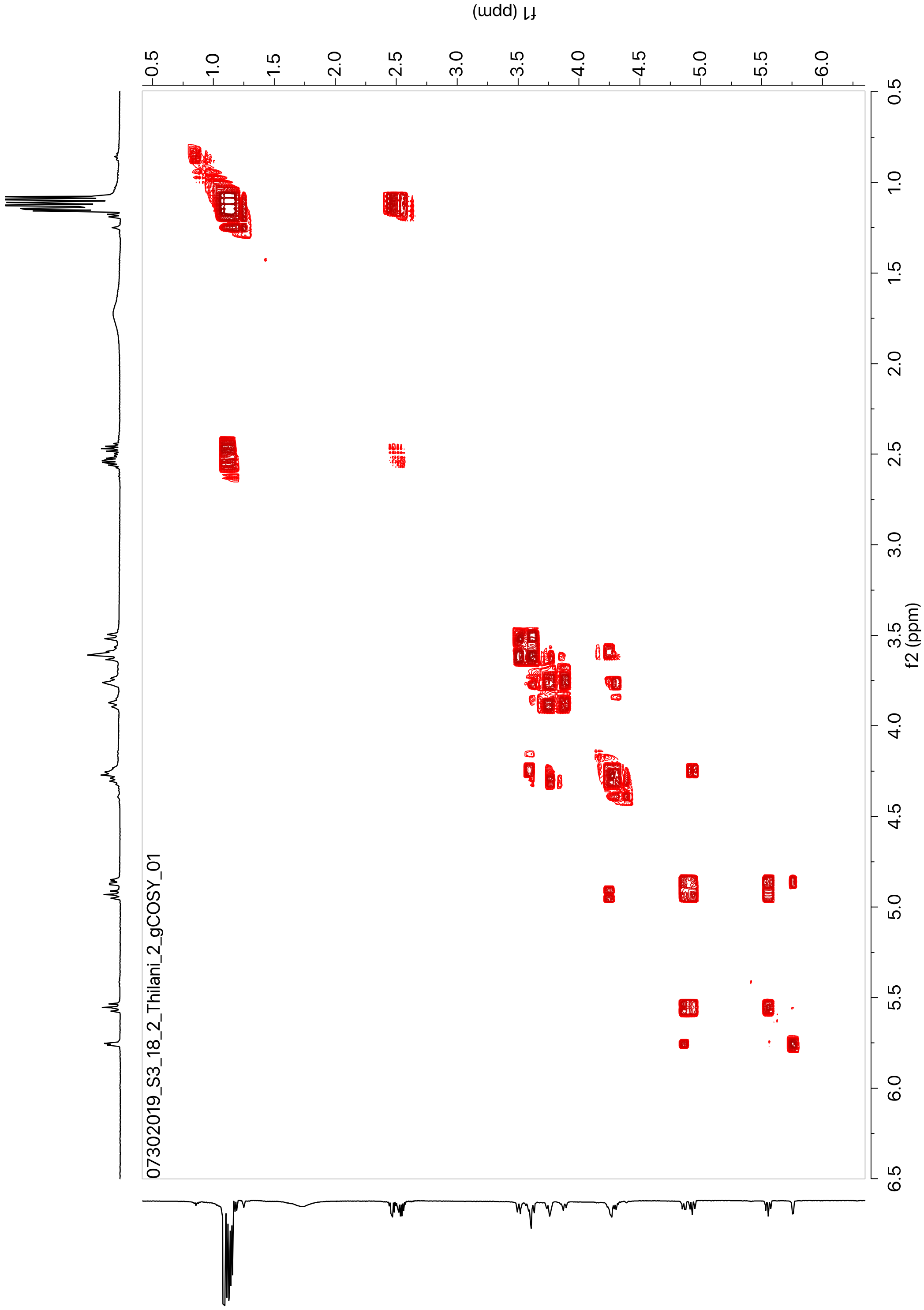

Figure S5 gCOSY NMR spectrum for S3:12(4,4,4) purified from *S. pennellii* LA0716.

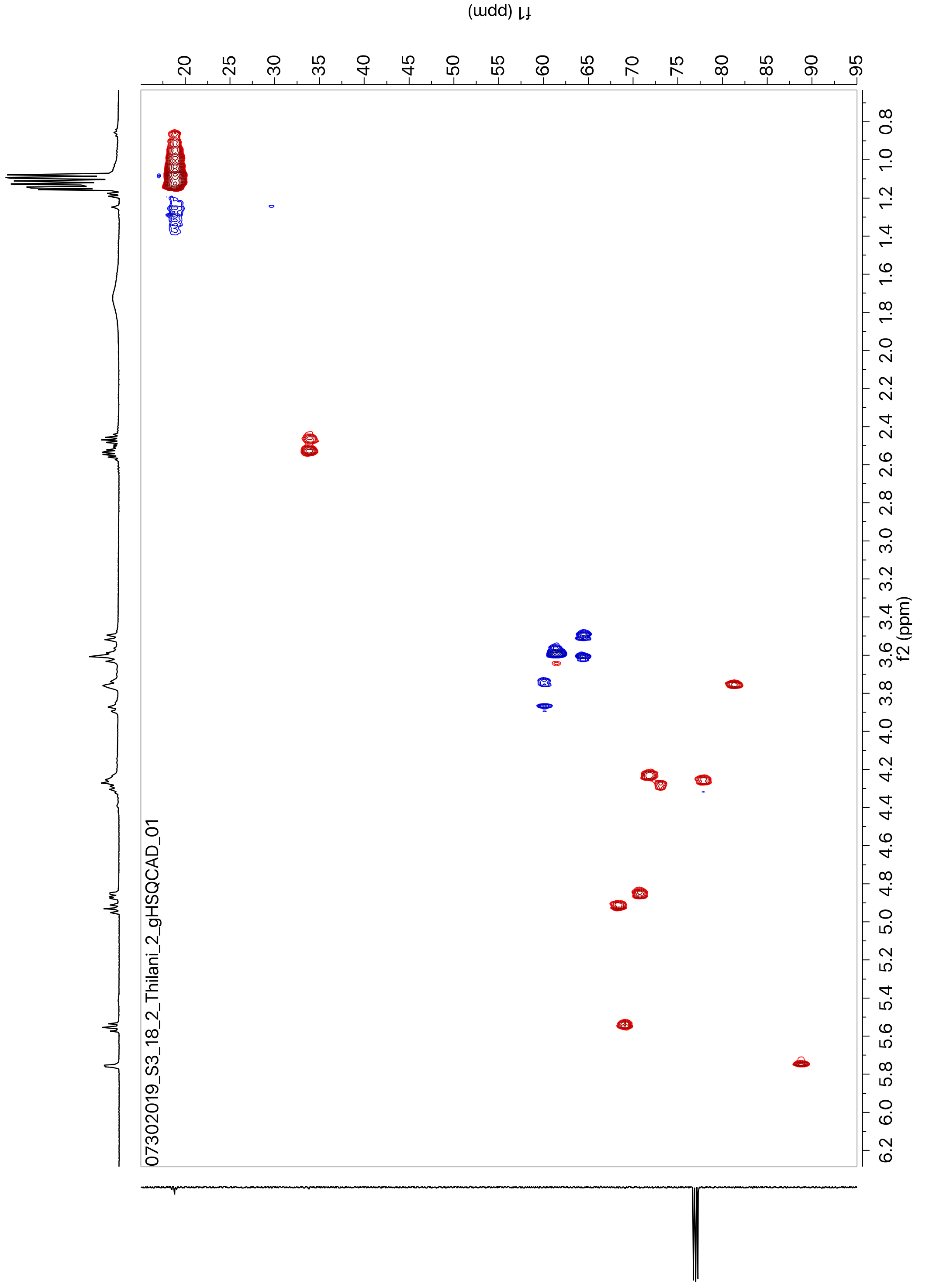

Figure S6 gHSQCAD NMR spectrum for S3:12(4,4,4) purified from *S. pennellii* LA0716.

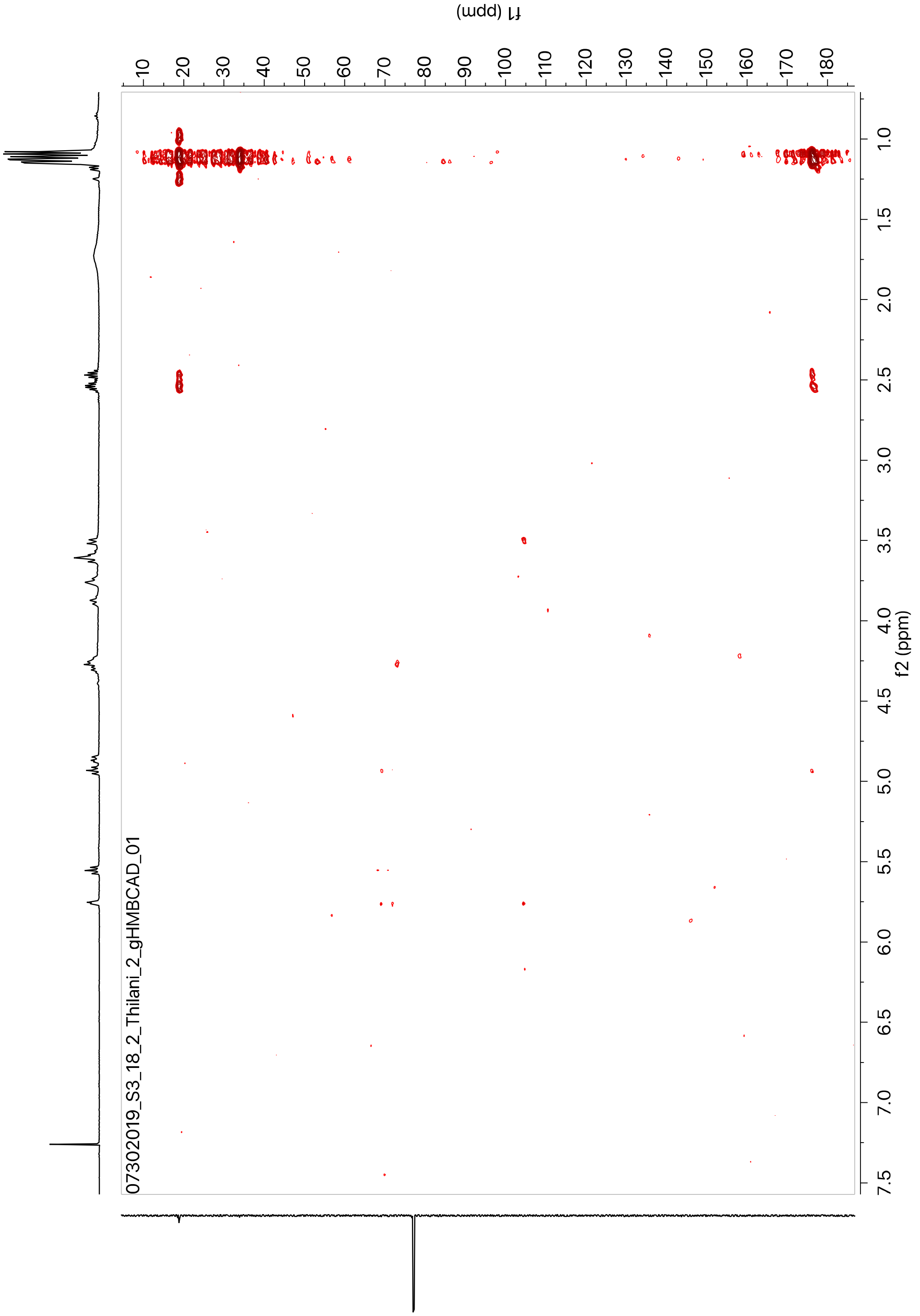

Figure S7 gHMBCAD NMR spectrum for S3:12(4,4,4) purified from *S. pennellii* LA0716.

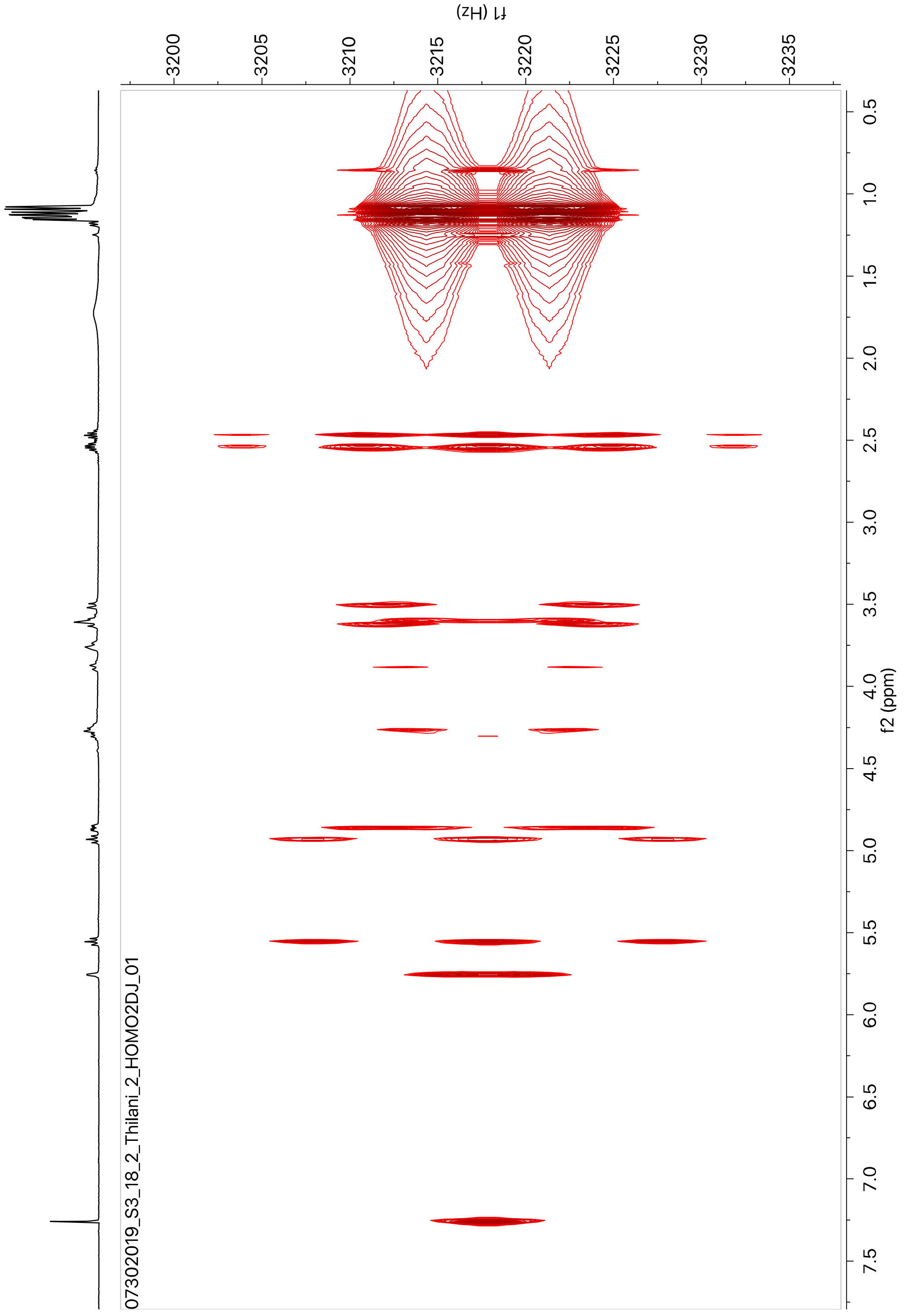

Figure S8 ^{1}H-^{1}H HOMO2DJ NMR spectrum for S3:12(4,4,4) purified from *S. pennellii* LA0716.

Table S7 NMR chemical shifts for S3:18(4,4,10)-1 purified from *S. pennellii* LA0716.

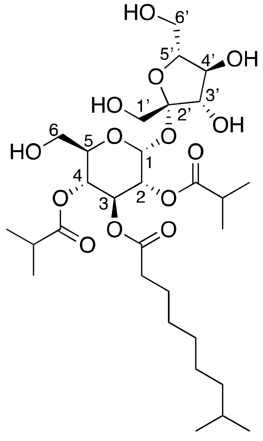

S3:18(4,4,10)-1

Purified from *S. pennellii* LA0716

Chemical Formula: $C_{30}H_{52}O_{14}$

HRMS: (ESI) *m/z* calculated for $C_{30}H_{52}O_{14}$ ($[M+NH_4]^+$): 654.3695

Experimental *m/z*: 654.3699

InChI Key: DGVGFAZINLIXDJ-ZCDLJYLNSA-N

NMR (500 MHz, $CDCl_3$)

Sample mass: 2 mg

**Carbon # (group)**	**^{1}H (ppm)**	**^{13}C (ppm)** (from HSQC and HMBC)
**1** (CH)	5.77 (d, *J* = 4.0 Hz, 1H)	88.79
**2** (CH)	4.84 (dd, *J* = 10.3, 4.0 Hz, 1H)	70.80
- 1 (CO)	-	176.59
- 2 (CH)	2.55 (hept, *J* = 7.0 Hz, 1H)	33.84
- 3,4 (CH_3)	1.13 (d, *J* = 7.0 Hz, 6H)	19.41
**3** (CH)	5.56 (dd, *J* = 10.3 Hz, 1H)	69.06
- 1 (CO)	-	172.84
- 2 (CH_2)	2.21 (t, J = 7.8 Hz, 2H)	34.28
- 3 (CH_2)	1.52(m, 2H)	24.83
- 4,5,6 (CH_2)	1.25 (m)	29.39
- 7 (CH_2)	1.13 (m)	38.93
- 8 (CH)	1.49 (m)	27.98
- 9,10 (CH_3)	0.85 (m)	22.65
**4** (CH)	4.91 (t, *J* = 10.4 Hz, 1H)	68.40
- 1 (CO)	-	176.16
- 2 (CH)	2.55 (hept, *J* = 7.0 Hz, 1H)	33.84
- 3,4 (CH_3)	1.13 (d, *J* = 7.0 Hz, 6H)	19.41
**5** (CH)	4.21 (m, 1H)	71.89
**6** (CH_2)	3.61 (m, 2H)	61.51
**1′** (CH_2)	3.60 (m, 1H), 3.52 (d, *J* = 12.0 Hz, 1H)	64.60
**2′** (C)	-	104.52
**3′** (CH)	4.25 (m, 1H)	78.18
**4′** (CH)	4.31 (t, *J* = 8.4 Hz, 2H)	72.89
**5′** (CH)	3.74 (m, 1H)	81.39
**6′** (CH_2)	3.87 (d, *J* = 13.0 Hz, 1H), 3.74 (m, 1H) -	60.02

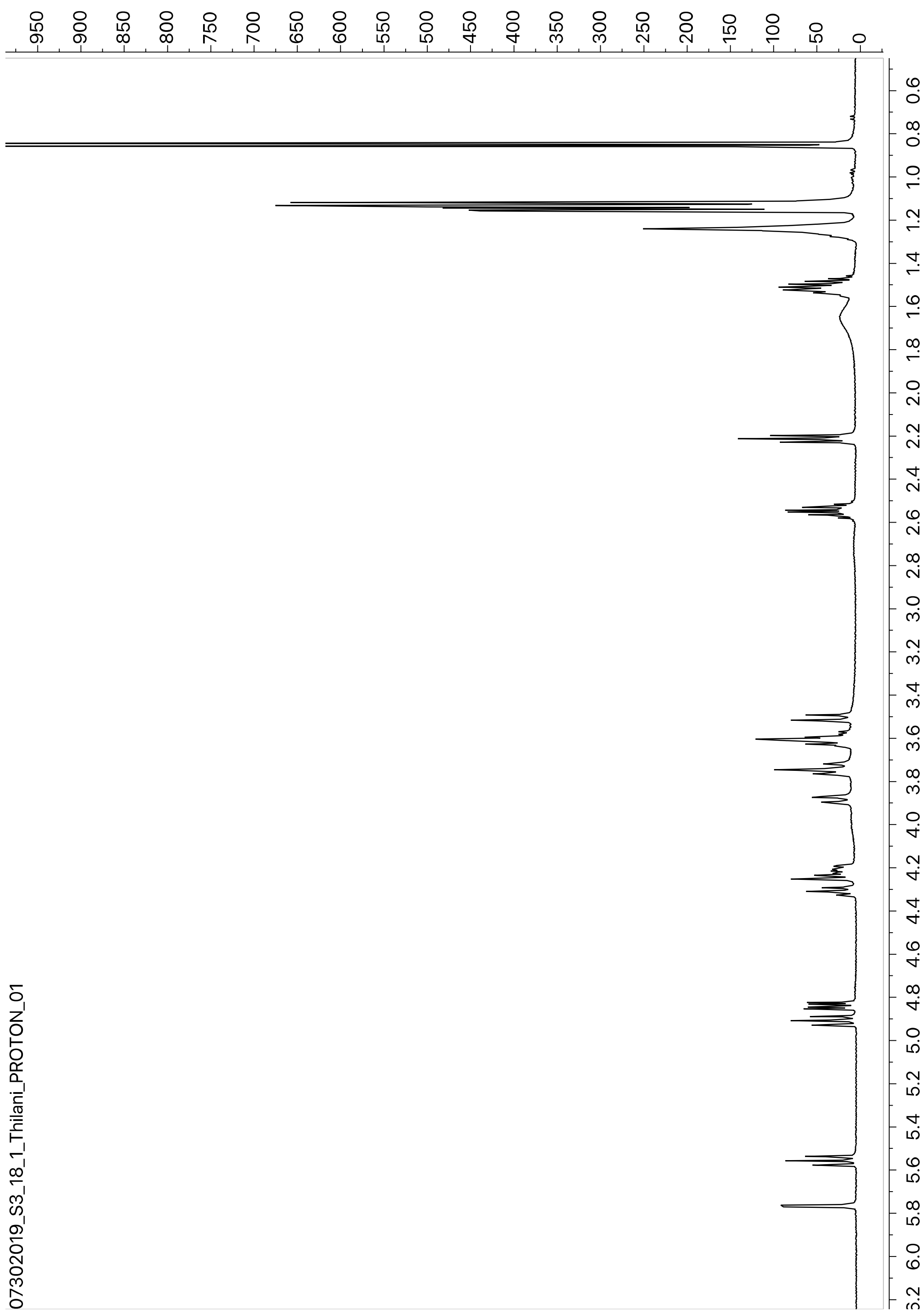

Figure S9 ^{1}H NMR spectrum for S3:18(4,4,10)-1 purified from *S. pennellii* LA0716.

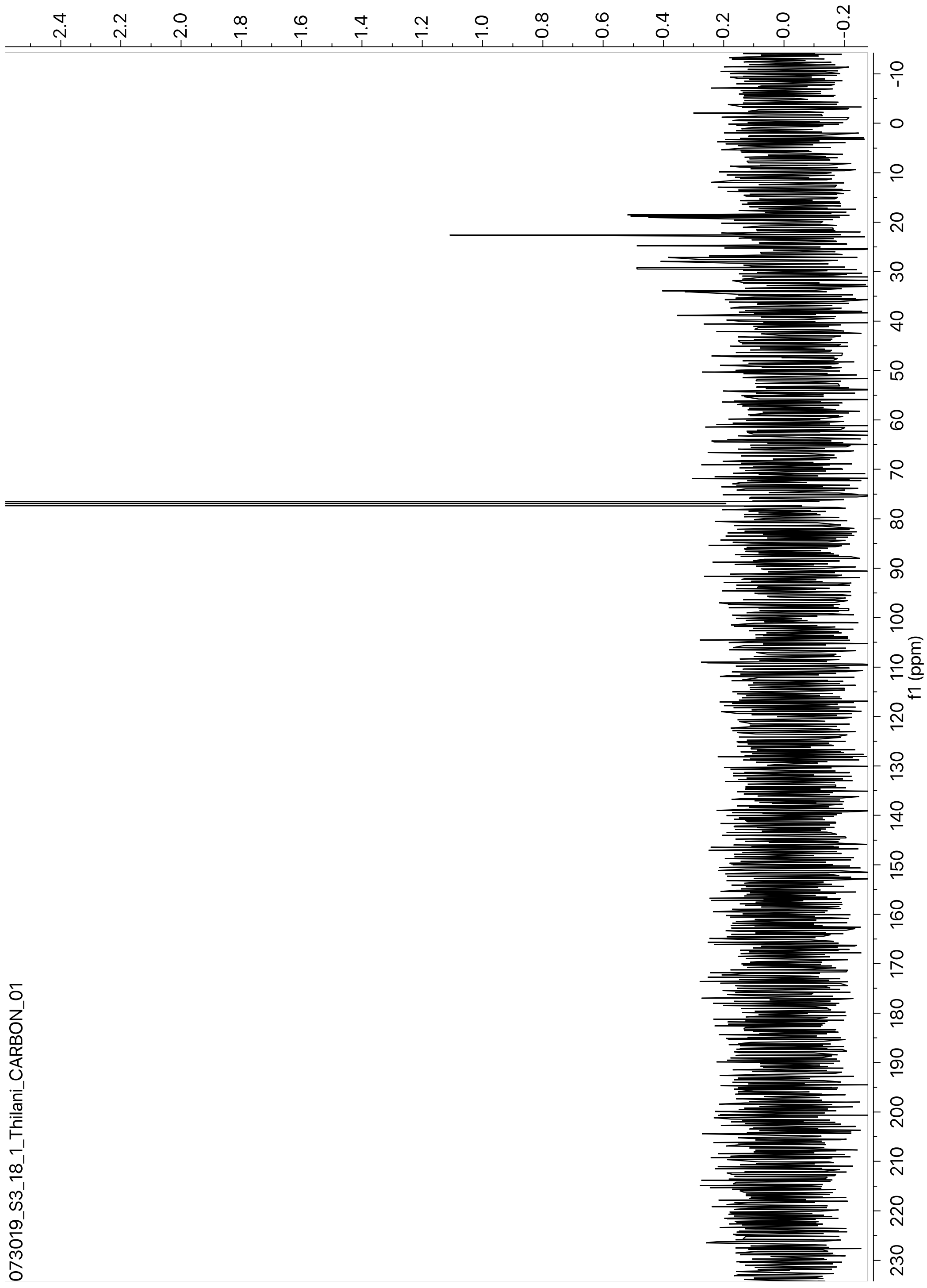

Figure S10 ^{13}C NMR spectrum for S3:18(4,4,10)-1 purified from *S. pennellii* LA0716.

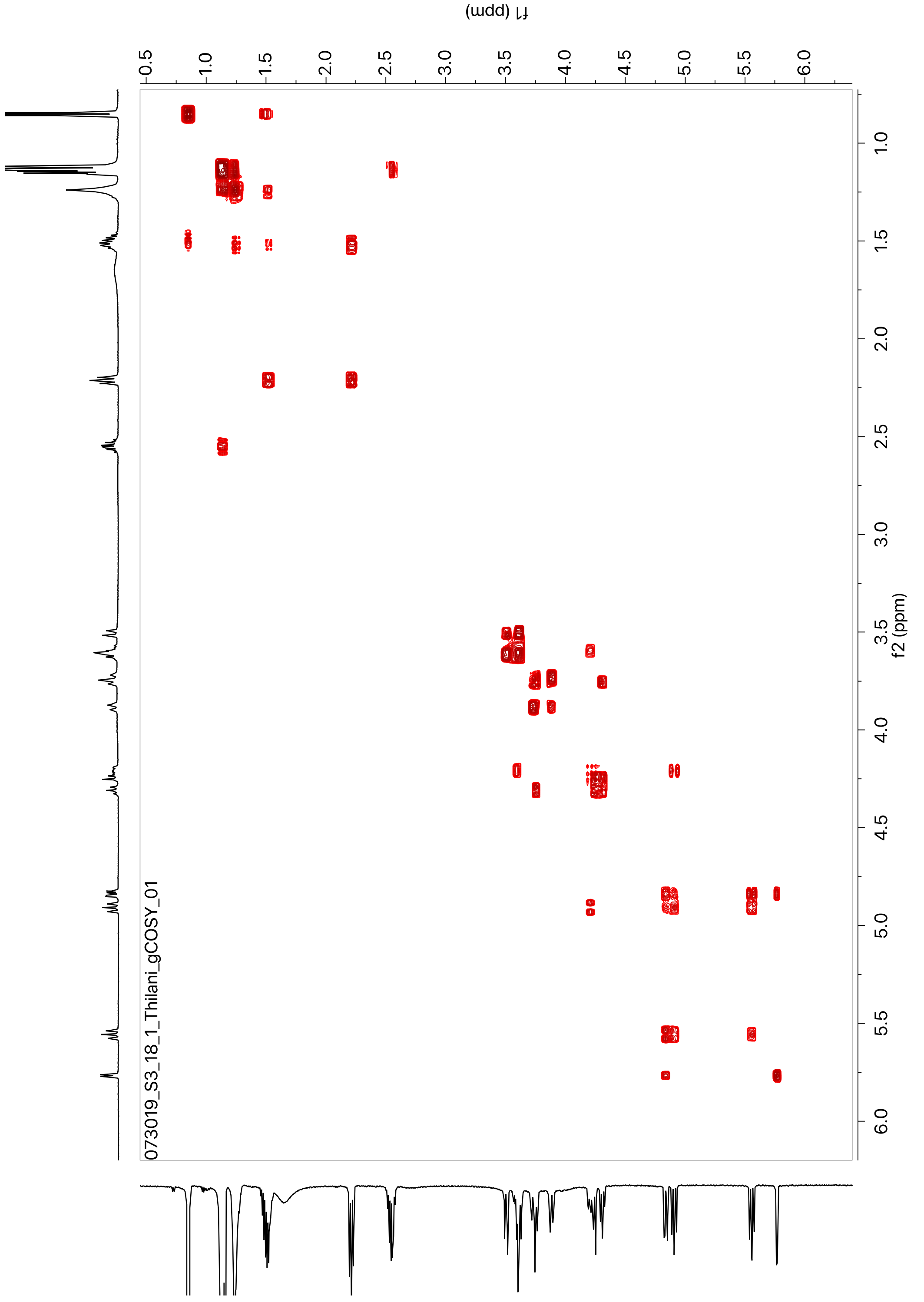

Figure S11 gCOSY NMR spectrum for S3:18(4,4,10)-1 purified from *S. pennellii* LA0716.

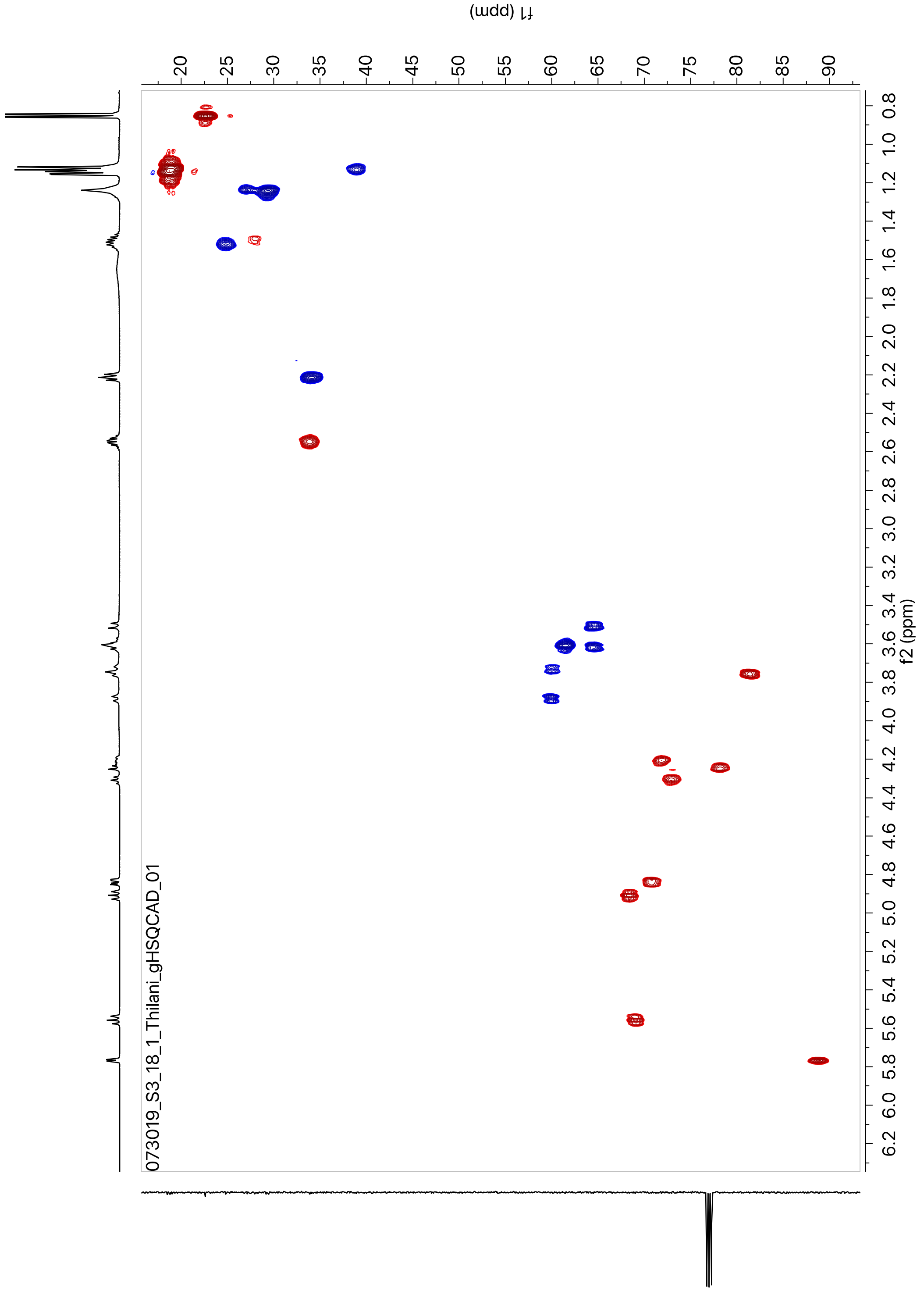

Figure S3.12 gHSQCAD NMR spectrum for S3:18(4,4,10)-1 purified from *S. pennellii* LA0716.

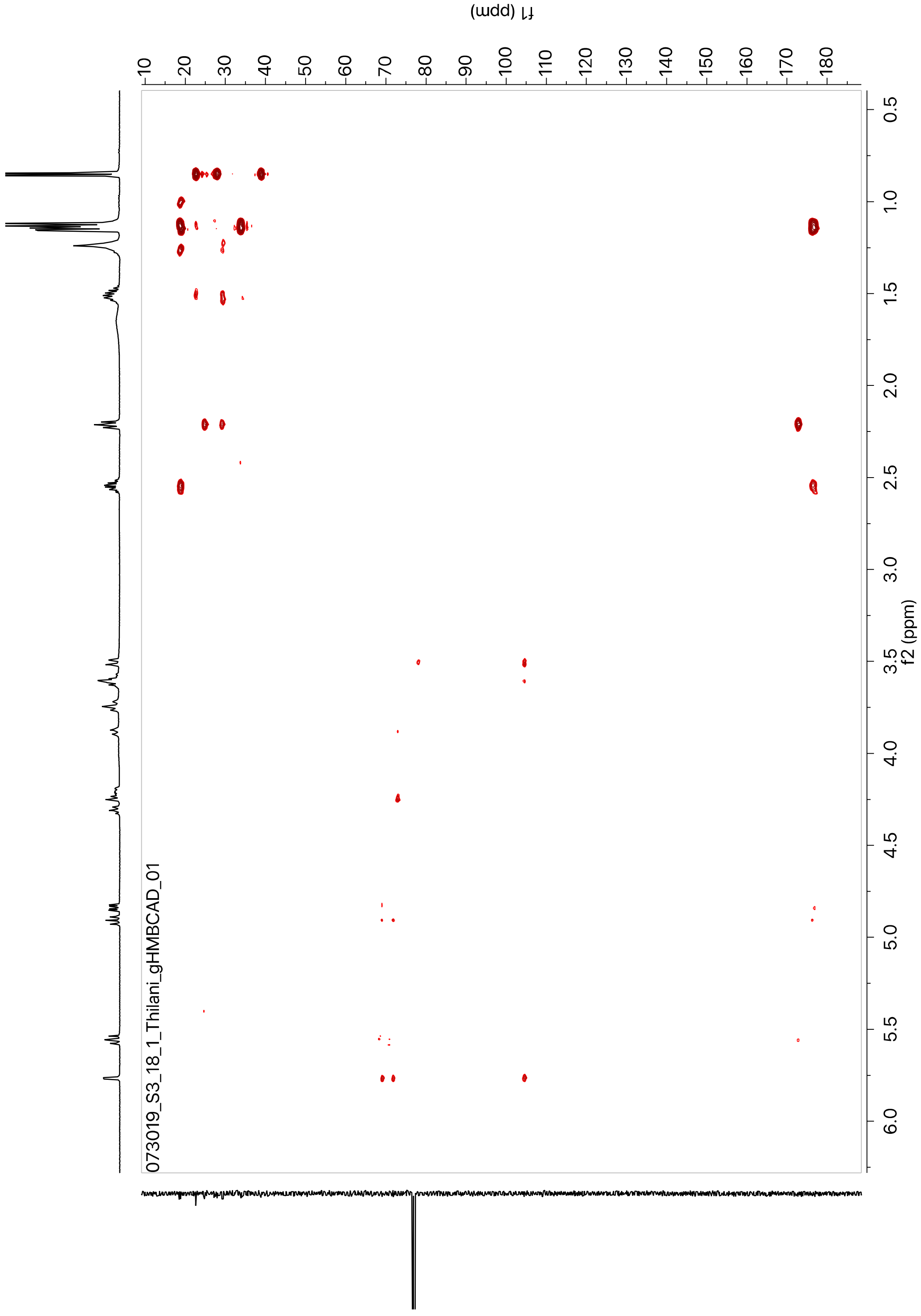

Figure S13 gHMBCAD NMR spectrum for S3:18(4,4,10)-1 purified from *S. pennellii* LA0716.

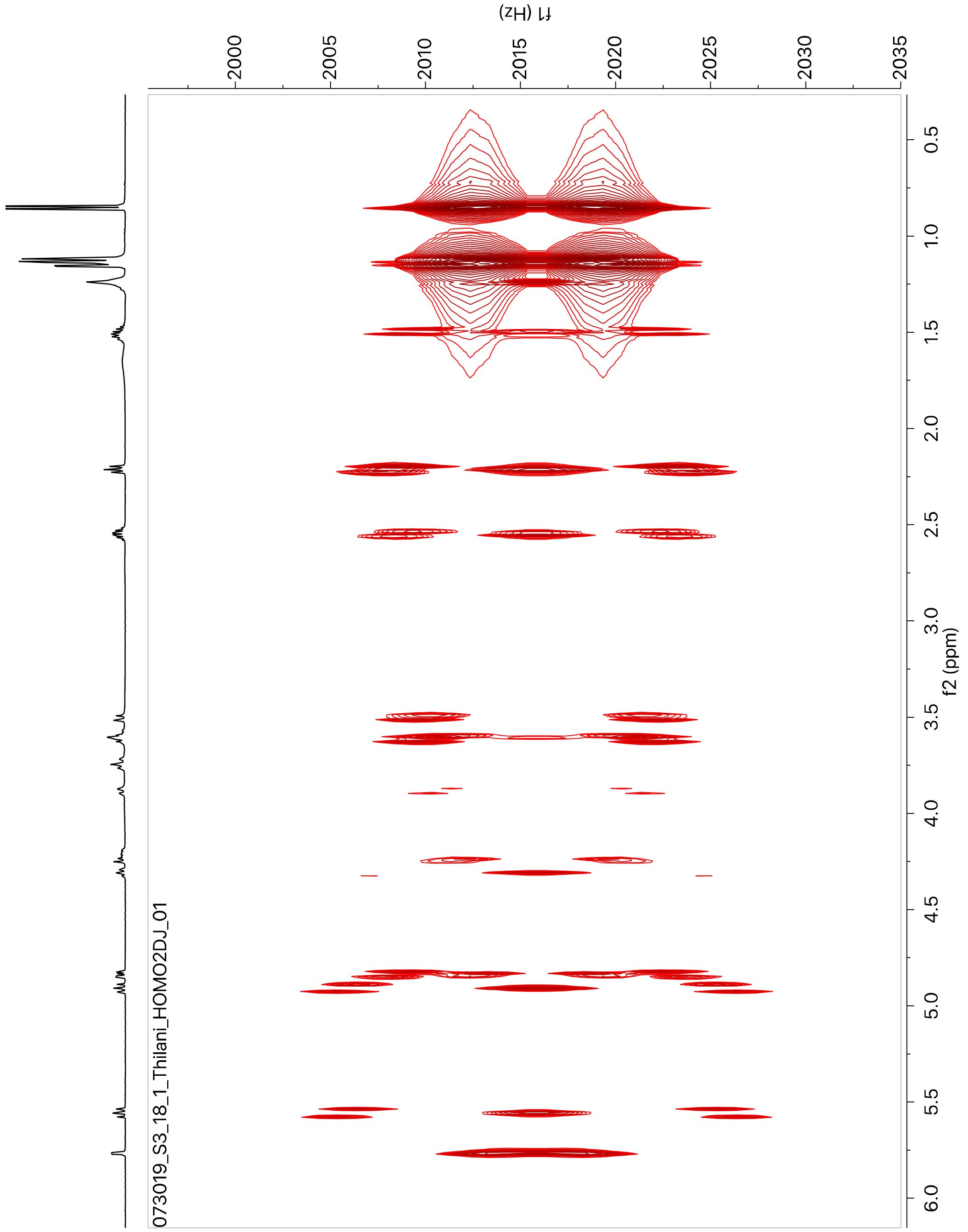

Figure S14 ^{1}H-^{1}H HOMO2DJ NMR spectrum for S3:18(4,4,10)-1 purified from *S. pennellii* LA0716.

Table S8 NMR chemical shifts for S3:18(4,4,10)-2 purified from *S. pennellii* LA0716.

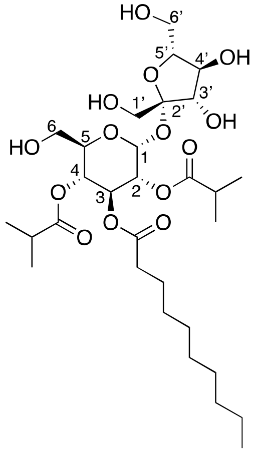

S3:18(4,4,10)-2

Purified from *S. pennellii* LA0716

Chemical Formula: $C_{30}H_{52}O_{14}$

HRMS: (ESI) *m/z* calculated for $C_{30}H_{52}O_{14}$ ($[M+NH_4]^+$): 654.3695

Experimental *m/z*: 654.3699

InChI Key: QCHCMGNIDJVBGL-ZCDLJYLNSA-N

NMR (500 MHz, $CDCl_3$)

Sample mass: 2 mg

**Carbon #<br>(group)**	**^{1}H (ppm)**	**^{13}C (ppm)**<br>(from HSQC and HMBC)
**1** (CH)	5.77 (d, *J* = 4.0 Hz, 1H)	88.81
**2** (CH)	4.83 (dd, *J* = 10.3, 4.0 Hz, 1H)	70.81
- 1 (CO)	-	176.89
- 2 (CH)	2.55 (hept, *J* = 7.0 Hz, 1H)	33.85
- 3,4 (CH_3)	1.13 (d, *J* = 7.0 Hz, 6H)	18.91
**3** (CH)	5.56 (dd, *J* = 10.0 Hz, 1H)	69.02
- 1 (CO)	-	172.70
- 2 (CH_2)	2.21 (t, J = 7.6 Hz, 2H)	34.11
- 3 (CH_2)	1.51 (pent, *J* = 7.1 Hz, 2H)	24.79
- 4,5,6,7 (CH_2)	1.24 (m)	29.31
- 8 (CH_2)	1.24 (m)	38.93
- 9 (CH_2)	1.27 (m)	22.56
- 10 (CH_3)	0.87 (t, *J* = 6.9 Hz, 3H)	14.06
**4** (CH)	4.90 (t, *J* = 10.4 Hz, 1H)	68.44
- 1 (CO)	-	176.16
- 2 (CH)	2.55 (hept, *J* = 7.0 Hz, 1H)	33.85
- 3,4 (CH_3)	1.13 (d, *J* = 7.0 Hz, 6H)	18.91
**5** (CH)	4.20 (m, 1H)	71.85
**6** (CH_2)	3.61 (m, 2H)	61.53
**1′** (CH_2)	3.60 (m, 1H), 3.51 (d, *J* = 12.0 Hz, 1H)	64.47
**2′** (C)	-	104.45
**3′** (CH)	4.25 (m, 1H)	78.27
**4′** (CH)	4.30 (t, *J* = 8.4 Hz, 2H)	72.99
**5′** (CH)	3.75 (m, 1H)	81.40
**6′** (CH_2)	3.89 (d, *J* = 13.0 Hz, 1H), 3.74 (m, 1H)<br>-	60.02

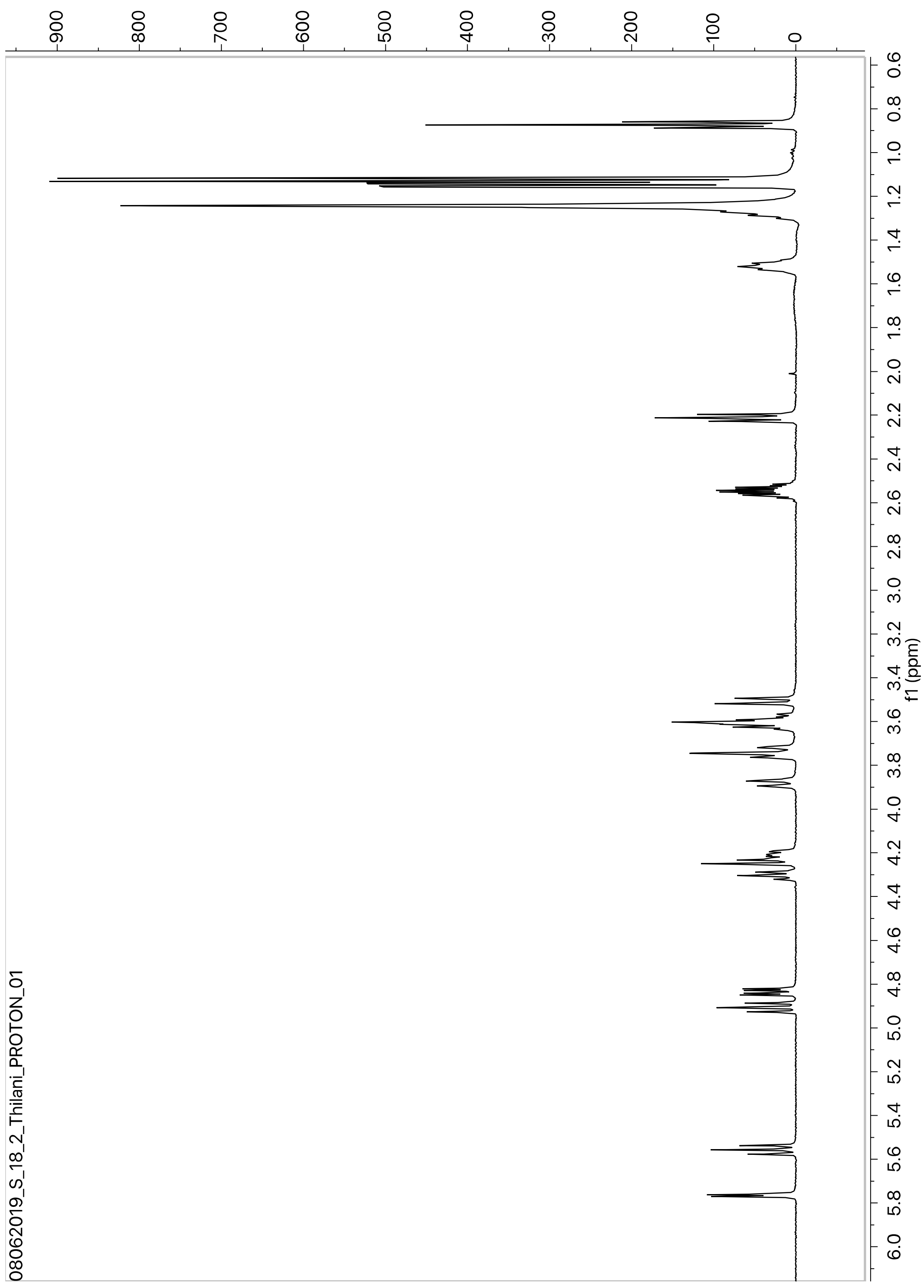

Figure S15 ^{1}H NMR spectrum for S3:18(4,4,10)-2 purified from *S. pennellii* LA0716.

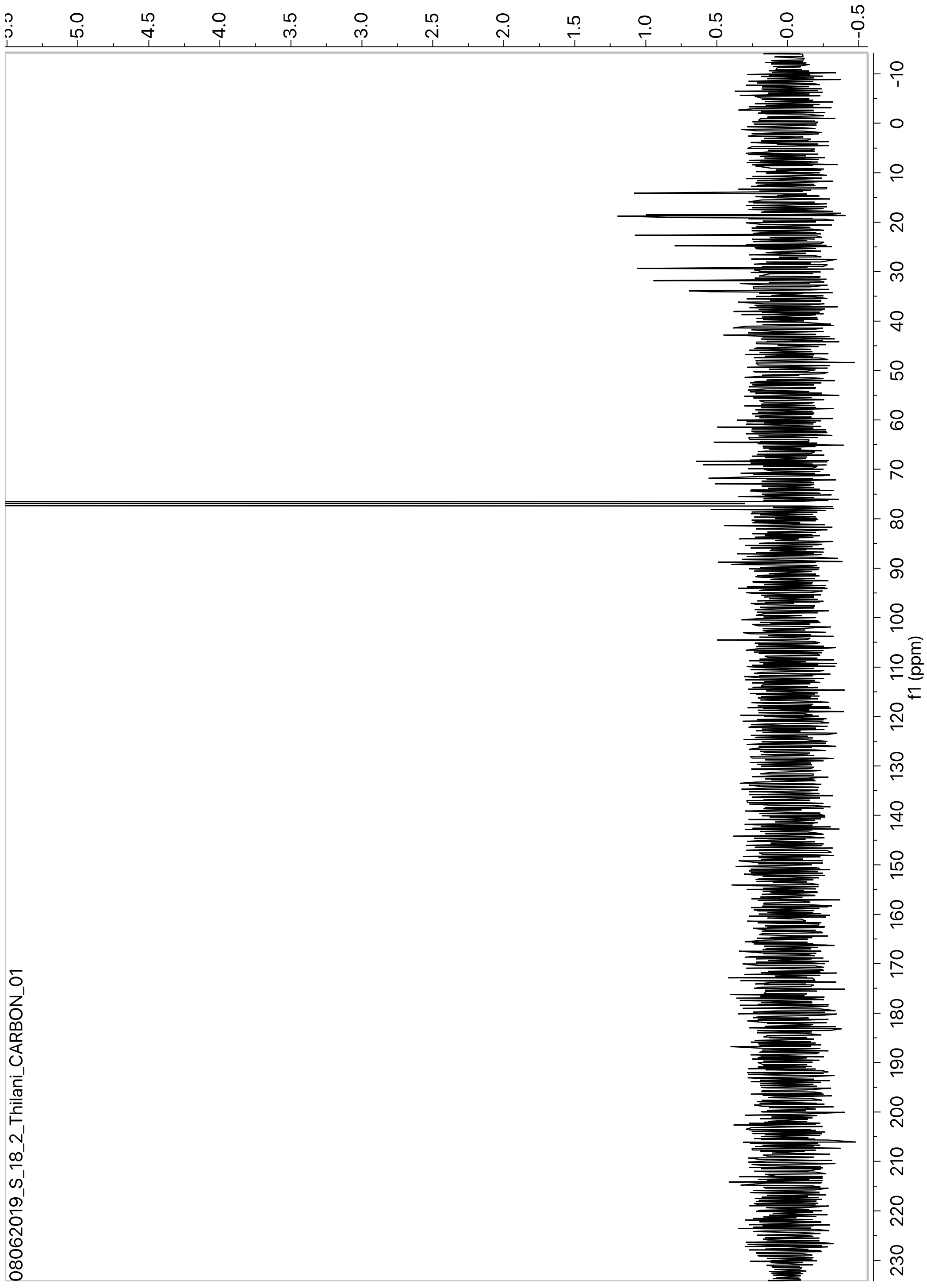

Figure S16 ^{13}C NMR spectrum for S3:18(4,4,10)-2 purified from *S. pennellii* LA0716.

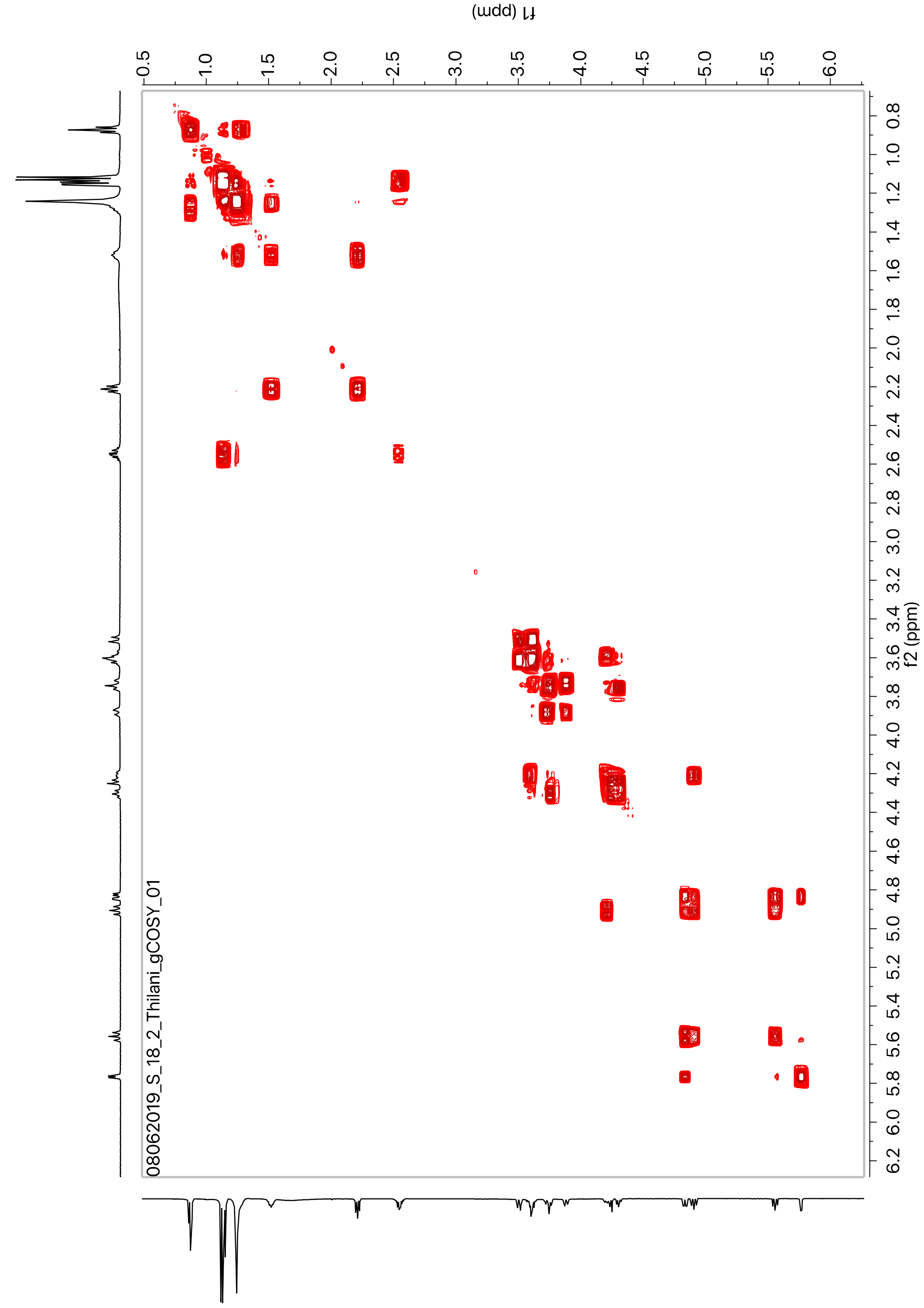

Figure S17 gCOSY NMR spectrum for S3:18(4,4,10)-2 purified from *S. pennellii* LA0716.

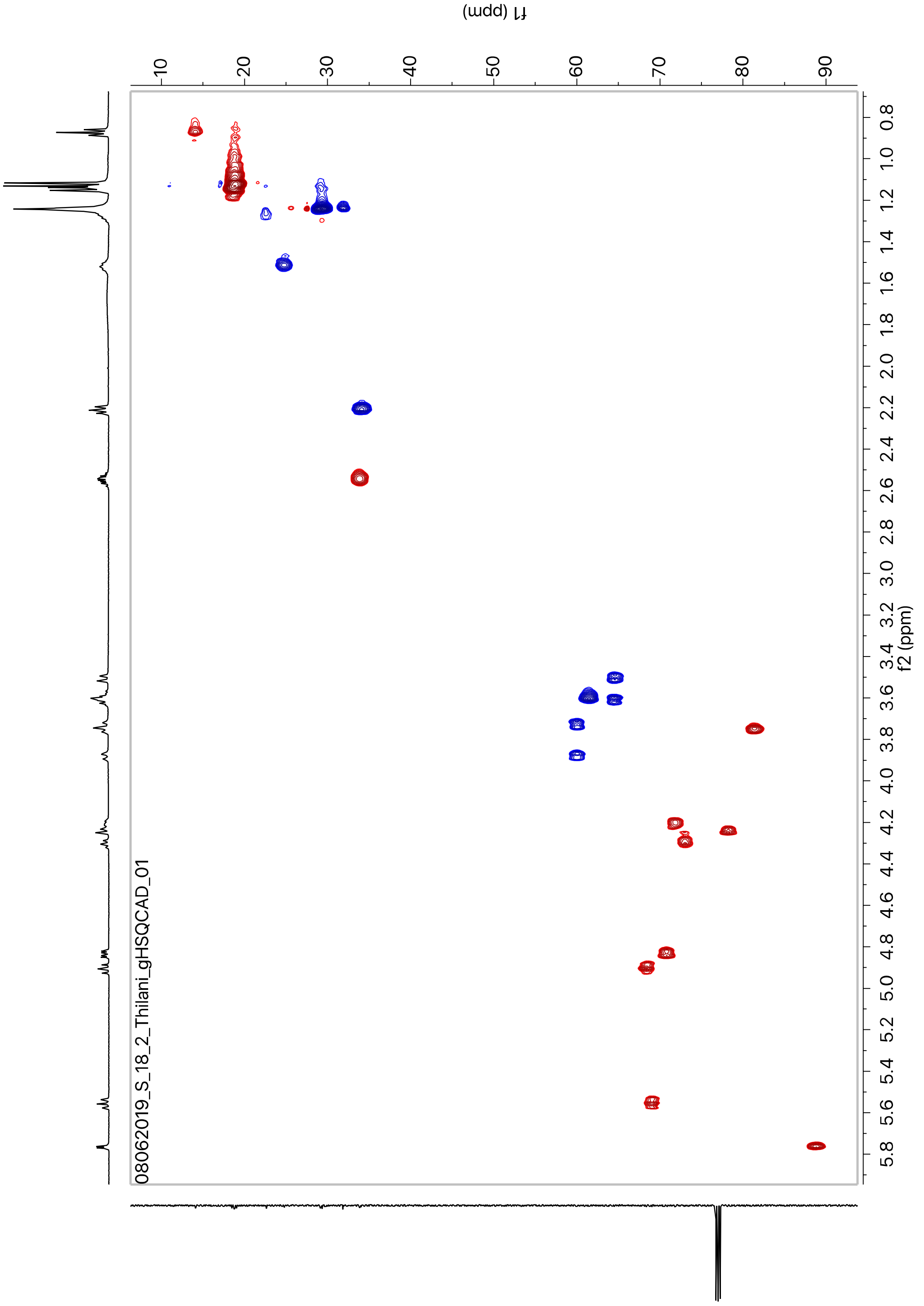

Figure S18 gHSQCAD NMR spectrum for S3:18(4,4,10)-2 purified from *S. pennellii* LA0716.

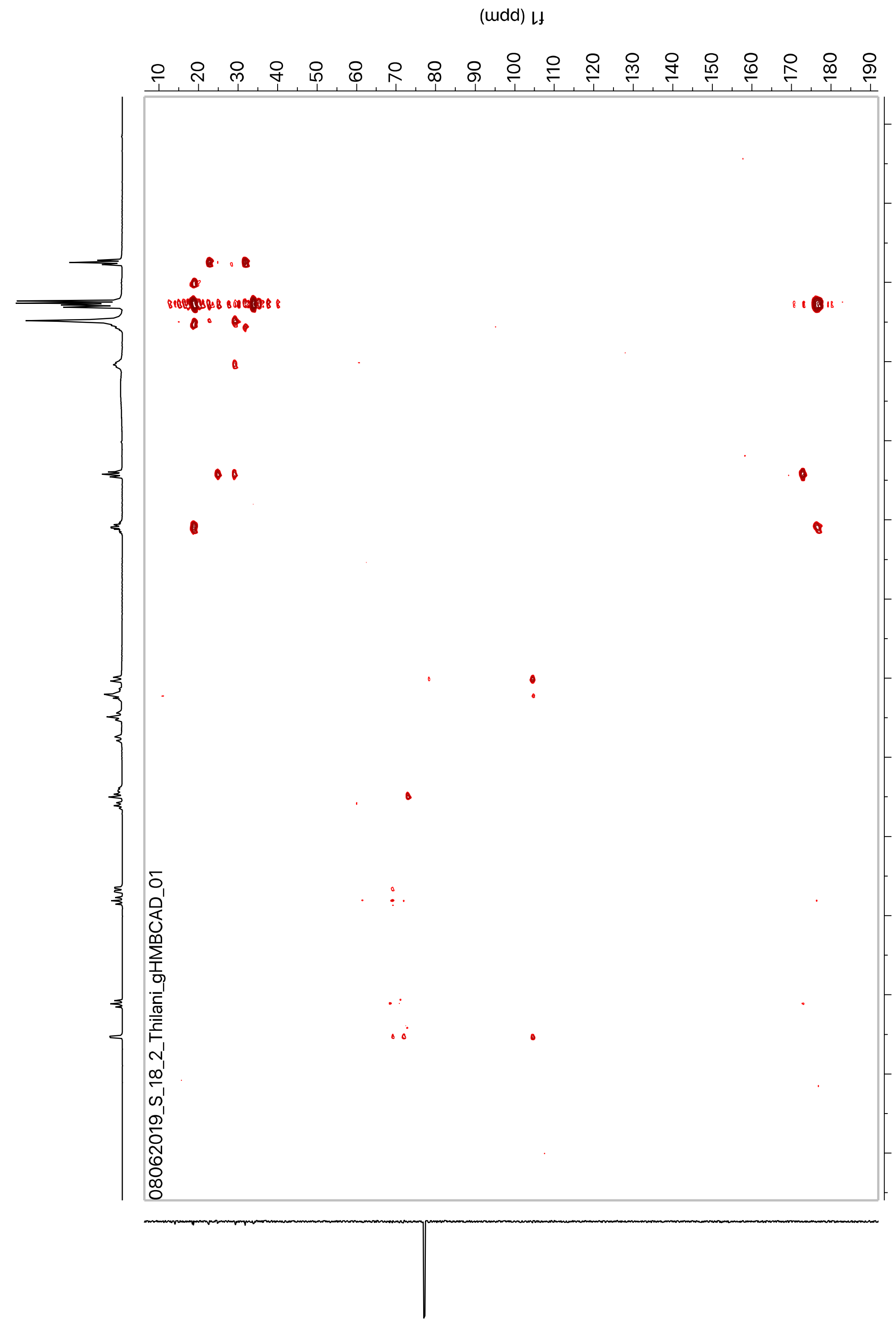

Figure S19 gHMBCAD NMR spectrum for S3:18(4,4,10)-2 purified from *S. pennellii* LA0716.

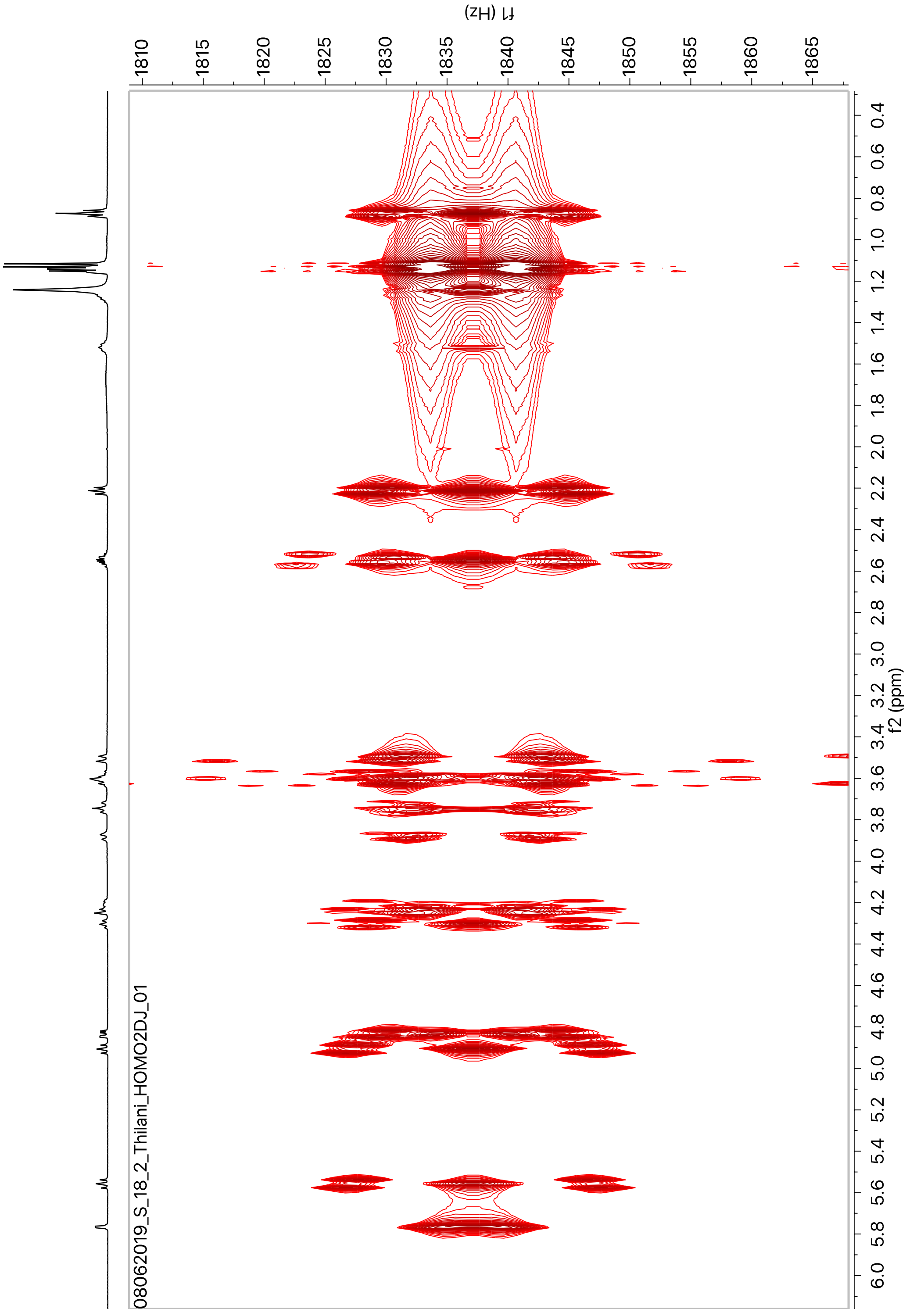

Figure S20 ^{1}H-^{1}H HOMO 2DJ NMR spectrum for S3:18(4,4,10)-2 purified from *S. pennellii* LA0716.

Table S9 NMR chemical shifts for S3:19(4,5,10)-1 purified from *S. pennellii* LA0716.

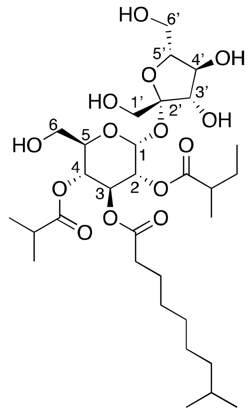	**S3:19(4,5,10)-1**<br><br>Purified from *S. pennellii* LA0716<br><br>Chemical Formula: $C_{31}H_{54}O_{14}$<br><br>HRMS: (ESI) *m/z* calculated for $C_{31}H_{54}O_{14}$ ($[M+NH_4]^+$): 668.3852<br><br>Experimental *m/z*: 668.3856<br><br>InChI Key: WWMDOPWHTJPJMW-KSMFYLDCSA-N<br><br>NMR (500 MHz, $CDCl_3$)<br><br>Sample mass: 2 mg

**Carbon #<br>(group)**	**^{1}H (ppm)**	**^{13}C (ppm)**<br>(from HSQC and HMBC)
**1** (CH)	5.78 (d, J = 4.0 Hz, 1H)	88.80
**2** (CH)	4.85 (dd, *J* = 10.4, 4.0 Hz, 1H)	70.84
- 1 (CO)	-	176.77
- 2 (CH)	2.40 (sextet, *J* = 6.8 Hz, 1H)	40.61
- 3 (CH_3)	1.12 (m, 3H)	16.08
- 4 (CH_2)	1.42, 1.60 (m, 2H)	26.78
- 5 (CH_3)	0.86 (t, *J* = 7.3 Hz, 3H)	11.42
**3** (CH)	5.56 (t, J = 10.0 Hz, 1H)	69.00
- 1 (CO)	-	173.04
- 2 (CH_2)	2.20 (t, J = 7.5 Hz, 2H)	34.16
- 3 (CH_2)	1.51(m, 2H)	24.68
- 4,5,6 (CH_2)	1.24 (m)	29.34
- 7 (CH_2)	1.13 (m)	38.82
- 8 (CH)	1.48 (m)	28.08
- 9,10 (CH_3)	0.85 (m)	22.71
**4** (CH)	4.90 (t, *J* = 10.0 Hz, 1H)	68.49
- 1 (CO)	-	176.16
- 2 (CH)	2.53 (hept, *J* = 7.0 Hz, 1H)	34.03
- 3,4 (CH_3)	1.11 (d, *J* = 7.0 Hz, 6H)	18.85
**5** (CH)	4.20 (m, 1H)	71.85
**6** (CH_2)	3.60, 3.60 (m, 2H)	61.55
**1′** (CH_2)	3.61 (m, 1H), 3.51 (d, *J* = 11.9 Hz, 2H)	64.60
**2′** (C)	-	104.52
**3′** (CH)	4.24 (m, 1H)	78.07
**4′** (CH)	4.30 (t, *J* = 8.5 Hz, 1H)	72.96
**5′** (CH)	3.74 (m, 1H)	81.35
**6′** (CH_2)	3.88 (d, *J* = 13.1 Hz, 1H), 3.74 (m, 1H)	60.06

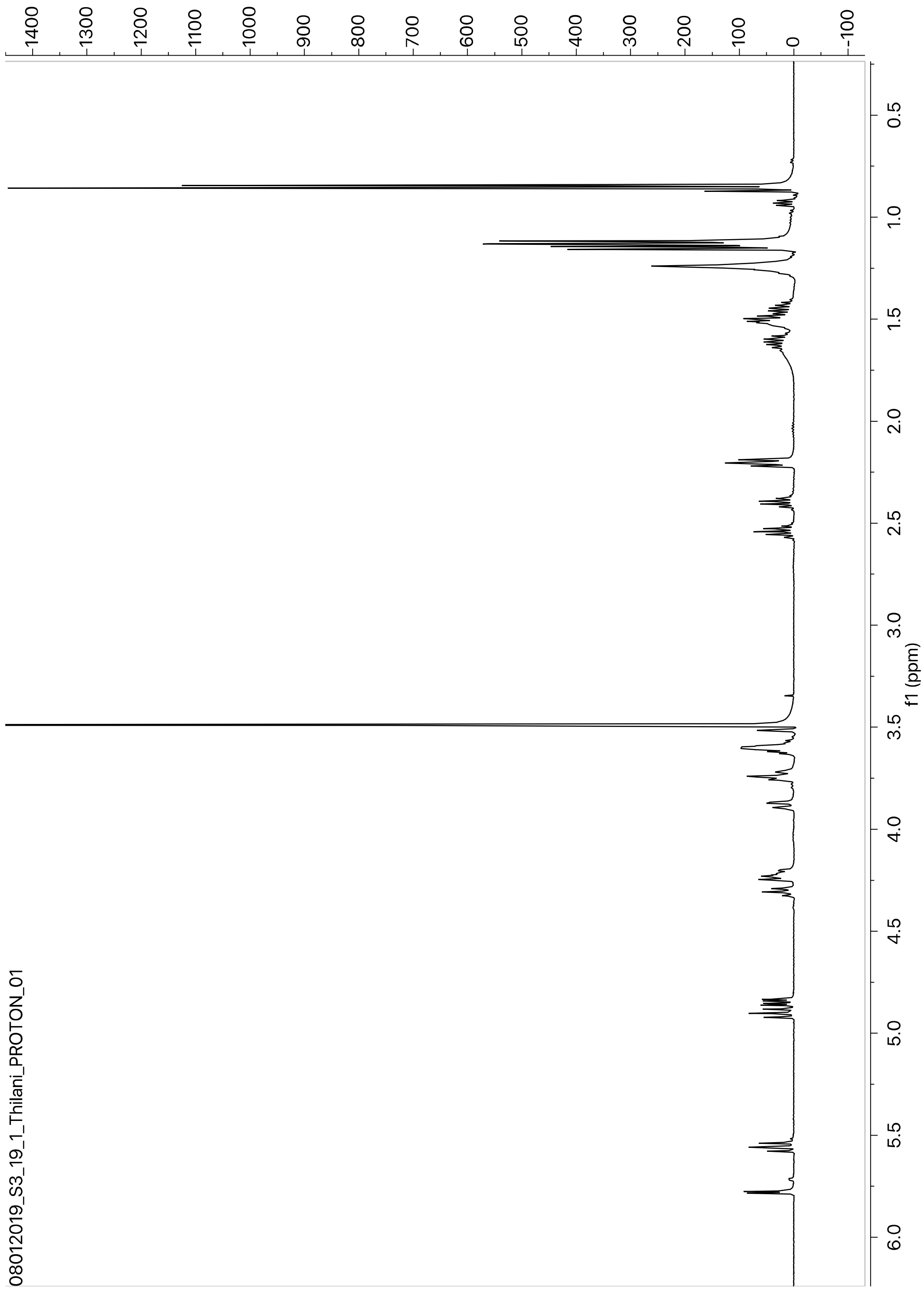

Figure S21 ^{1}H NMR spectrum for S3:19(4,5,10)-1 purified from *S. pennellii* LA0716.

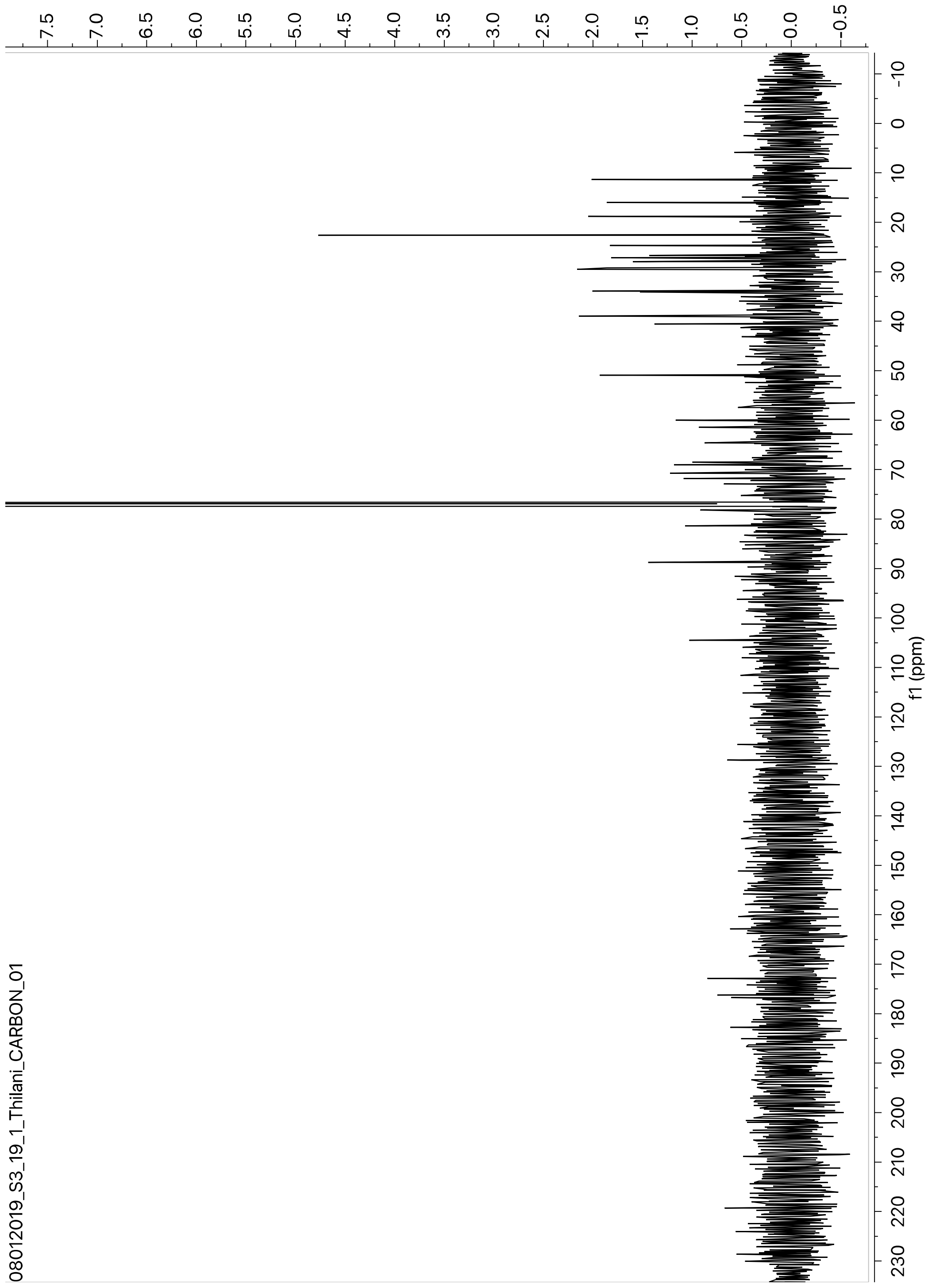

Figure S22 ^{13}C NMR spectrum for S3:19(4,5,10)-1 purified from *S. pennellii* LA0716.

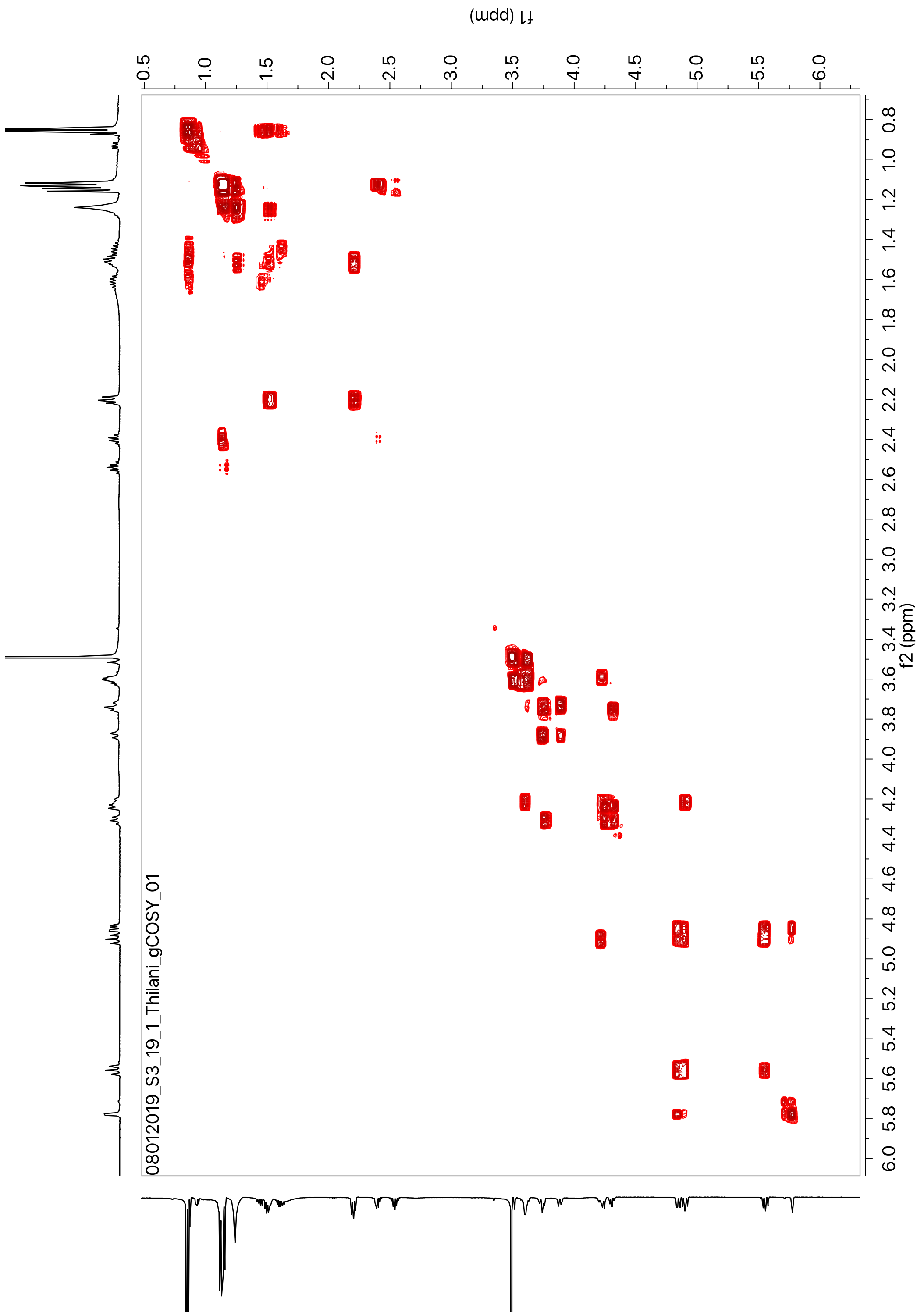

Figure S23 gCOSY NMR spectrum for S3:19(4,5,10)-1 purified from *S. pennellii* LA0716.

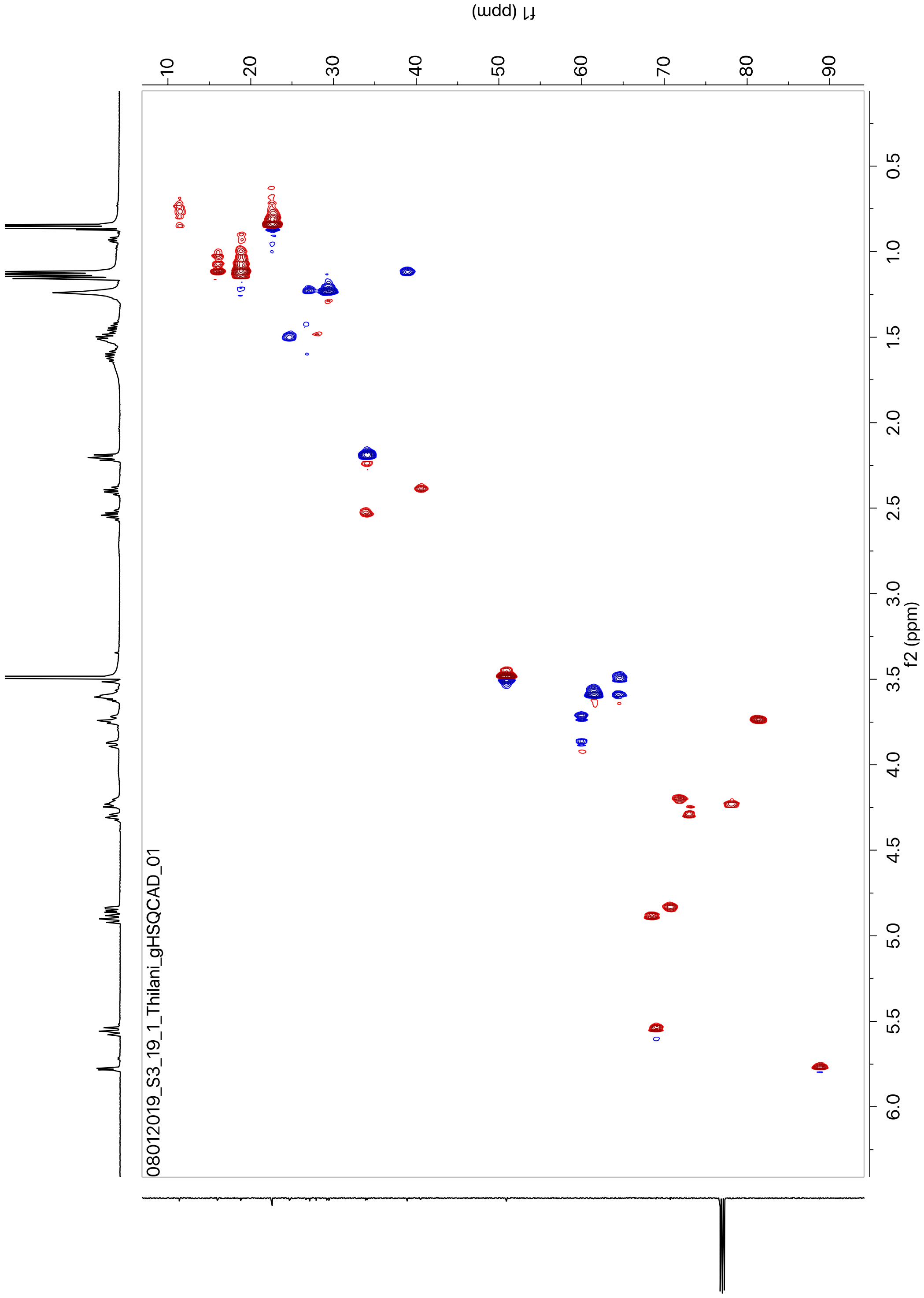

Figure S24 gHSQCAD NMR spectrum for S3:19(4,5,10)-1 purified from *S. pennellii* LA0716.

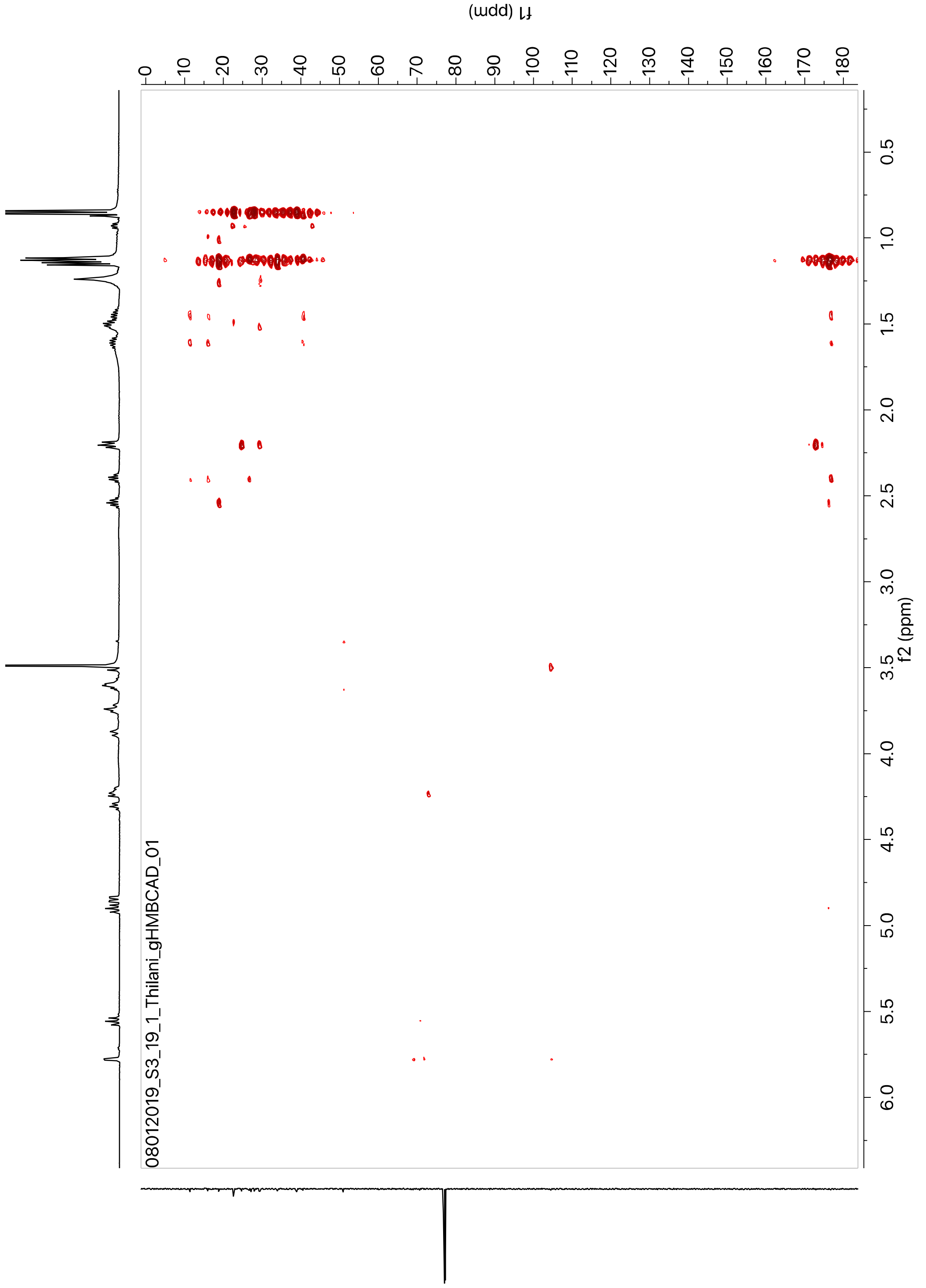

Figure S25 gHMBCAD NMR spectrum for S3:19(4,5,10)-1 purified from *S. pennellii* LA0716.

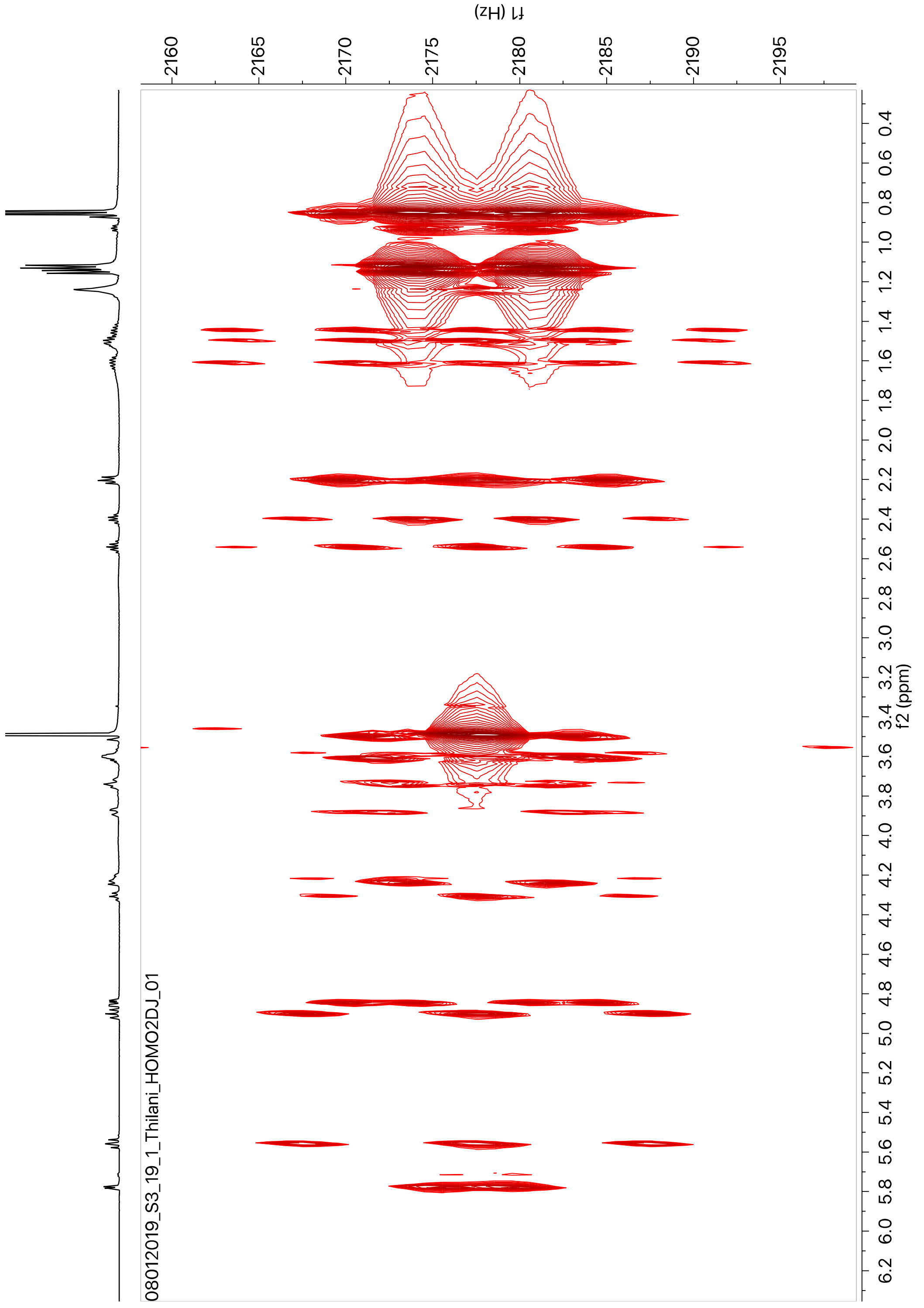

Figure S26 ^{1}H-^{1}H HOMO2DJ NMR spectrum for S3:19(4,5,10)-1 purified from *S. pennellii* LA0716.

Table S10 NMR chemical shifts for S3:19(4,5,10)-2 purified from *S. pennellii* LA0716.

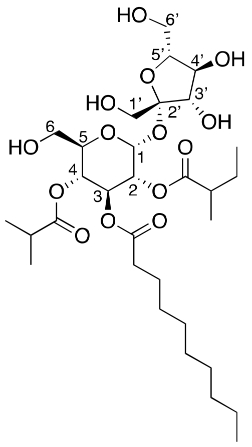	**S3:19(4,5,10)-2**<br>Purified from *S. pennellii* LA0716<br>Chemical Formula: $C_{31}H_{54}O_{14}$<br>HRMS: (ESI) *m/z* calculated for $C_{31}H_{54}O_{14}$ ($[M+NH_4]^+$): 668.3852<br>Experimental *m/z*: 668.3855<br>InChI Key: GPWDXGGZLQIWLR-KSMFYLDCSA-N<br>NMR (500 MHz, $CDCl_3$)<br>Sample mass: 2 mg

**Carbon #<br>(group)**	**^{1}H (ppm)**	**^{13}C (ppm)**<br>(from HSQC and HMBC)
**1** (CH)	5.78 (d, J = 4.0 Hz, 1H)	88.86
**2** (CH)	4.85 (dd, J = 10.4, 4.0 Hz, 1H)	70.68
- 1 (CO)	-	176.81
- 2 (CH)	2.40 (sextet, J = 6.8 Hz, 1H)	40.57
- 3 (CH_3)	1.13 (m, 3H)	16.03
- 4 (CH_2)	1.45, 1.61 (m, 2H)	26.62
- 5 (CH_3)	0.87 (t, J = 7.3 Hz, 3H)	11.36
**3** (CH)	5.56 (t, J = 10.0 Hz, 1H)	68.48
- 1 (CO)	-	172.64
- 2 (CH_2)	2.20 (t, J = 7.5 Hz, 2H)	34.07
- 3 (CH_2)	1.51(m, 2H)	24.75
- 4,5,6,7 (CH_2)	1.25 (m)	29.26
- 8 (CH_2)	1.24 (m)	31.92
- 9 (CH_2)	1.25 (m)	22.49
- 10 (CH_3)	0.87 (m)	14.13
**4** (CH)	4.91 (t, J = 10.0 Hz, 1H)	68.48
- 1 (CO)	-	176.16
- 2 (CH)	2.54 (hept, J = 6.84 Hz, 1H)	33.86
- 3,4 (CH_3)	1.13 (d, J = 6.84 Hz, 6H)	18.82
**5** (CH)	4.20 (m, 1H)	71.92
**6** (CH_2)	3.60, 3.60 (m, 2H)	61.51
**1′** (CH_2)	3.61, 3.51 (d, J = 11.9 Hz, 2H)	64.56
**2′** (C)	-	104.45
**3′** (CH)	4.24 (m, 1H)	78.32
**4′** (CH)	4.31 (t, J = 8.4 Hz, 2H)	72.98
**5′** (CH)	3.75 (m, 1H)	81.43
**6′** (CH_2)	3.87 (d, J = 13.1 Hz, 1H), 3.74 (m, 1H)<br>-	60.09

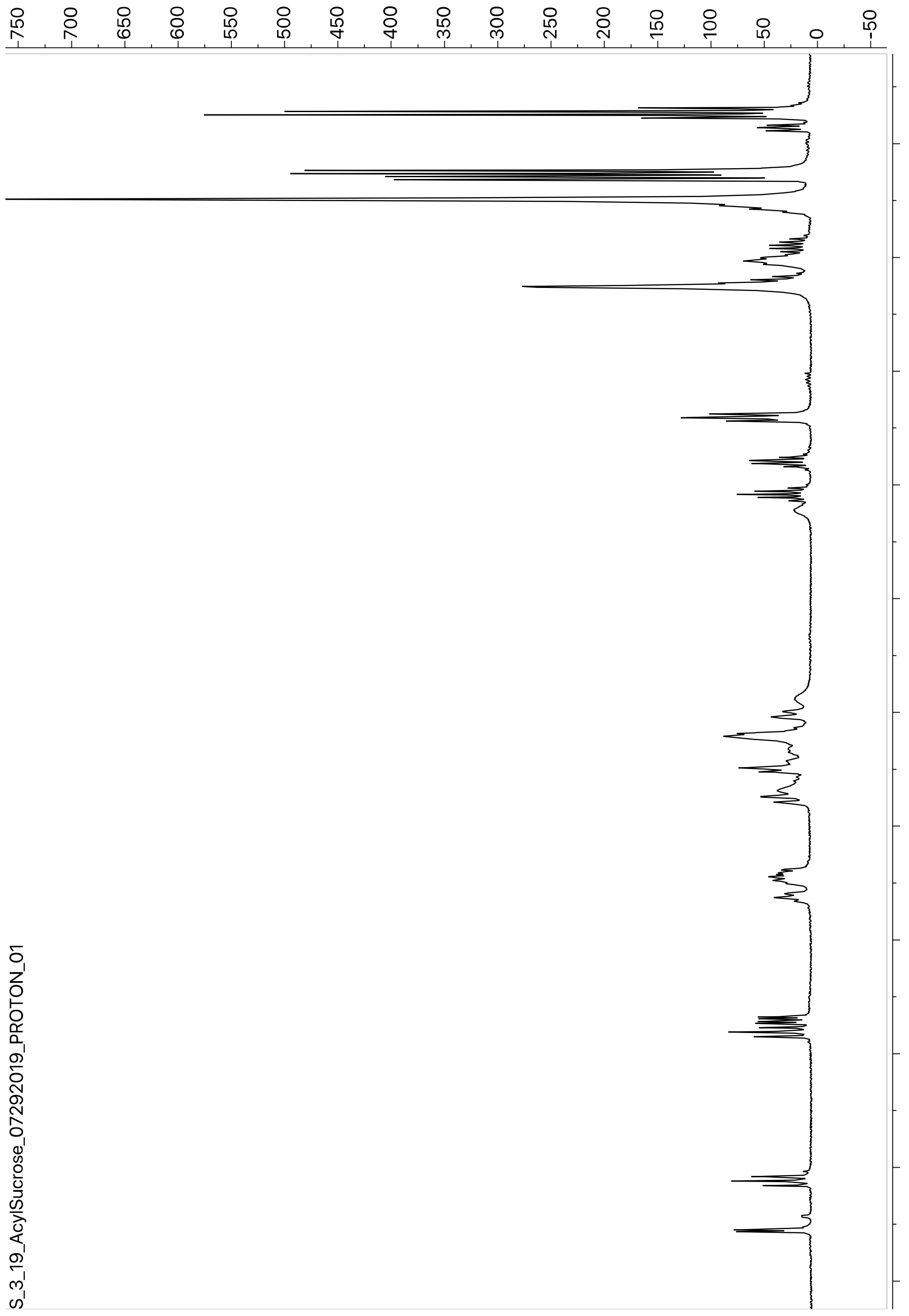

Figure S27 ^{1}H NMR spectrum for S3:19(4,5,10)-2 purified from *S. pennellii* LA0716.

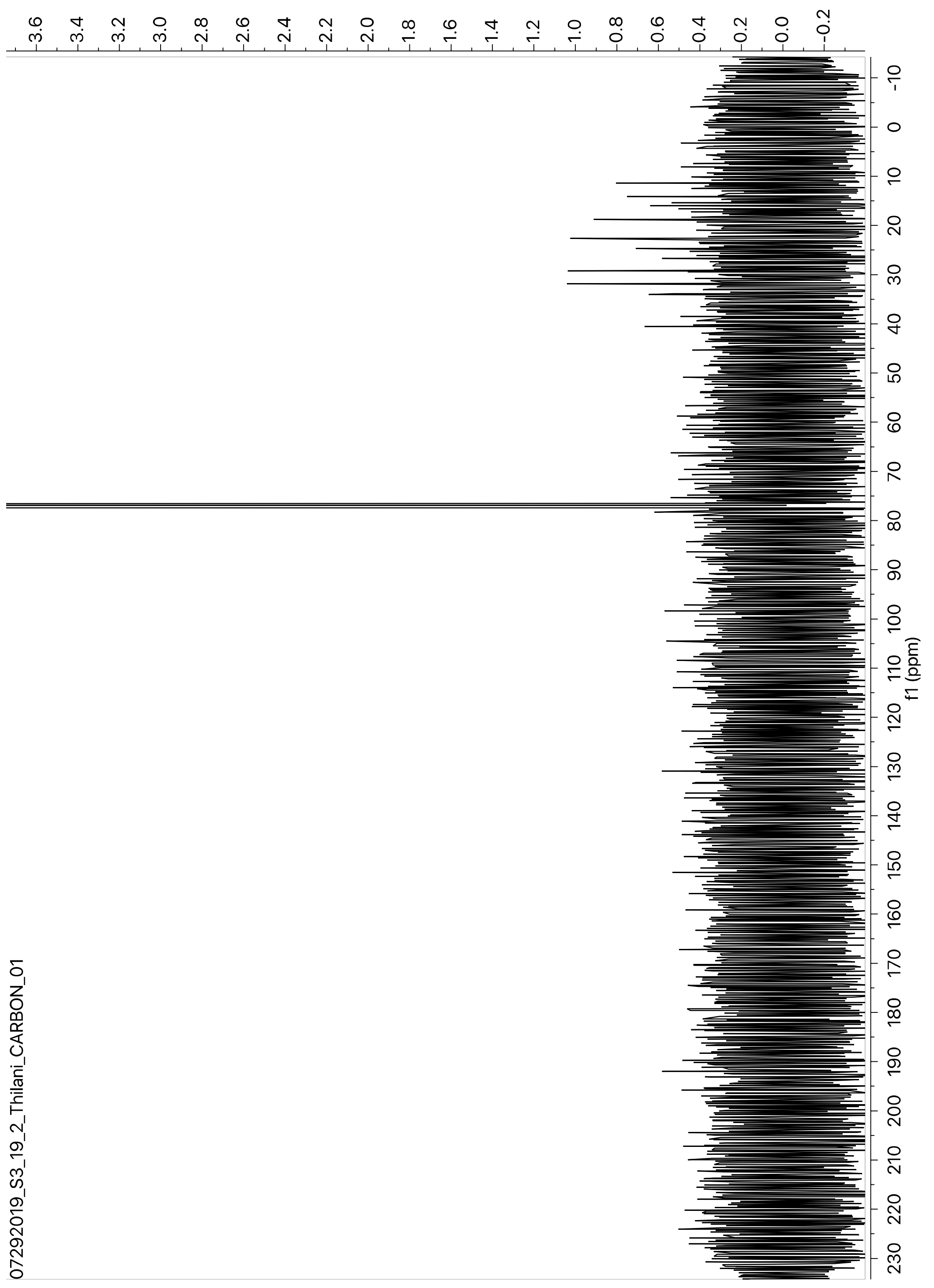

Figure S28 ^{13}C NMR spectrum for S3:19(4,5,10)-2 purified from *S. pennellii* LA0716.

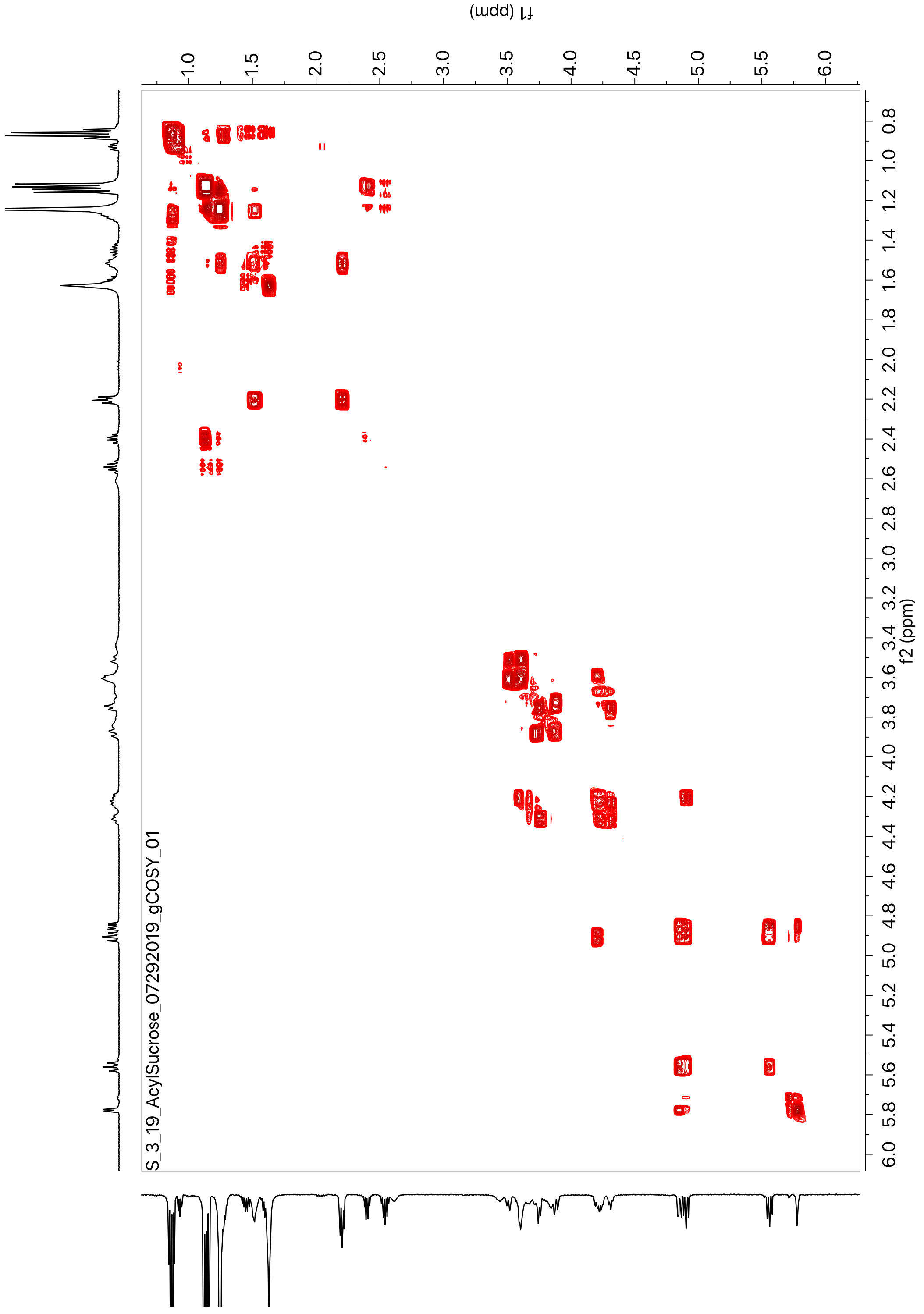

Figure S29 gCOSY NMR spectrum for S3:19(4,5,10)-2 purified from *S. pennellii* LA0716.

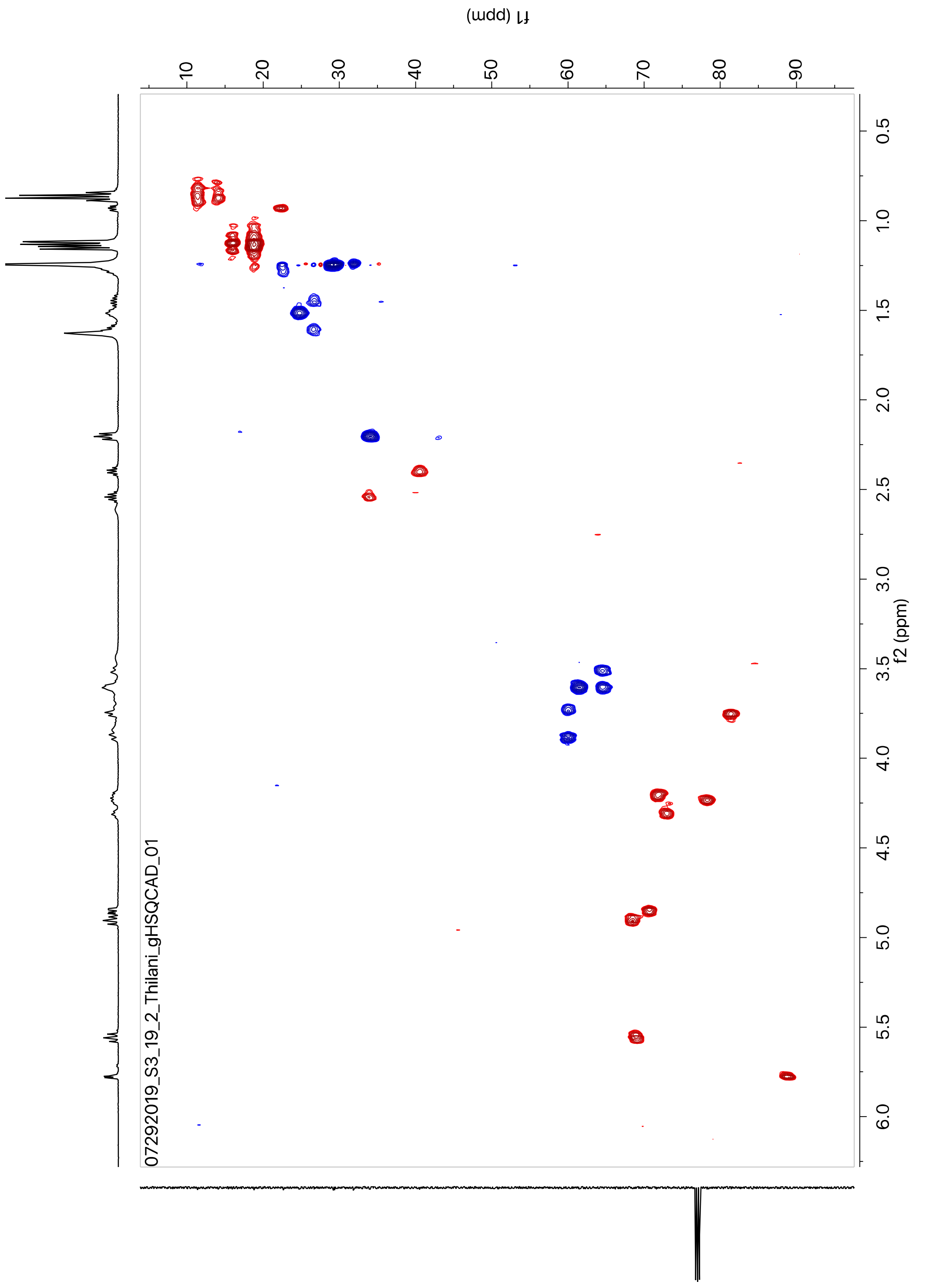

Figure S30 gHSQCAD NMR spectrum for S3:19(4,5,10)-2 purified from *S. pennellii* LA0716.

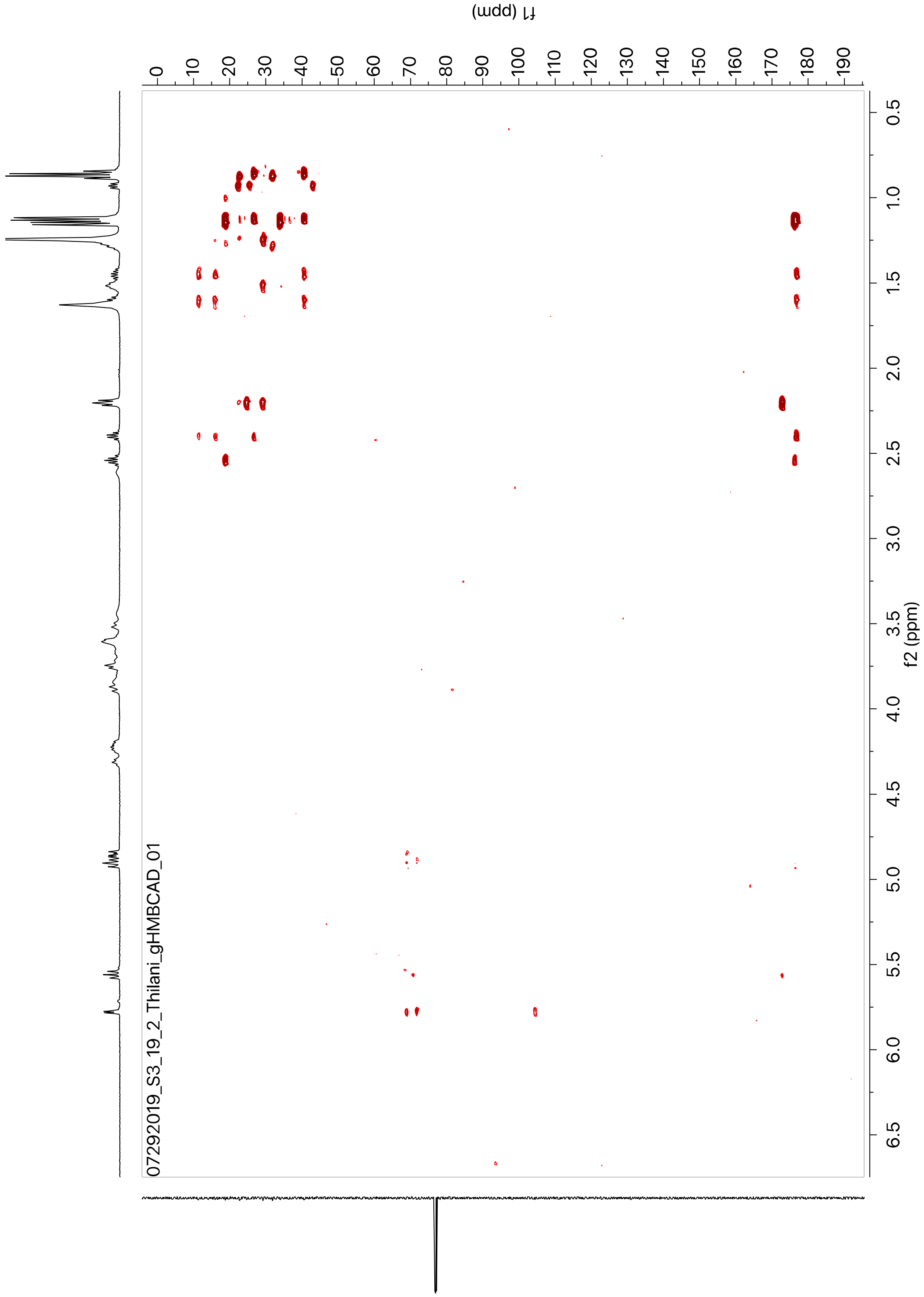

Figure S31 gHMBCAD NMR spectrum for S3:19(4,5,10)-2 purified from *S. pennellii* LA0716.

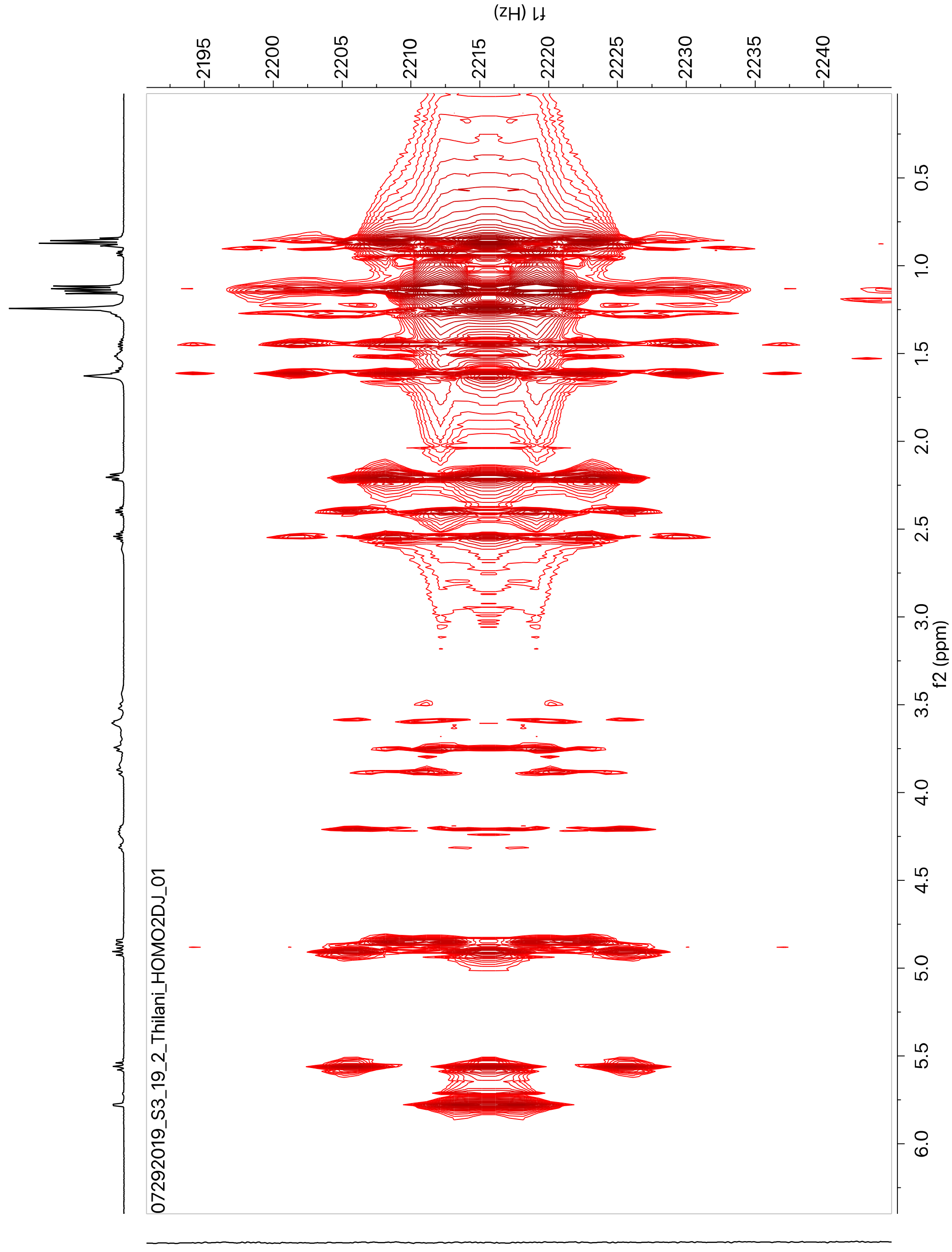

Figure S32 ^{1}H-^{1}H HOMO 2DJ NMR spectrum for S3:19(4,5,10)-2 purified from *S. pennellii* LA0716.

Table S11 NMR chemical shifts for G3:12(4,4,4) purified from *S. pennellii* LA0716.

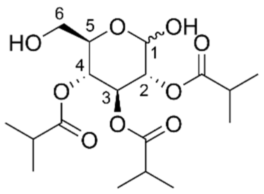

G3:12(4,4,4)

Purified from *S. pennellii* LA0716

Chemical Formula: $C_{18}H_{30}O_9$

HRMS: (ESI) *m/z* calculated for $C_{18}H_{30}O_9$ ($[M+NH_4]^+$): 408.2228

Experimental *m/z*: 408.2235

InChI Key: NVEWKQZGZJWFKH-SXHVGMSVSA-N
InChI Key (α): NVEWKQZGZJWFKH-VKNNWULWSA-N
InChI Key (b): NVEWKQZGZJWFKH-MSGZUBATSA-N

NMR (500 MHz, $CDCl_3$)

Sample mass: 2 mg

Carbon # (group)	^{1}H (ppm)		^{13}C (ppm) (from HSQC and HMBC)	
	α	β	α	β
**1** (CH)	5.49 (d, *J* = 3.7 Hz)	4.74 (d, *J* = 7.6 Hz)	90.28	95.75
**2** (CH)	4.91 (m)	4.89 (m)	71.06	73.30
- 1 (CO)	-	-	175.83	175.83
- 2 (CH)	2.49 (hept, *J* = 7.0 Hz)	2.49 (hept, *J* = 7.0 Hz)	33.93	33.93
- 3,4 (CH_3)	1.10 (m)	1.10 (m)	18.87	18.87
**3** (CH)	5.67 (t, *J* = 9.9 Hz)	5.39 (t, *J* = 9.7 Hz)	68.67	71.21
- 1 (CO)	-	-	175.91	175.91
- 2 (CH)	2.56 (hept, *J* = 7.0 Hz)	2.56 (hept, *J* = 7.0 Hz)	33.87	33.87
- 3,4 (CH_3)	1.14 (m)	1.14 (m)	18.85	18.85
**4** (CH)	5.02 (t, *J* = 9.7 Hz)	5.02 (t, *J* = 9.7 Hz)	68.53	68.53
- 1 (CO)	-	-	176.83	176.83
- 2 (CH)	2.49 (hept, *J* = 7.0 Hz)	2.49 (hept, *J* = 7.0 Hz)	33.93	33.93
- 3,4 (CH_3)	1.10 (m)	1.10 (m)	18.87	18.87
**5** (CH)	4.08 (ddd, *J* = 10.3, 4.2, 2.3 Hz)	3.58 (m)	69.47	74.54
**6** (CH_2)	3.57, 3.68 (m)	3.57, 3.68 (m)	61.05	61.05

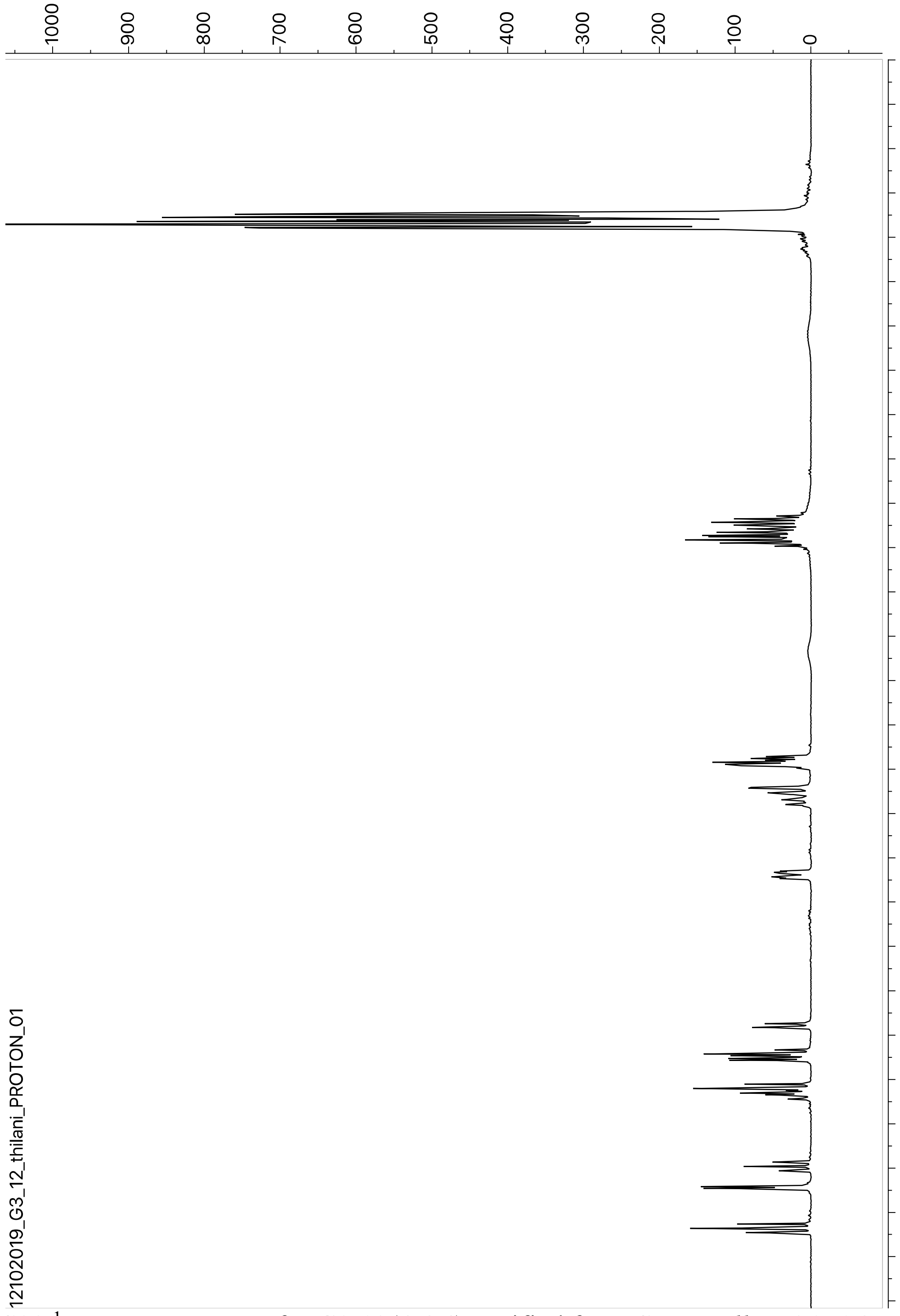

Figure S33 ^{1}H NMR spectrum for G3:12(4,4,4) purified from *S. pennellii* LA0716.

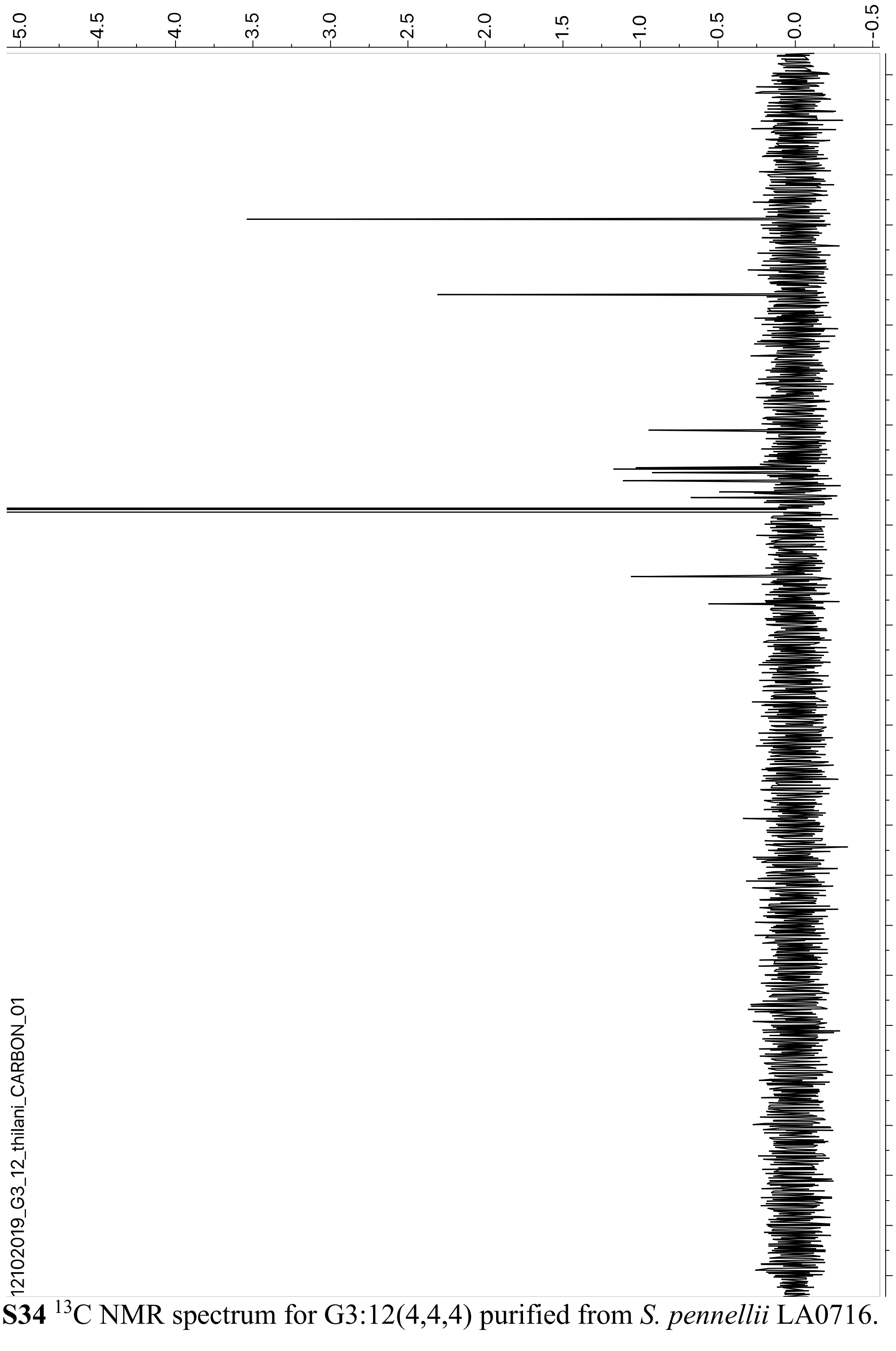

Figure S34 ^{13}C NMR spectrum for G3:12(4,4,4) purified from *S. pennellii* LA0716.

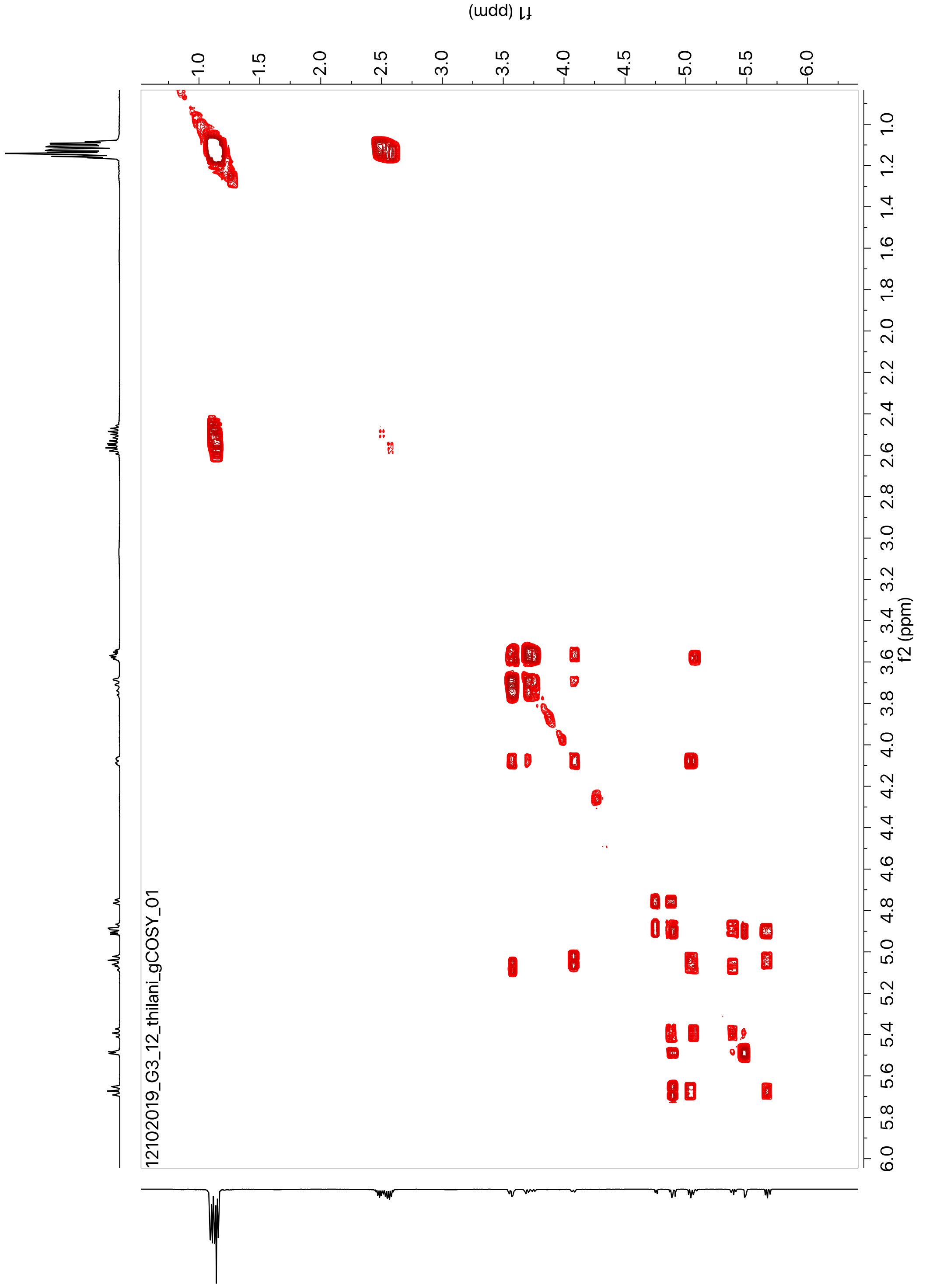

Figure S35 gCOSY NMR spectrum for G3:12(4,4,4) purified from *S. pennellii* LA0716.

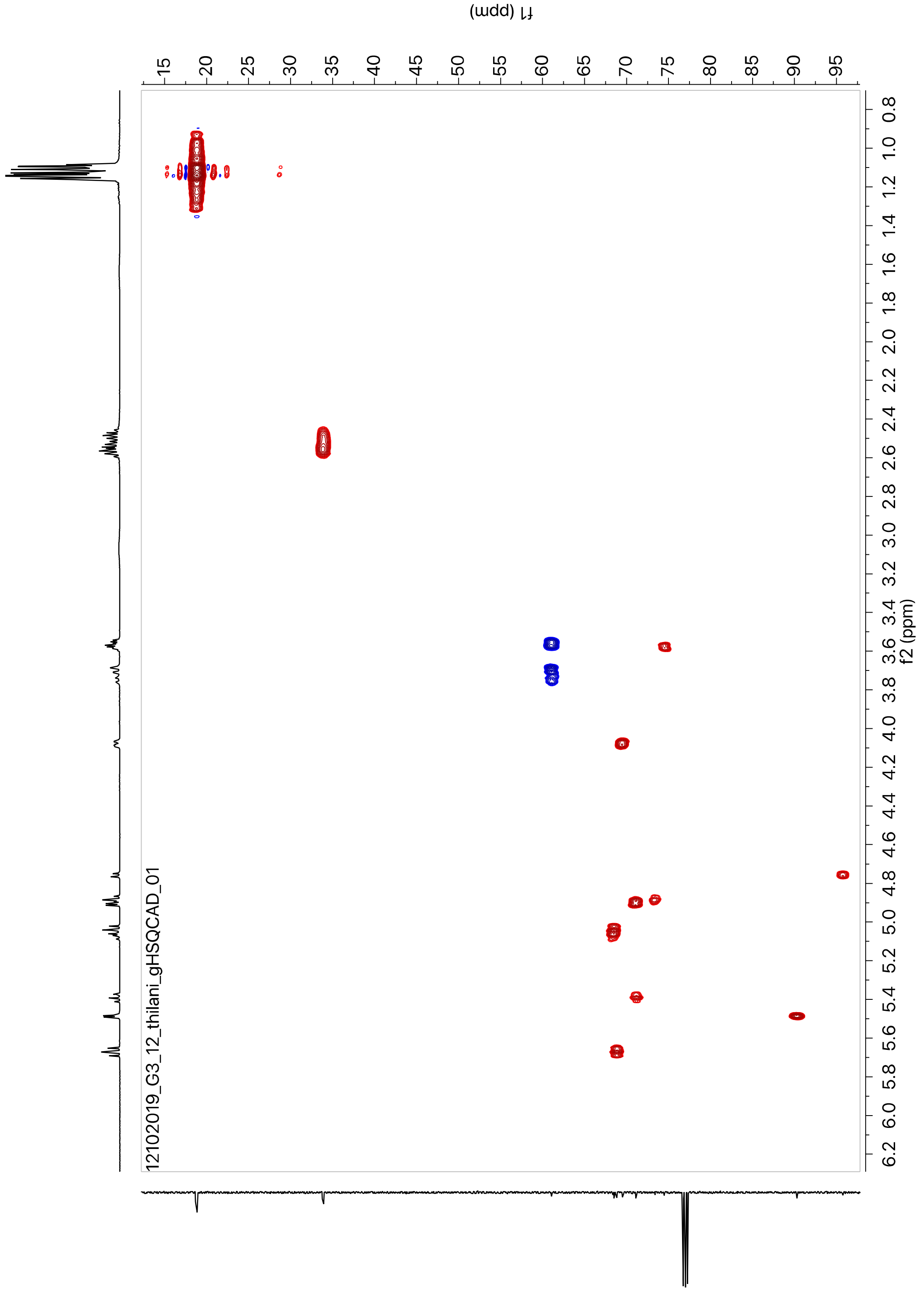

Figure S36 gHSQCAD NMR spectrum for G3:12(4,4,4) purified from *S. pennellii* LA0716.

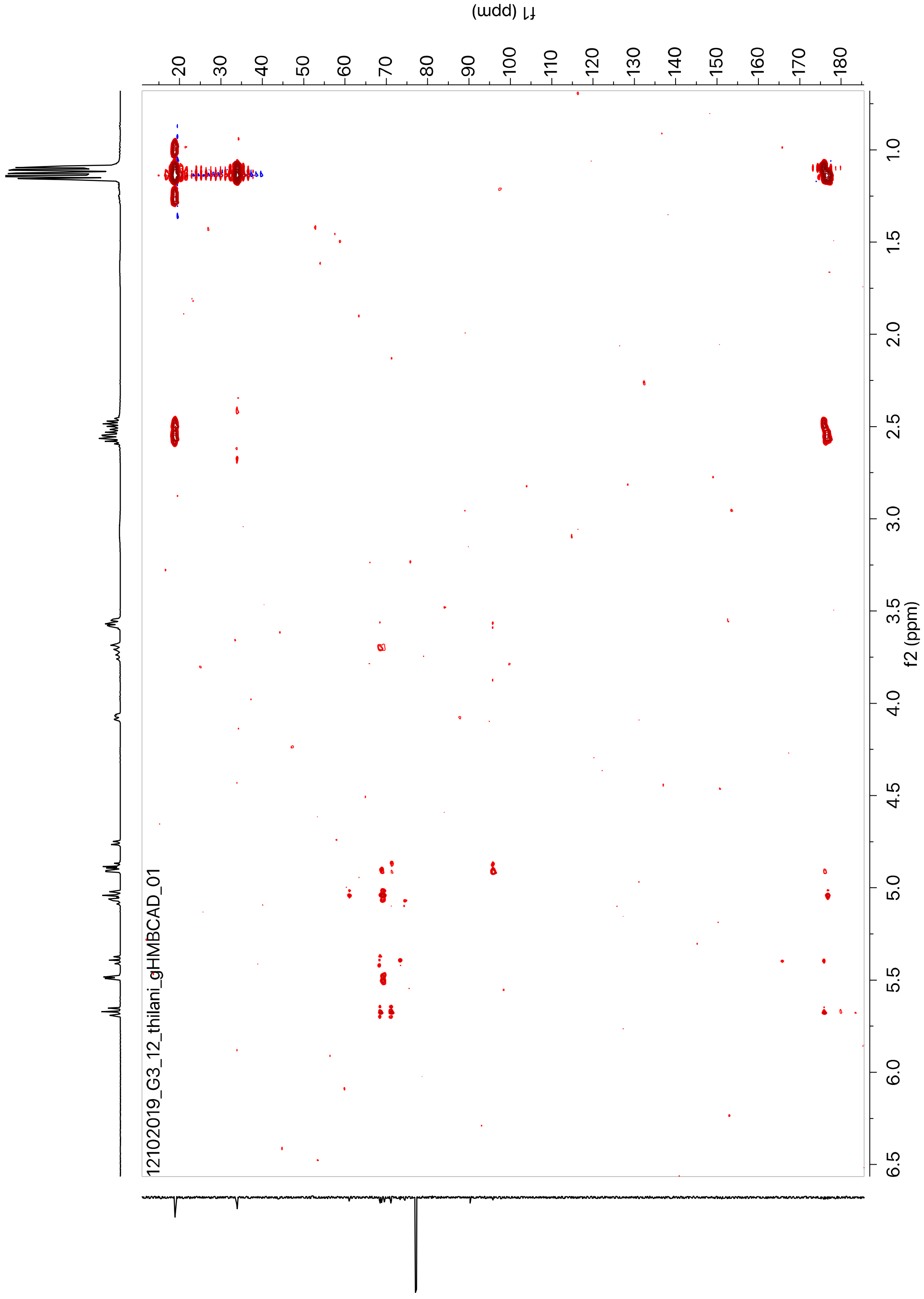

Figure S37 gHMBCAD NMR spectrum for G3:12(4,4,4) purified from *S. pennellii* LA0716.

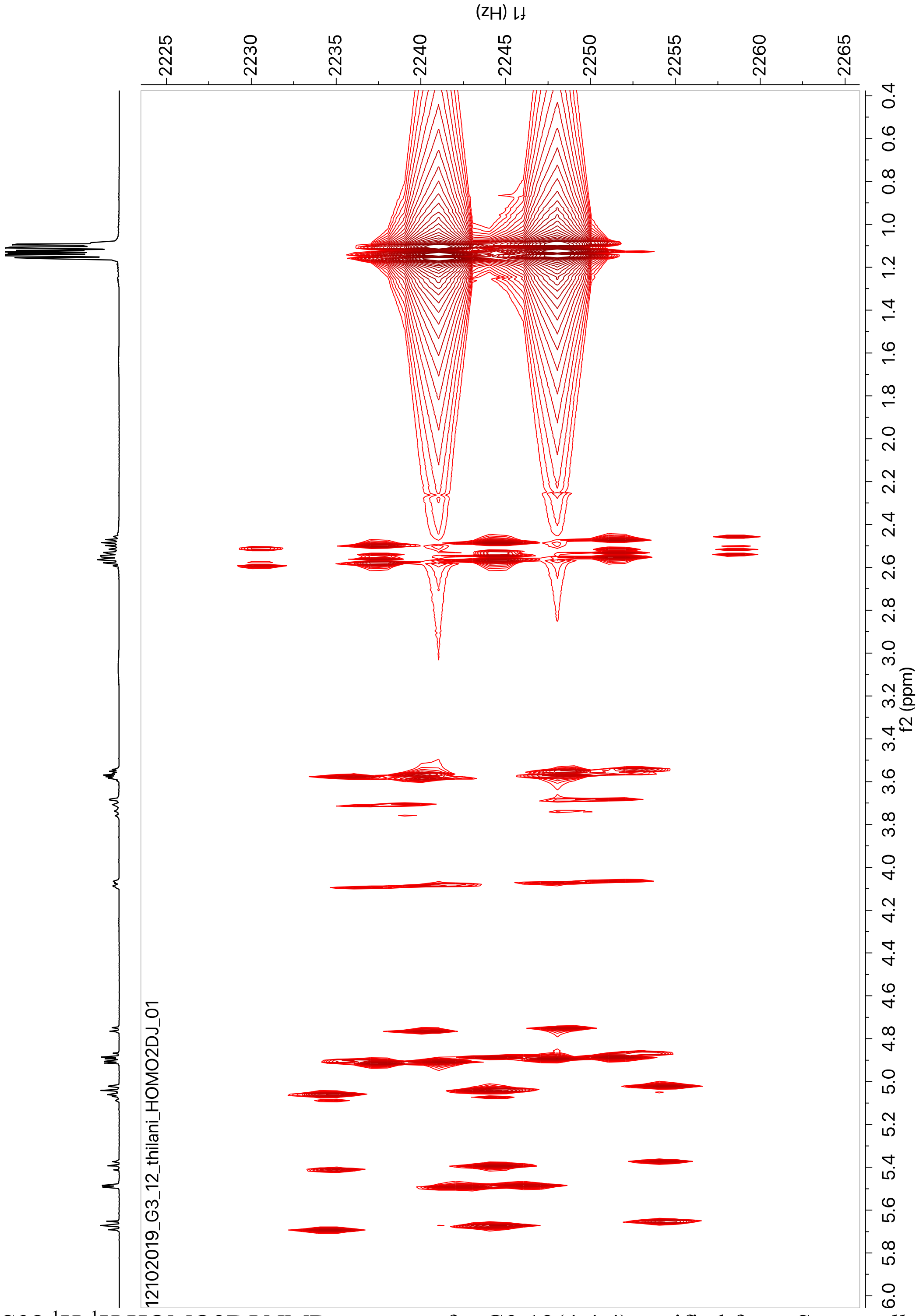

Figure S38 ^{1}H-^{1}H HOMO2DJ NMR spectrum for G3:12(4,4,4) purified from *S. pennellii* LA0716.

Table S12 NMR chemical shifts for G3:18(4,4,10)-1 purified from *S. pennellii* LA0716.

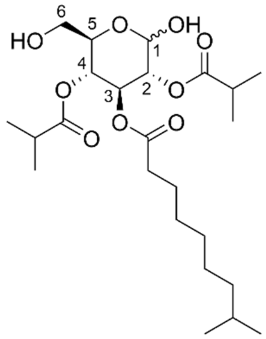

G3:18(4,4,10)-1

Purified from *S. pennellii* LA0716

Chemical Formula: $C_{24}H_{42}O_9$

HRMS: (ESI) *m/z* calculated for $C_{24}H_{42}O_9$ ($[M+NH_4]^+$): 492.3167

Experimental *m/z*: 492.3168

InChI Key: YTHLWGABNVZNEQ-QGZVAWBXSA-N
InChI Key (α): YTHLWGABNVZNEQ-MJALHYBGSA-N
InChI Key (β): YTHLWGABNVZNEQ-UKMCQSRUSA-N

NMR (500 MHz, $CDCl_3$)

Sample mass: 2 mg

**Carbon # (group)**	**^{1}H (ppm)**		**^{13}C (ppm)** (from HSQC and HMBC)	
	α	β	α	β
**1** (CH)	5.50 (d, *J* = 3.7 Hz, 1H)	4.75 (d, *J* = 8.1 Hz, 1H)	90.25	95.78
**2** (CH)	4.88 (dd, *J* = 9.9, 3.7 Hz)	4.85 (m)	71.13	73.44
- 1 (CO)	-	-	176.73	176.73
- 2 (CH)	2.56 (hept, *J* = 7.0 Hz)	2.56 (hept, *J* = 7.0 Hz)	33.92	33.92
- 3,4 (CH_3)	1.14 (m)	1.14 (m)	18.82	18.82
**3** (CH)	5.69 (t, J = 9.9 Hz)	5.41 (t, J = 9.6 Hz)	68.83	71.15
- 1 (CO)	-	-	172.62	172.62
- 2 (CH_2)	2.23 (t, *J* = 7.4 Hz)	2.23 (t, *J* = 7.4 Hz)	34.09	34.09
- 3 (CH_2)	1.54(m)	1.54(m)	24.84	24.84
- 4,5,6 (CH_2)	1.25(m)	1.25(m)	29.37	29.37
- 7 (CH_2)	1.24 (m)	1.24 (m)	27.15	27.15
- 8 (CH)	1.50 (m)	1.50 (m)	27.93	27.93
- 9,10 (CH_3)	0.85 (d, *J* = 6.6 Hz, 6H)	0.85 (d, *J* = 6.6 Hz, 6H)	22.65	22.65
**4** (CH)	5.02 (m)	5.02(m)	68.53	68.53
- 1 (CO)	-	-	176.73	176.73
- 2 (CH)	2.56 (hept, J = 7.0 Hz)	2.56 (hept, J = 7.0 Hz)	33.92	33.92
- 3,4 (CH_3)	1.14 (m)	1.14 (m)	18.82	18.82
**5** (CH)	4.06 (ddd, *J* = 10.2, 4.0, 2.2 Hz)	3.56 (m)	69.53	74.52
**6** (CH_2)	3.53, 3.66 (m)	3.53, 3.66 (m)	61.00	61.00

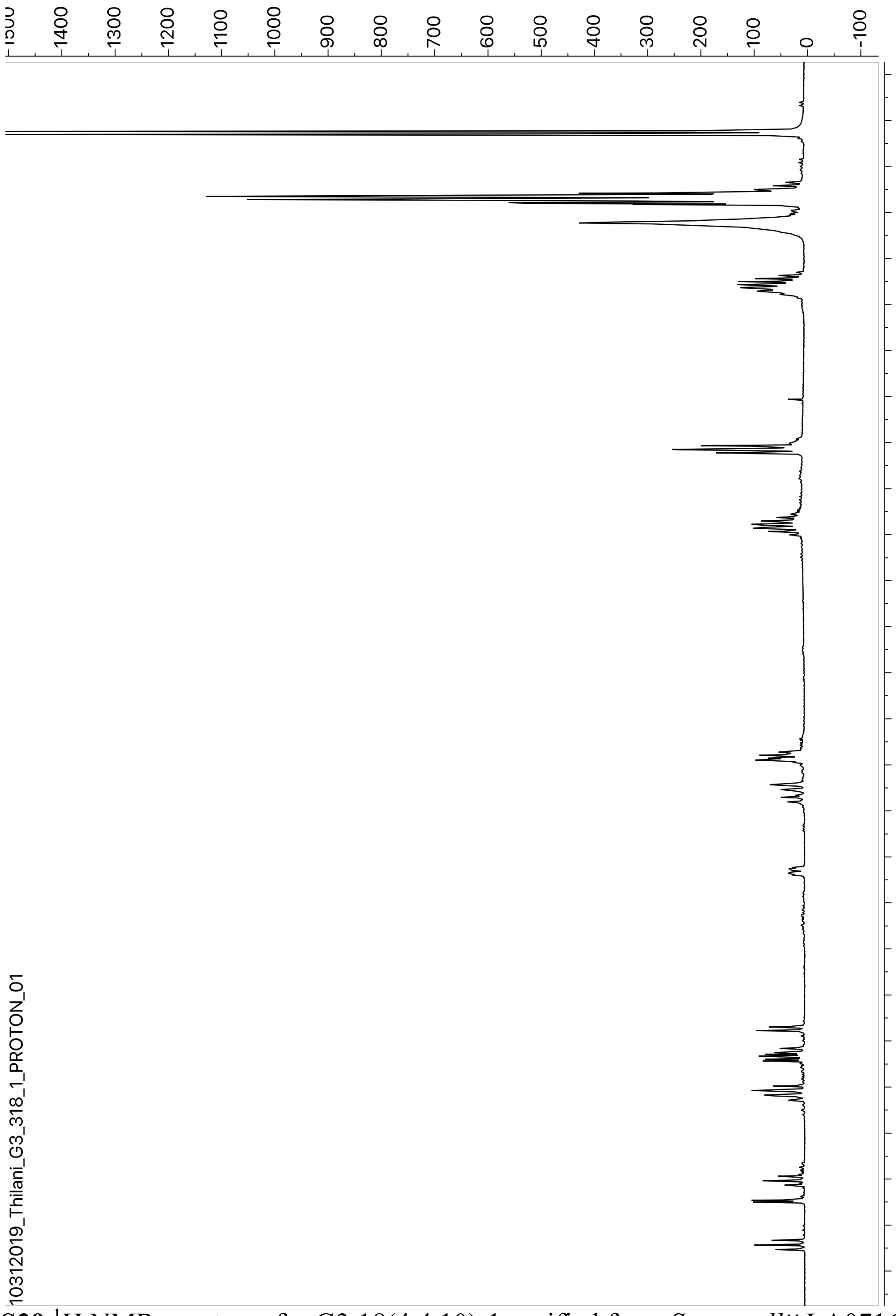

Figure S39 ^{1}H NMR spectrum for G3:18(4,4,10)-1 purified from *S. pennellii* LA0716.

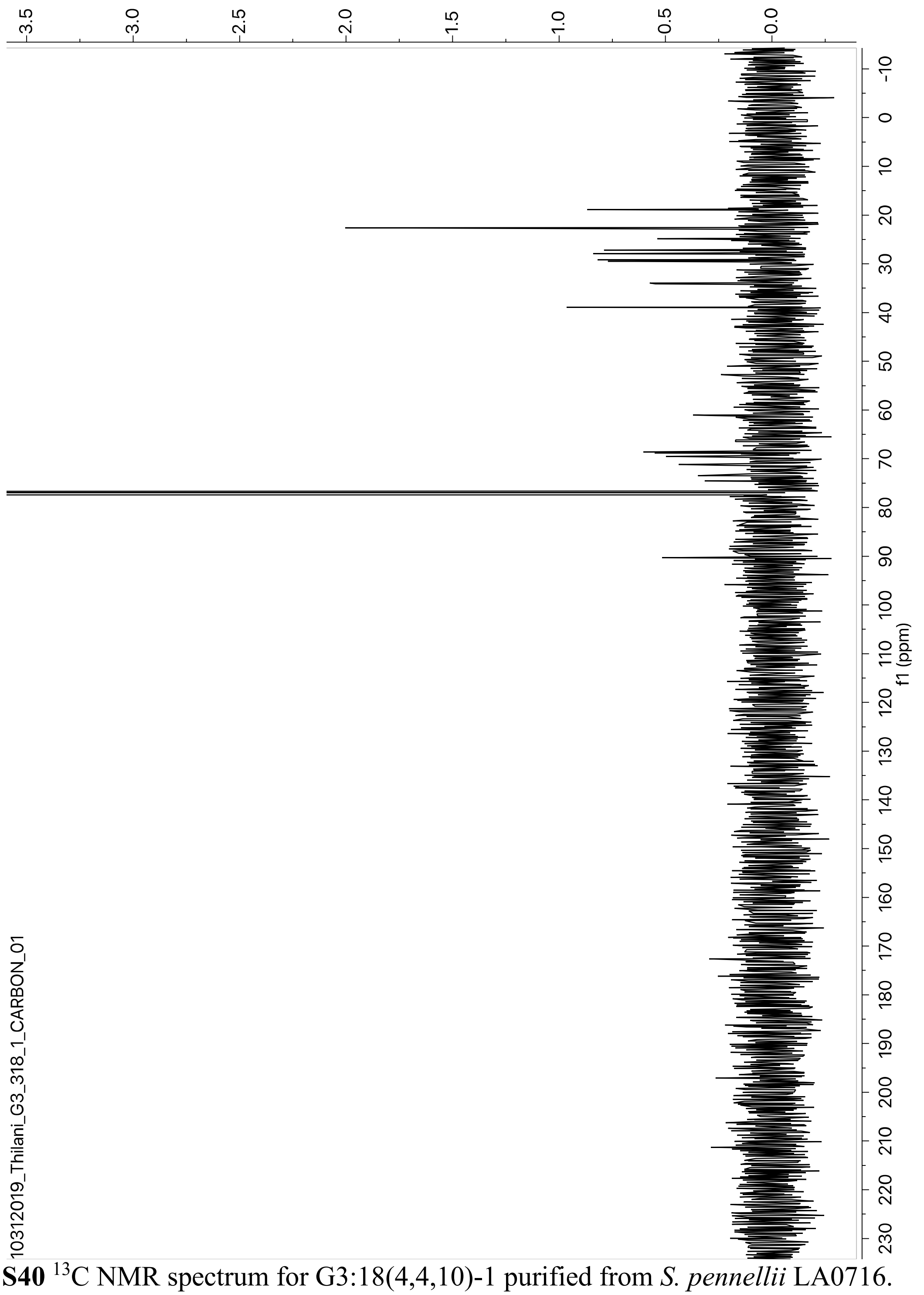

Figure S40 ^{13}C NMR spectrum for G3:18(4,4,10)-1 purified from *S. pennellii* LA0716.

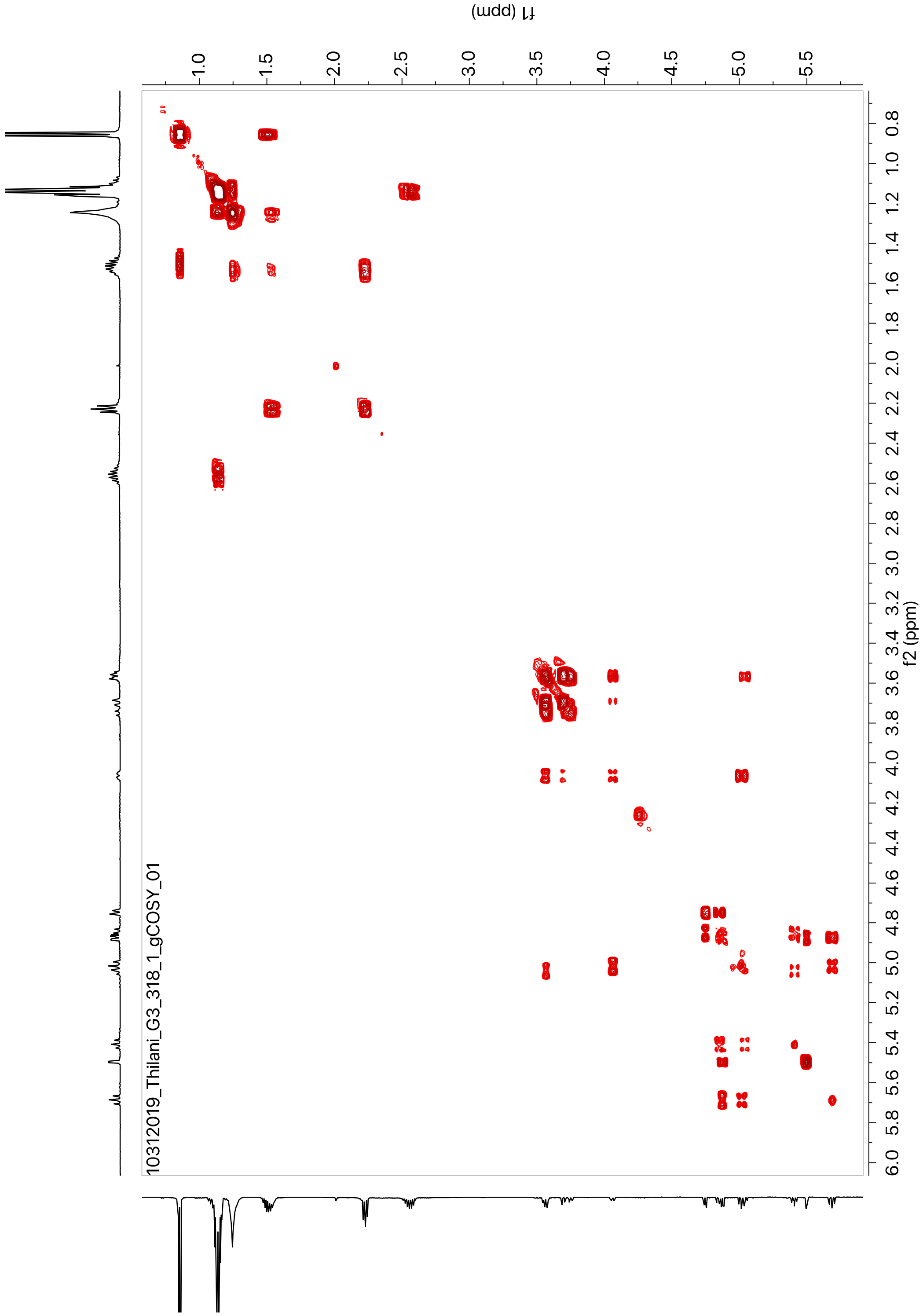

Figure S41 gCOSY NMR spectrum for G3:18(4,4,10)-1 purified from *S. pennellii* LA0716.

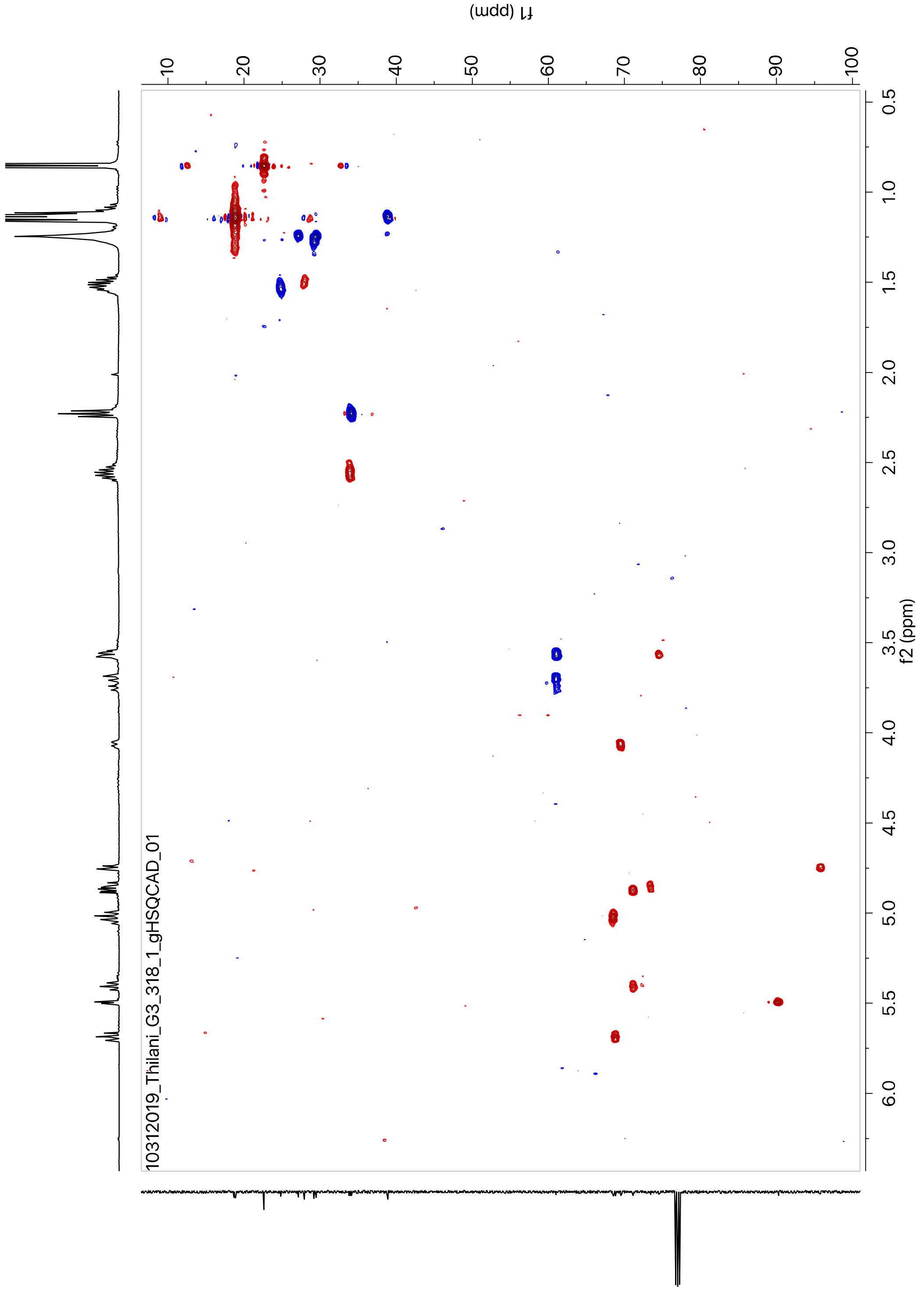

Figure S42 gHSQCAD NMR spectrum for G3:18(4,4,10)-1 purified from *S. pennellii* LA0716.

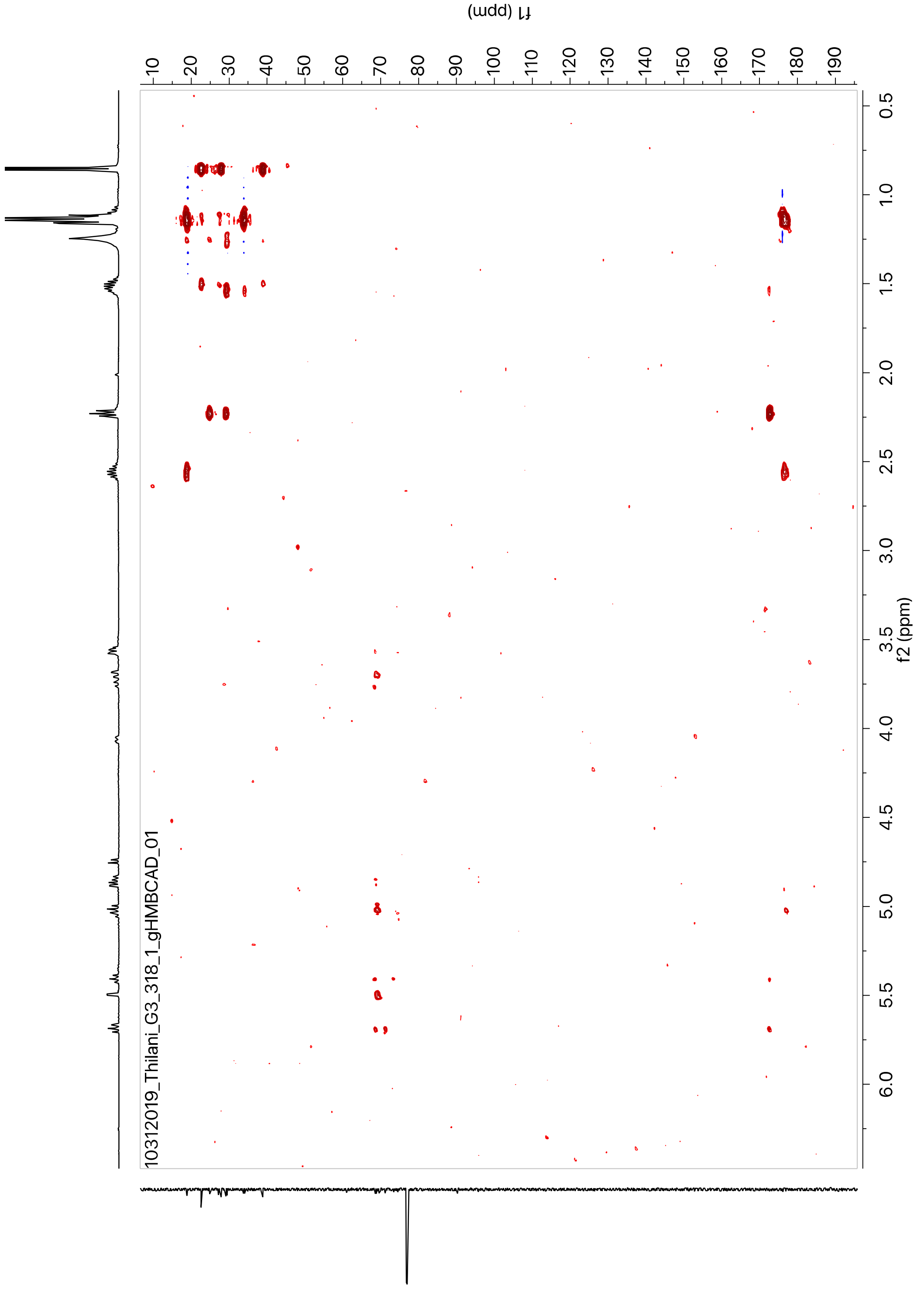

Figure S43 gHMBCAD NMR spectrum for G3:18(4,4,10)-1 purified from *S. pennellii* LA0716.

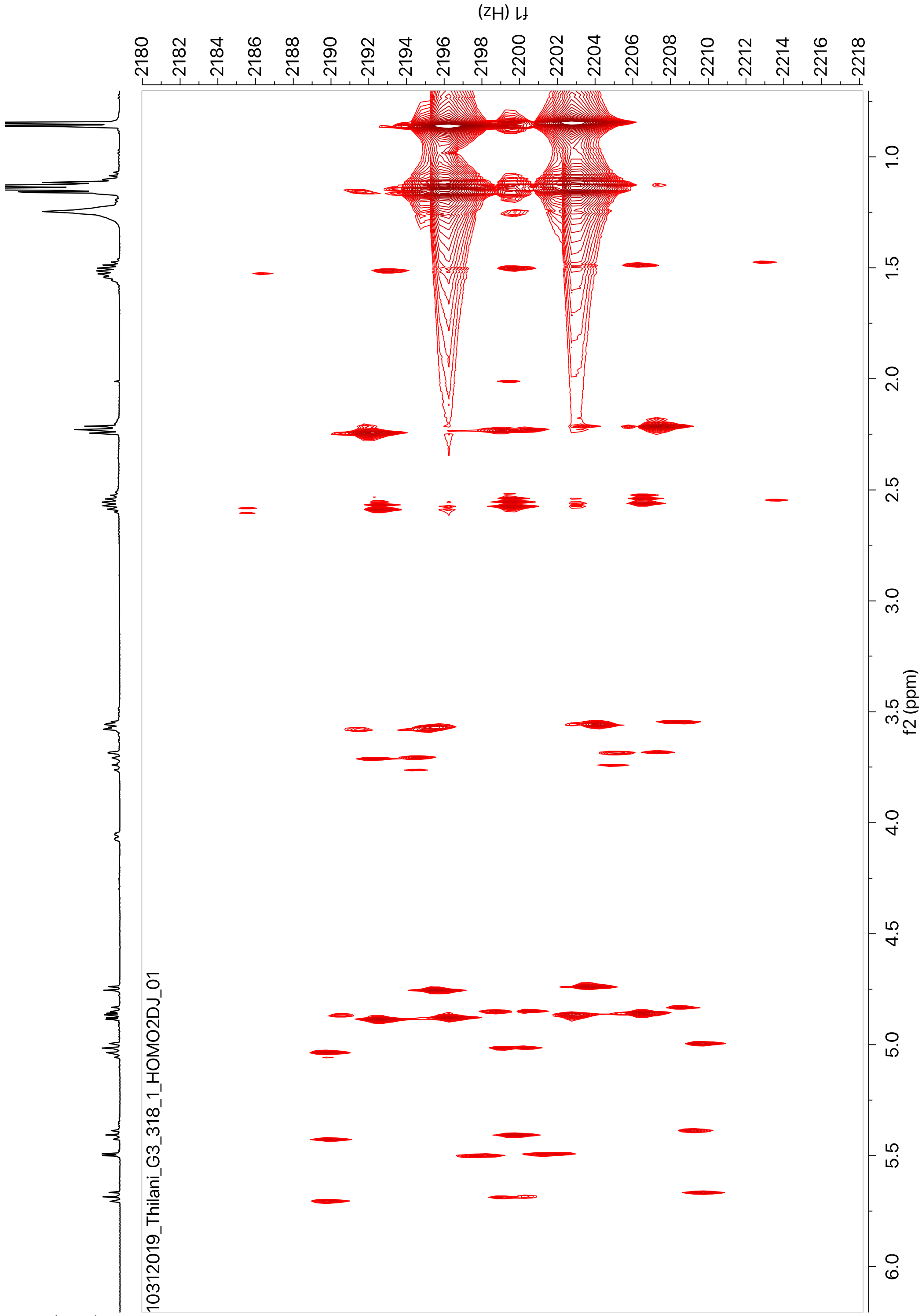

Figure S44 ^{1}H-^{1}H HOMO2DJ NMR spectrum for G3:18(4,4,10)-1 purified from *S. pennellii* LA0716.

Table S13 NMR chemical shifts for G3:18(4,4,10)-2 purified from *S. pennellii* LA0716.

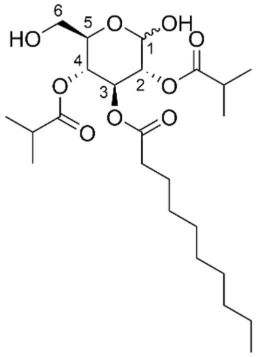

G3:18(4,4,10)-2

Purified from *S. pennellii* LA0716

Chemical Formula: $C_{24}H_{42}O_9$

HRMS: (ESI) *m/z* calculated for $C_{24}H_{42}O_9$ ($[M+NH_4]^+$): 492.3167

Experimental *m/z*: 492.3170

InChI Key: LJSYEIZIEREFSJ-QGZVAWBXSA-N
InChI Key (a): LJSYEIZIEREFSJ-MJALHYBGSA-N
InChI Key (b): LJSYEIZIEREFSJ-UKMCQSRUSA-N

NMR (500 MHz, $CDCl_3$)

Sample mass: 2 mg

**Carbon # (group)**	**^{1}H (ppm)**		**^{13}C (ppm)** (from HSQC and HMBC)	
	α	β	α	β
**1** (CH)	5.50 (d, *J* = 3.4 Hz)	4.75 (d, *J* = 8.1 Hz)	90.29	95.77
**2** (CH)	4.86 (dd, *J* = 10.0, 3.4 Hz)	4.85 (m)	71.15	73.48
- 1 (CO)	-	-	176.83	176.83
- 2 (CH)	2.56 (hept, *J* = 7.0 Hz)	2.56 (hept, *J* = 7.0 Hz)	33.89	33.89
- 3,4 (CH_3)	1.15 (m)	1.15 (m)	18.79	18.79
**3** (CH)	5.69 (t, J = 10.0 Hz)	5.40 (t, J = 9.7 Hz)	68.83	71.13
- 1 (CO)	-	-	172.82	172.82
- 2 (CH_2)	2.23 (t, *J* = 7.4 Hz)	2.23 (t, *J* = 7.4 Hz)	34.10	34.10
- 3 (CH_2)	1.53(m)	1.53(m)	24.82	24.82
- 4,5,6,7 (CH_2)	1.24(m)	1.24(m)	29.20	29.20
- 8 (CH_2)	1.24 (m)	1.24 (m)	31.79	31.79
- 9 (CH_2)	1.28 (m)	1.28 (m)	22.70	22.70
- 10 (CH_3)	0.88 (t, *J* = 7.0 Hz)	0.88 (t, *J* = 7.0 Hz)	14.06	14.06
**4** (CH)	5.02 (m)	5.02(m)	68.54	68.54
- 1 (CO)	-	-	176.83	176.83
- 2 (CH)	2.56 (hept, J = 7.0 Hz)	2.56 (hept, J = 7.0 Hz)	33.89	33.89
- 3,4 (CH_3)	1.15 (m)	1.15 (m)	18.79	18.79
**5** (CH)	4.07 (ddd, *J* = 10.2, 4.0, 2.3 Hz)	3.56 (m)	69.57	74.53
**6** (CH_2)	3.57, 3.68 (m)	3.57, 3.68 (m)	61.06	61.06

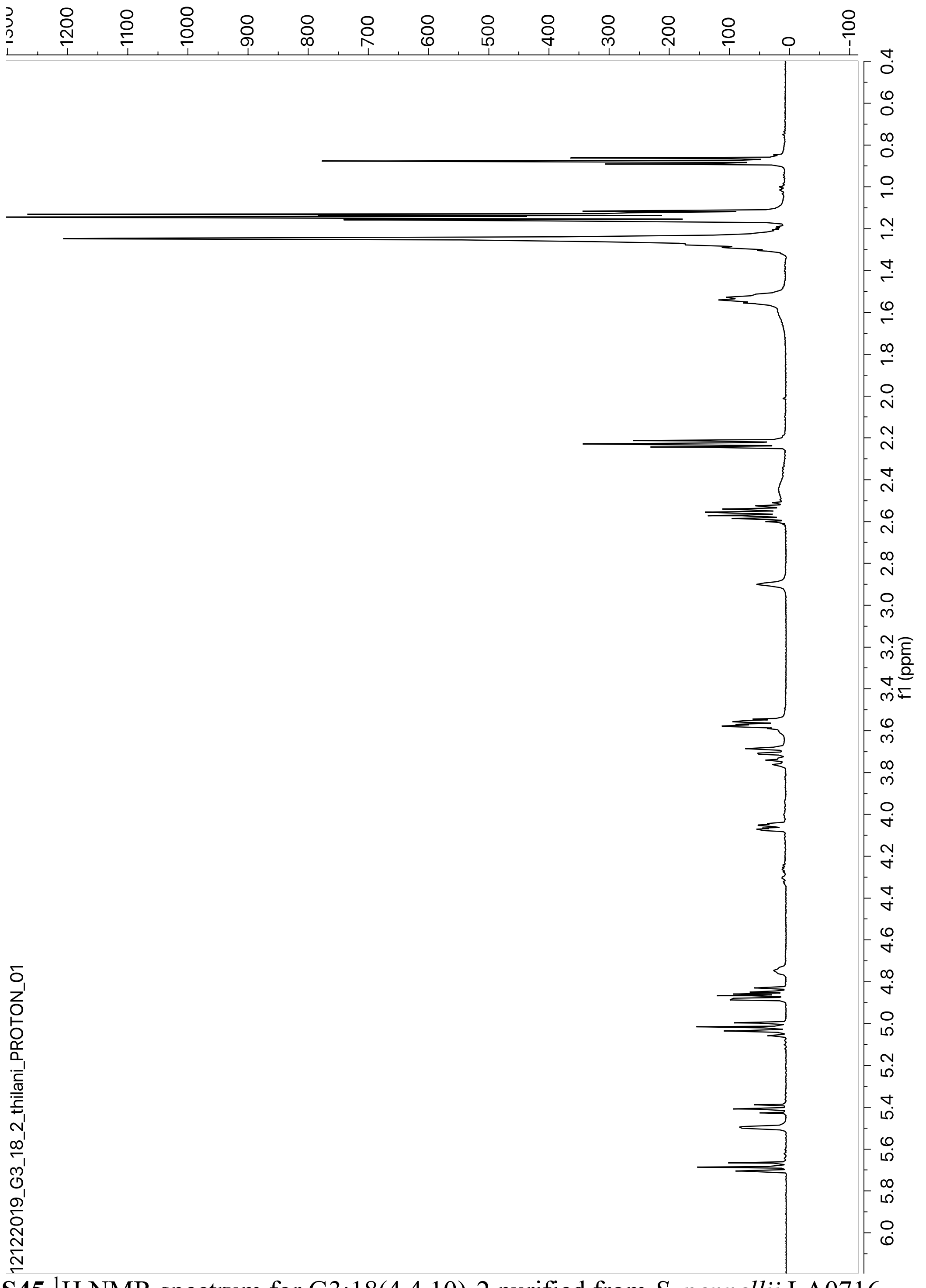

Figure S45 ^{1}H NMR spectrum for G3:18(4,4,10)-2 purified from *S. pennellii* LA0716.

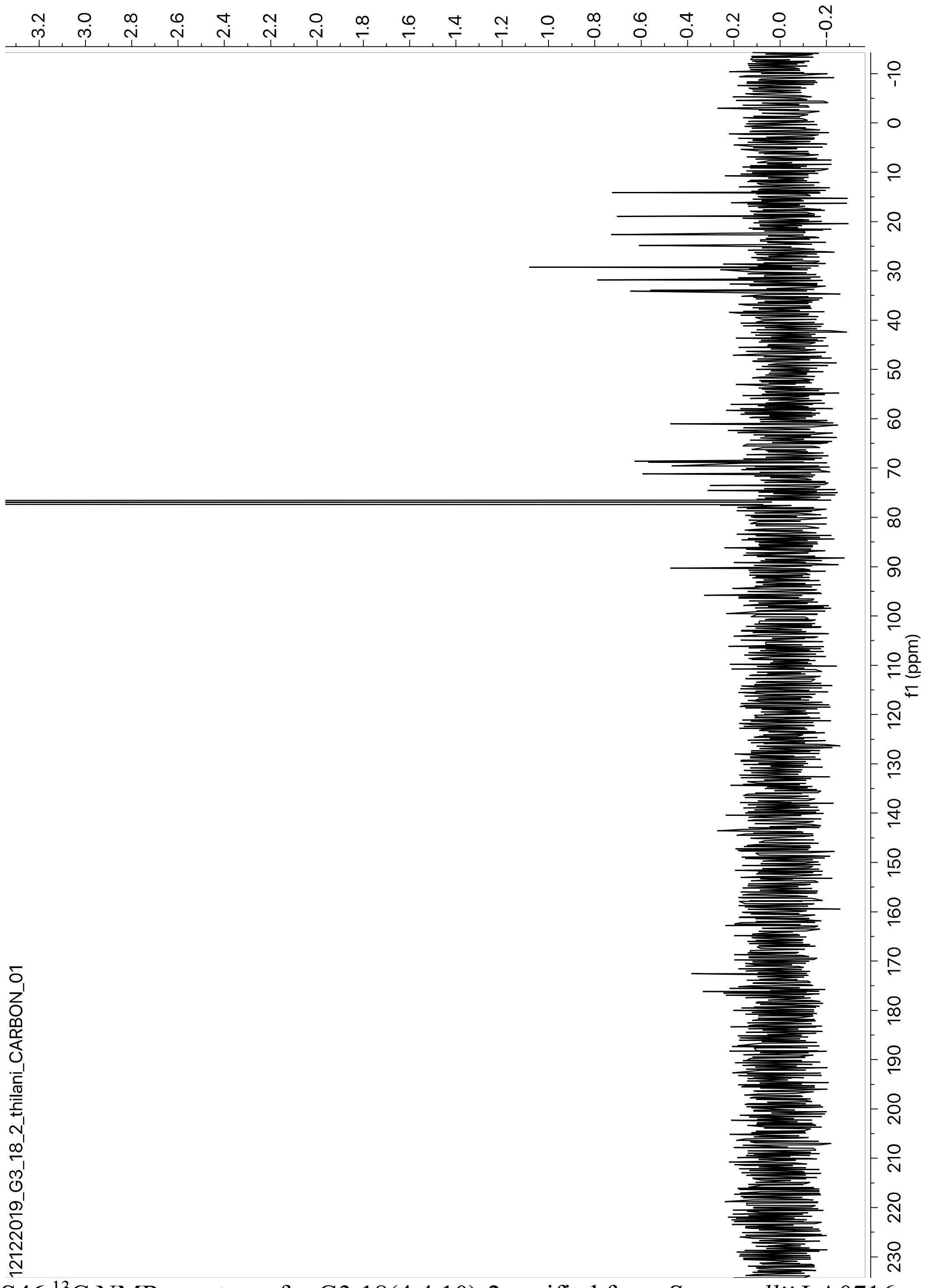

Figure S46 ^{13}C NMR spectrum for G3:18(4,4,10)-2 purified from *S. pennellii* LA0716.

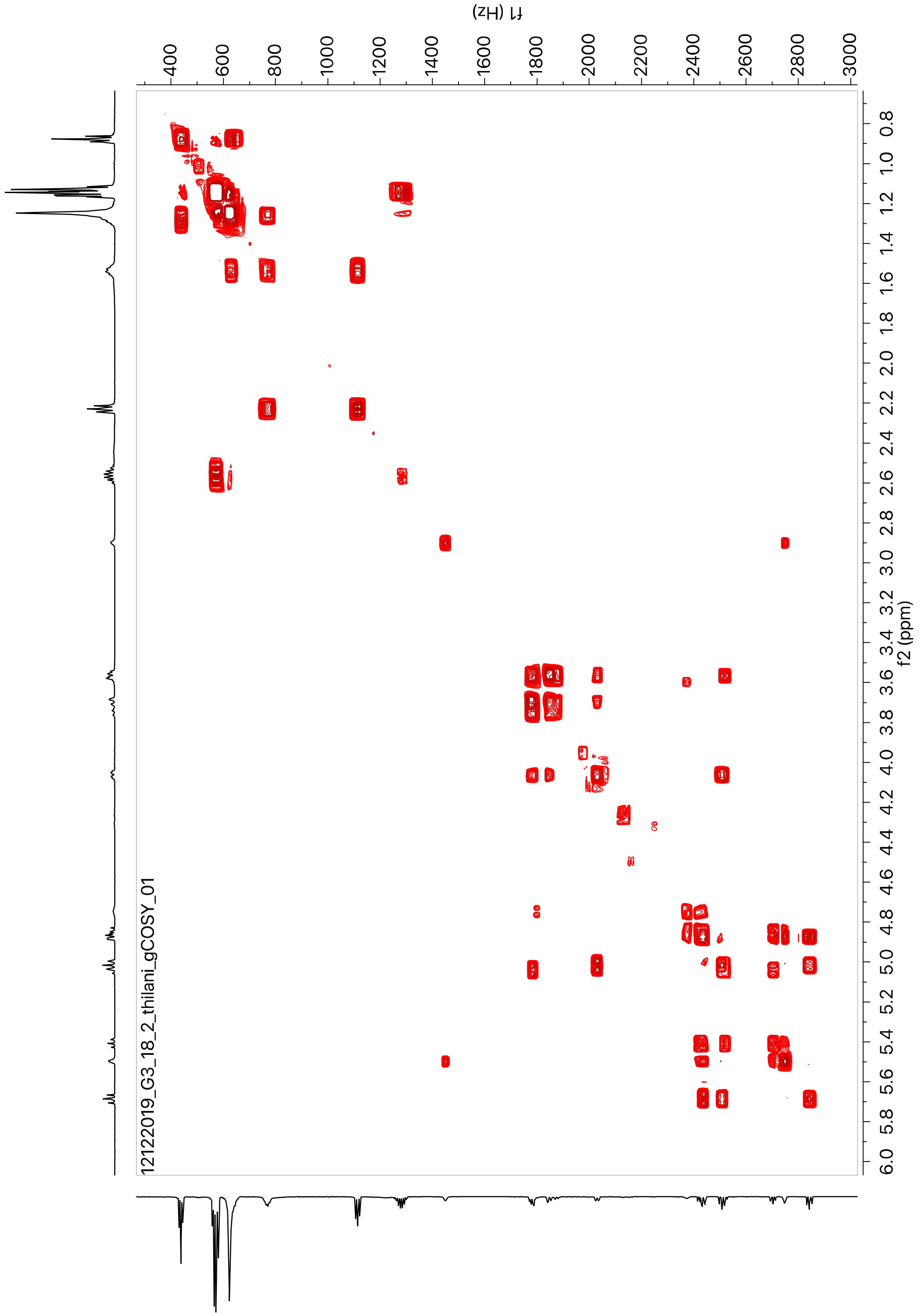

Figure S47 gCOSY NMR spectrum for G3:18(4,4,10)-2 purified from *S. pennellii* LA0716.

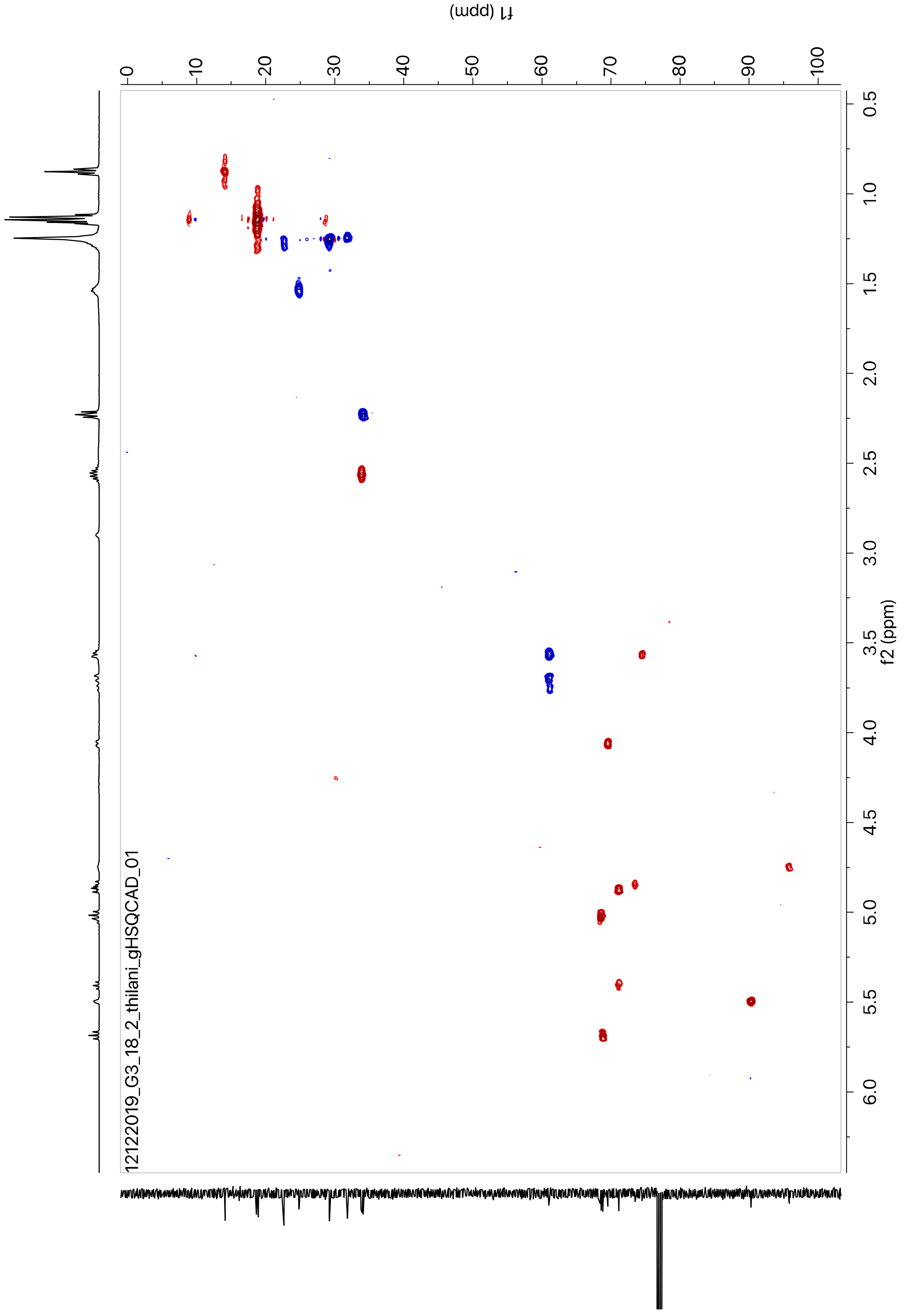

Figure S48 gHSQCAD NMR spectrum for G3:18(4,4,10)-2 purified from *S. pennellii* LA0716.

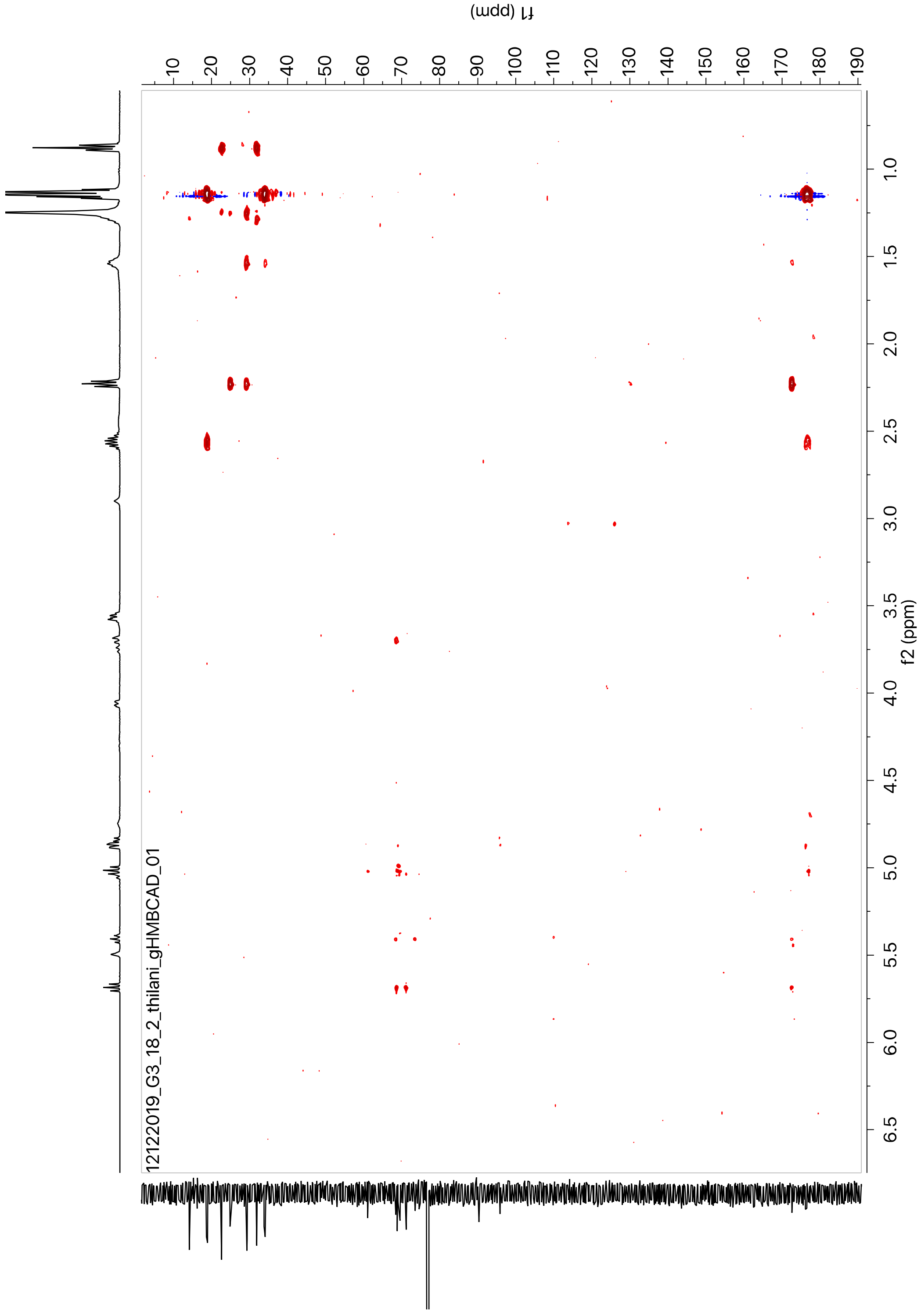

Figure S49 gHMBCAD NMR spectrum for G3:18(4,4,10)-2 purified from *S. pennellii* LA0716.

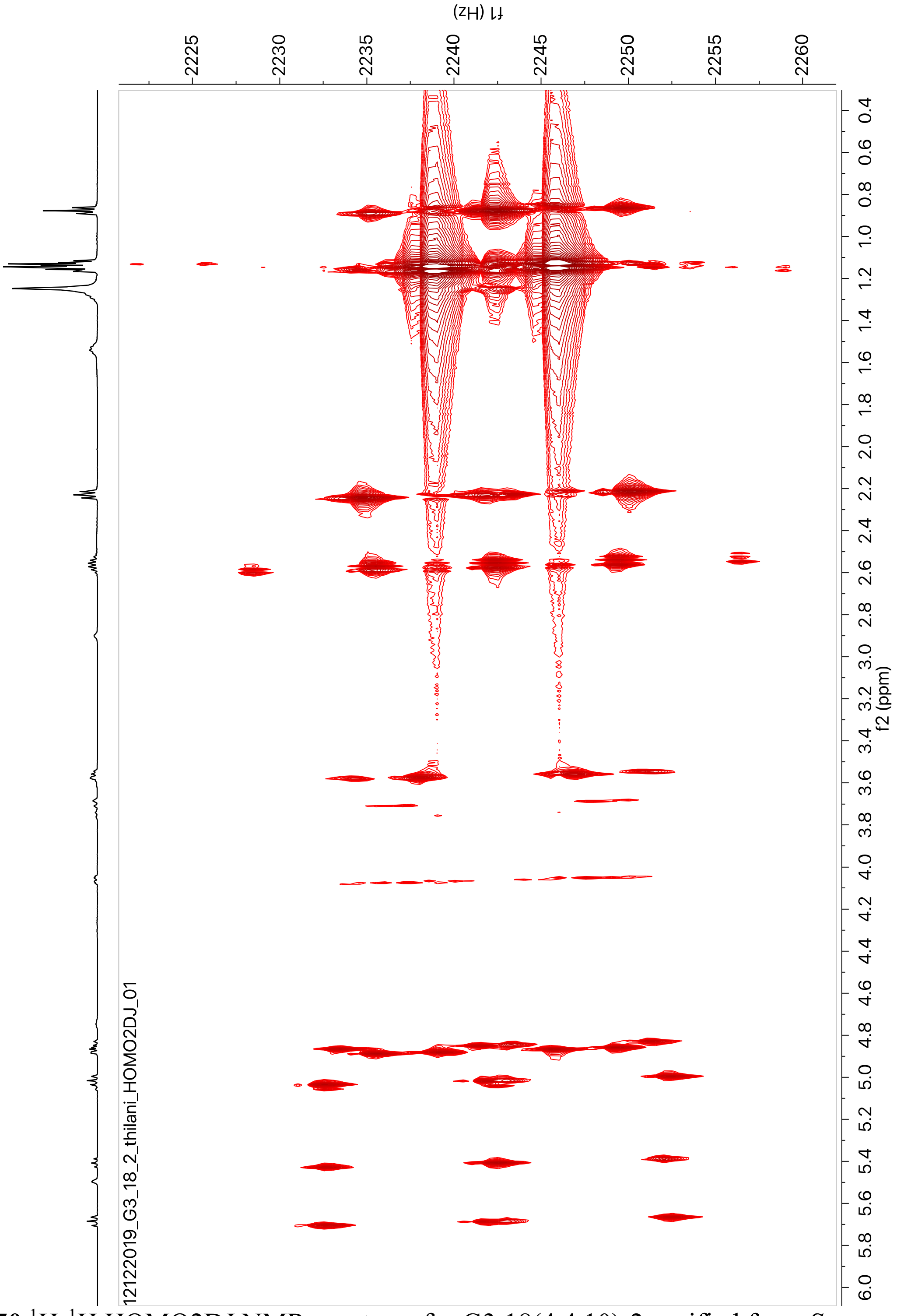

Figure S50 ^{1}H-^{1}H HOMO2DJ NMR spectrum for G3:18(4,4,10)-2 purified from *S. pennellii* LA0716.

Table S14 NMR chemical shifts for G3:19(4,5,10)-1 purified from *S. pennellii* LA0716.

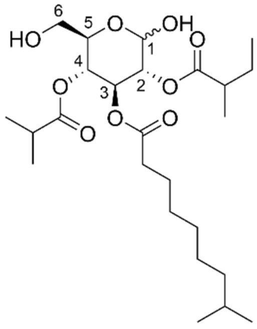

G3:19(4,5,10)-1

Purified from *S. pennellii* LA0716

Chemical Formula: $C_{24}H_{42}O_9$

HRMS: (ESI) *m/z* calculated for $C_{24}H_{42}O_9$ ($[M+NH_4]^+$): 506.3324

Experimental *m/z*: 506.3328

InChI Key: UCMUJVLLMOVWPV-VSLCRTCVSA-N
InChI Key (α): UCMUJVLLMOVWPV-ONCJQFAMSA-N
InChI Key (β): UCMUJVLLMOVWPV-MBFSEYTRSA-N

NMR (500 MHz, $CDCl_3$)

Sample mass: 2 mg

**Carbon # (group)**	**^{1}H (ppm)**		**^{13}C (ppm)** (from HSQC and HMBC)	
	α	β	α	β
**1** (CH)	5.51 (d, *J* = 3.6 Hz)	4.74 (d, *J* = 8.1 Hz)	90.29	95.86
**2** (CH)	4.88 (m)	4.87 (m)	71.17	73.35
- 1 (CO)	-	-	176.22	176.22
- 2 (CH)	2.41 (sextet, *J* = 6.9 Hz)	2.41 (sextet, *J* = 6.9 Hz)	40.92	40.92
- 3 (CH_3)	1.13 (m, 3H)	1.13 (m, 3H)	16.35	16.35
- 4 (CH_2)	1.45, 1.65 (m, 2H)	1.45, 1.65 (m, 2H)	26.49	26.49
- 5 (CH_3)	0.88 (t, *J* = 7.3 Hz, 3H)	0.88 (t, *J* = 7.3 Hz, 3H)	11.50	11.50
**3** (CH)	5.69 (t, J = 9.9 Hz)	5.41 (t, J = 9.6 Hz)	68.69	71.09
- 1 (CO)	-	-	172.71	172.71
- 2 (CH_2)	2.23 (t, *J* = 7.4 Hz)	2.23 (t, *J* = 7.4 Hz)	34.05	34.05
- 3 (CH_2)	1.54(m)	1.54(m)	24.76	24.76
- 4,5,6 (CH_2)	1.25(m)	1.25(m)	29.27	29.27
- 7 (CH_2)	1.24 (m)	1.24 (m)	27.22	27.22
- 8 (CH)	1.50 (m)	1.50 (m)	27.88	27.88
- 9,10 (CH_3)	0.88 (d, *J* = 7.0 Hz, 6H)	0.88 (d, *J* = 7.0 Hz, 6H)	22.48	22.48
**4** (CH)	5.04 (m)	5.04(m)	68.55	68.55
- 1 (CO)	-	-	176.73	176.73
- 2 (CH)	2.54 (hept, J = 7.0 Hz)	2.54 (hept, J = 7.0 Hz)	33.98	33.98
- 3,4 (CH_3)	1.13 (m)	1.13 (m)	18.31	18.31
**5** (CH)	4.07 (ddd, *J* = 10.2, 4.0, 2.2 Hz)	3.58 (m)	69.49	74.58
**6** (CH_2)	3.58, 3.69 (m)	3.58, 3.69 (m)	61.10	61.10

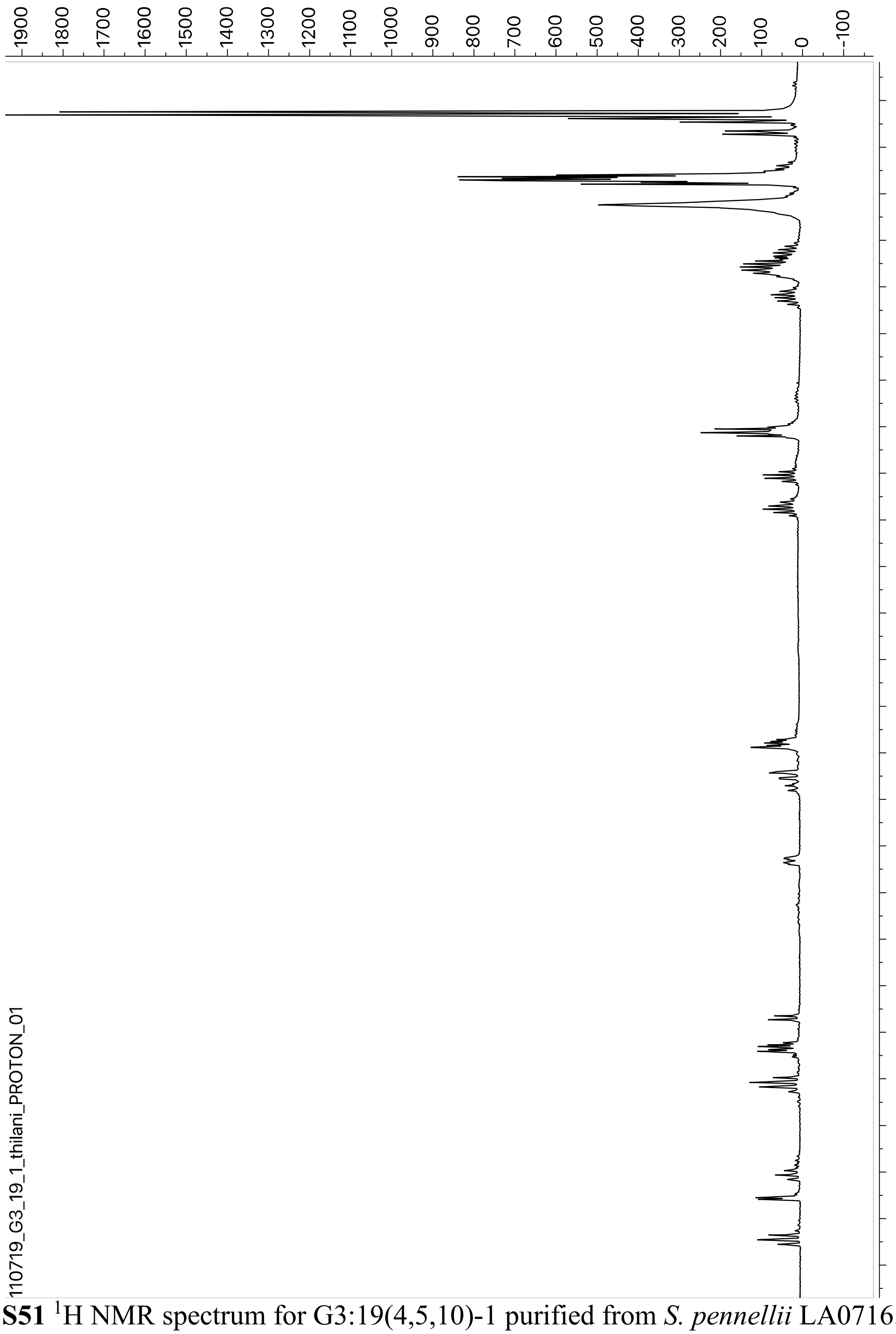

Figure S51 ^{1}H NMR spectrum for G3:19(4,5,10)-1 purified from *S. pennellii* LA0716.

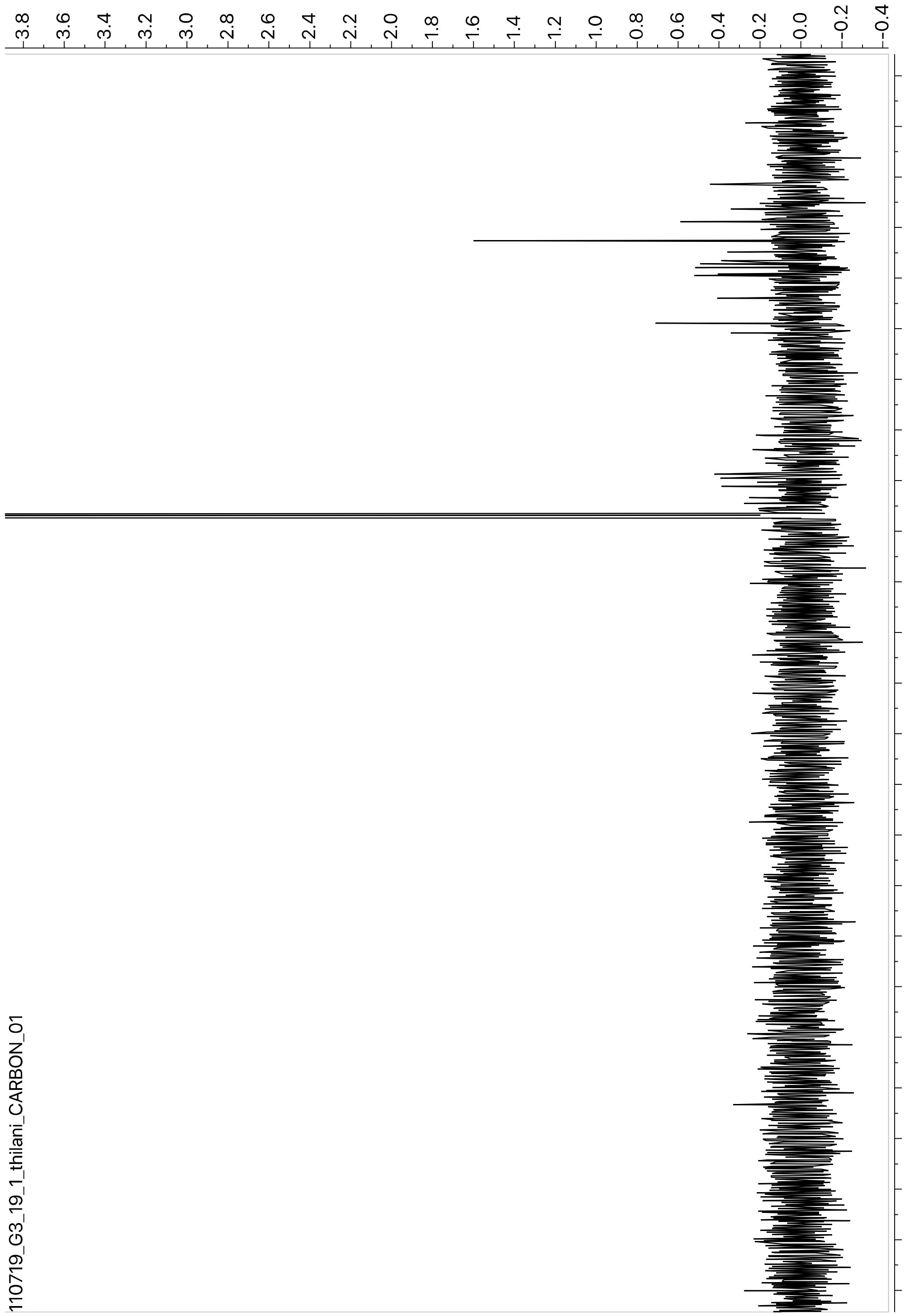

Figure S52 ^{13}C NMR spectrum for G3:19(4,5,10)-1 purified from *S. pennellii* LA0716.

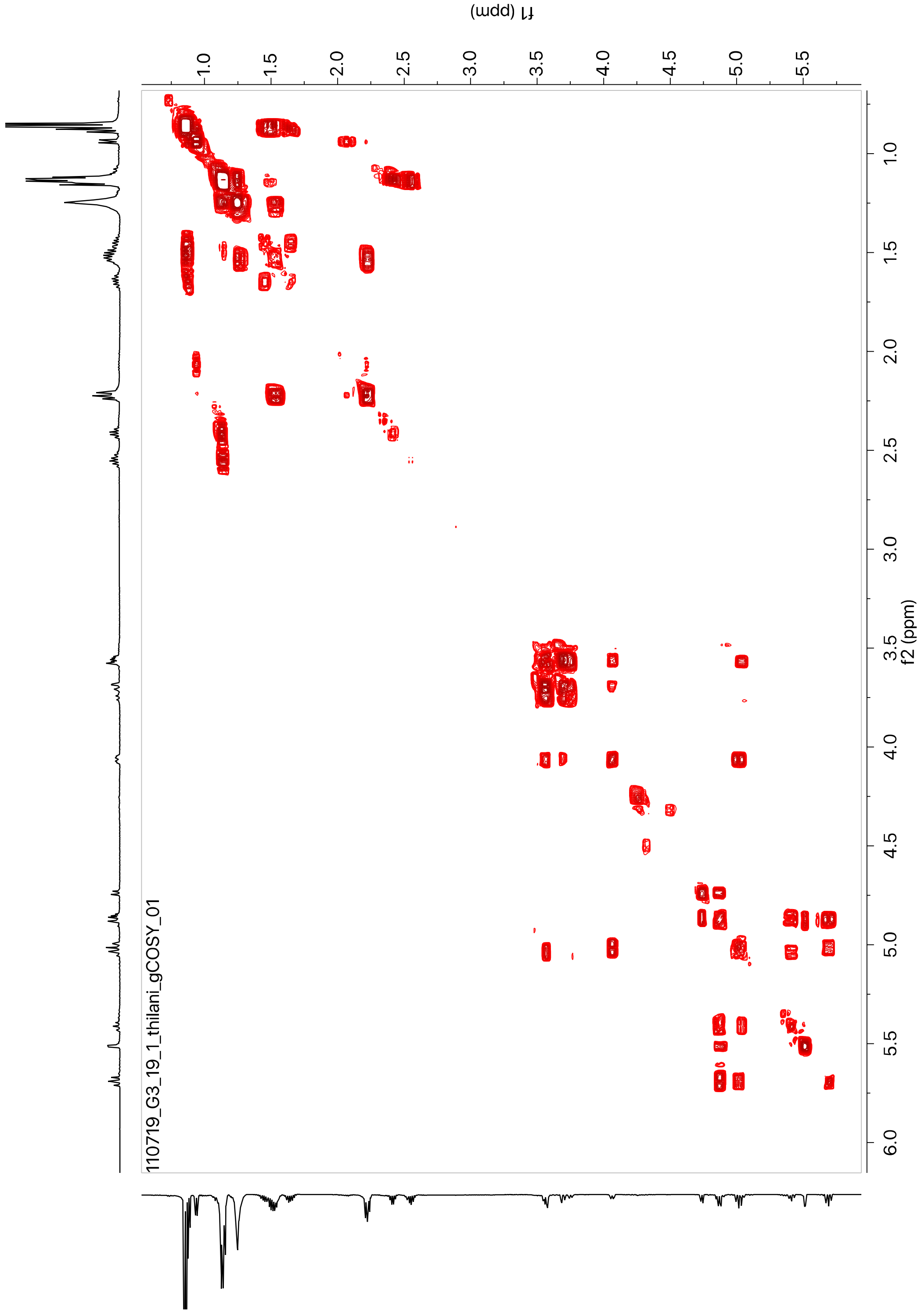

Figure S53 gCOSY NMR spectrum for G3:19(4,5,10)-1 purified from *S. pennellii* LA0716.

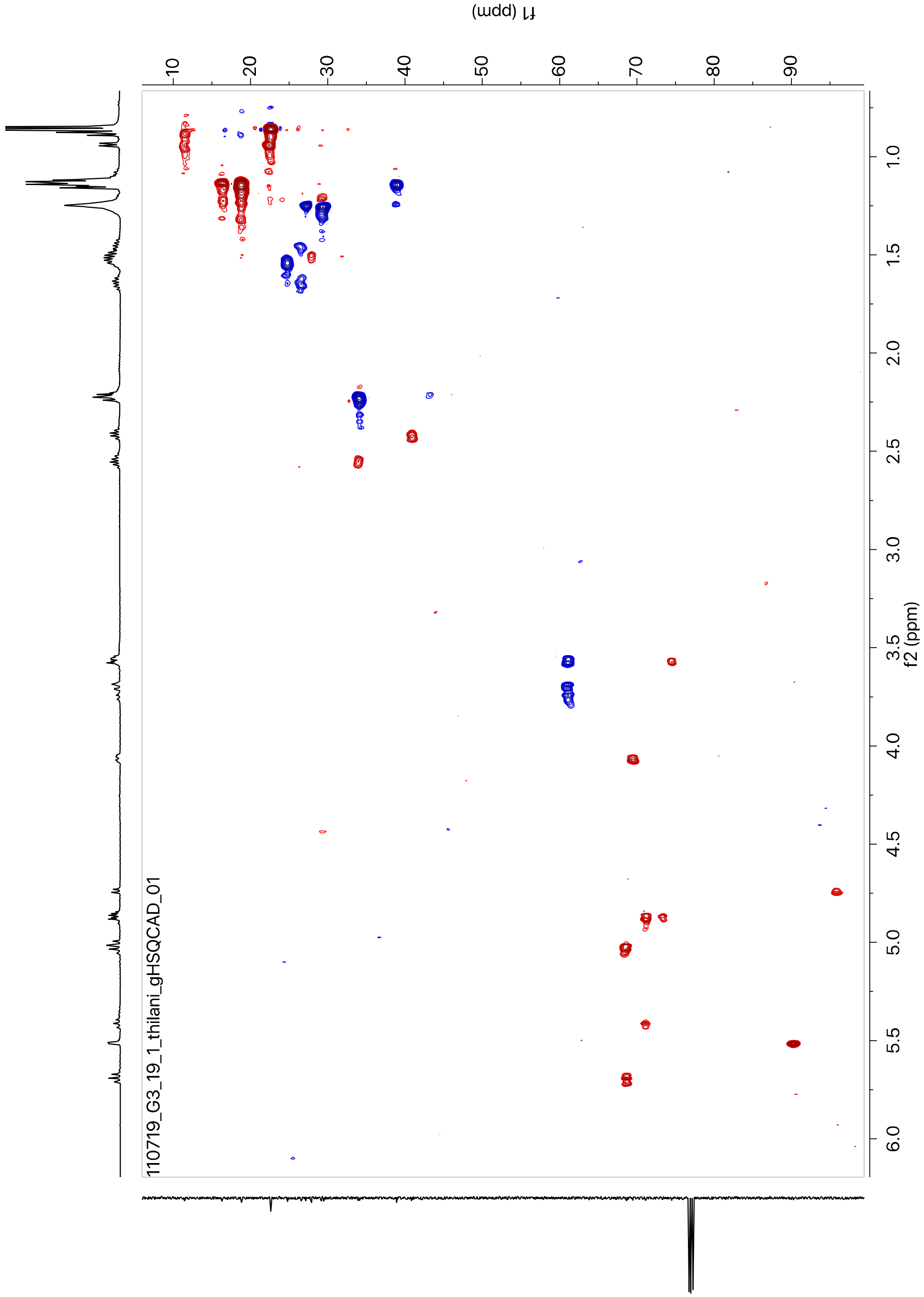

Figure S54 gHSQCAD NMR spectrum for G3:19(4,5,10)-1 purified from *S. pennellii* LA0716.

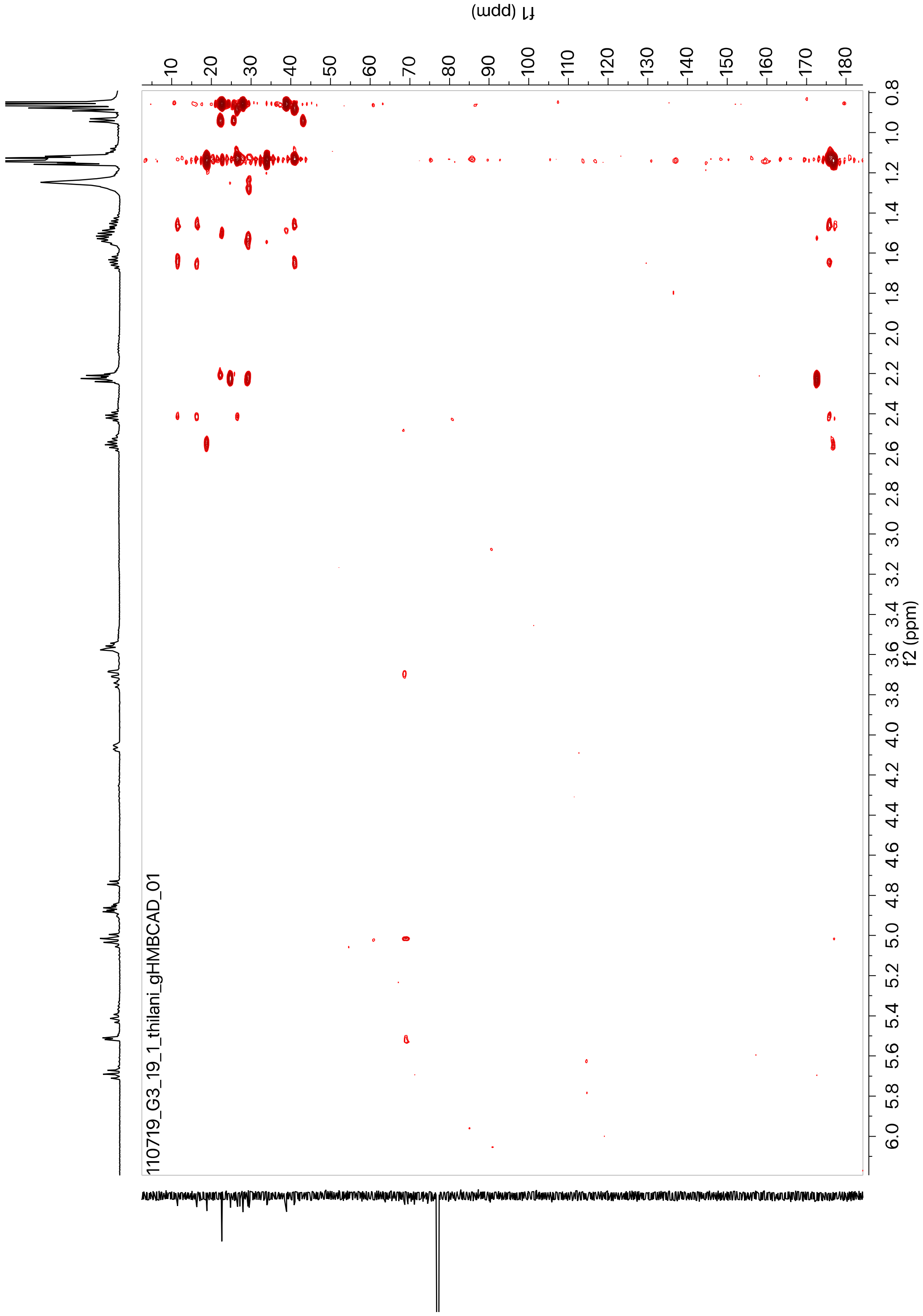

Figure S55 gHMBCAD NMR spectrum for G3:19(4,5,10)-1 purified from *S. pennellii* LA0716.

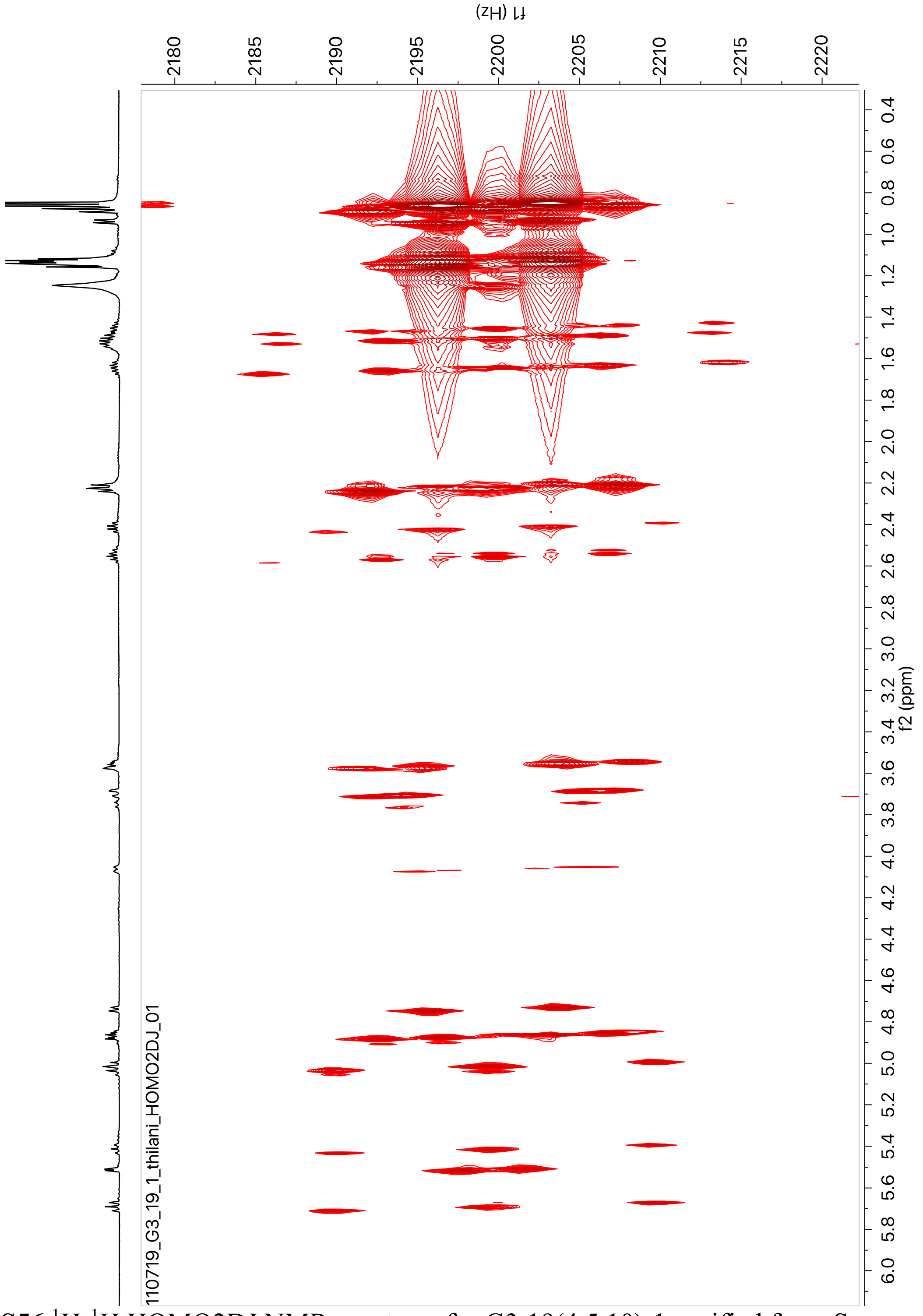

Figure S56 ^{1}H-^{1}H HOMO2DJ NMR spectrum for G3:19(4,5,10)-1 purified from *S. pennellii* LA0716.

Table S15 NMR chemical shifts for G3:19(4,5,10)-2 purified from *S. pennellii* LA0716.

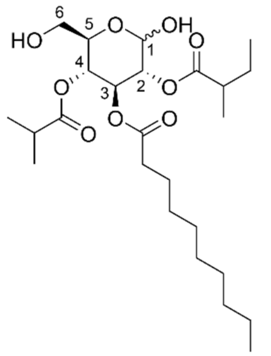

G3:19(4,5,10)-2

Purified from *S. pennellii* LA0716

Chemical Formula: $C_{24}H_{42}O_9$

HRMS: (ESI) *m/z* calculated for $C_{24}H_{42}O_9$ ($[M+NH_4]^+$): 506.3324

Experimental *m/z*: 506.3328

InChI Key: FUSUEACFDRZDFT-VSLCRTCVSA-N
InChI Key (a): FUSUEACFDRZDFT-ONCJQFAMSA-N
InChI Key (b): FUSUEACFDRZDFT-MBFSEYTRSA-N

NMR (500 MHz, $CDCl_3$)

Sample mass: 2 mg

Carbon # (group)	^{1}H (ppm)		^{13}C (ppm) (from HSQC and HMBC)	
	α	β	α	β
**1** (CH)	5.51 (d, J = 3.1 Hz)	4.74 (d, J = 7.6 Hz)	90.25	95.82
**2** (CH)	4.87 (m)	4.86 (m)	71.13	73.31
- 1 (CO)	-	-	176.22	176.22
- 2 (CH)	2.41 (sextet, J = 7.0 Hz)	2.41 (sextet, J = 7.0 Hz)	40.86	40.86
- 3 (CH_3)	1.13 (m, 3H)	1.13 (m, 3H)	16.35	16.35
- 4 (CH_2)	1.45, 1.65 (m, 2H)	1.45, 1.65 (m, 2H)	26.53	26.53
- 5 (CH_3)	0.89 (t, J = 7.3 Hz, 3H)	0.89 (t, J = 7.3 Hz, 3H)	11.76	11.76
**3** (CH)	5.68 (t, J = 9.6 Hz)	5.41 (t, J = 9.7 Hz)	68.66	71.01
- 1 (CO)	-	-	172.62	172.62
- 2 (CH_2)	2.13 (t, J = 7.4 Hz)	2.21 (t, J = 7.4 Hz)	34.08	34.08
- 3 (CH_2)	1.53(m)	1.53(m)	25.13	25.13
- 4,5,6,7 (CH_2)	1.25(m)	1.25(m)	29.33	29.33
- 8 (CH_2)	1.24 (m)	1.24 (m)	32.21	32.21
- 9 (CH_2)	1.25 (m)	1.25 (m)	22.56	22.56
- 10 (CH_3)	0.88 (t, J = 7.0 Hz)	0.88 (t, J = 7.0 Hz)	14.07	14.07
**4** (CH)	5.02 (t, J = 9.7 Hz)	5.02 (t, J = 9.7 Hz)	68.53	68.53
- 1 (CO)	-	-	176.83	176.83
- 2 (CH)	2.54 (hept, J = 7.0 Hz)	2.54 (hept, J = 7.0 Hz)	33.60	33.60
- 3,4 (CH_3)	1.14 (m)	1.14 (m)	18.82	18.82
**5** (CH)	4.04 (ddd, J = 10.2, 4.0, 2.2 Hz)	3.56 (m)	69.49	74.55
**6** (CH_2)	3.57, 3.68 (m)	3.57, 3.68 (m)	60.95	60.95

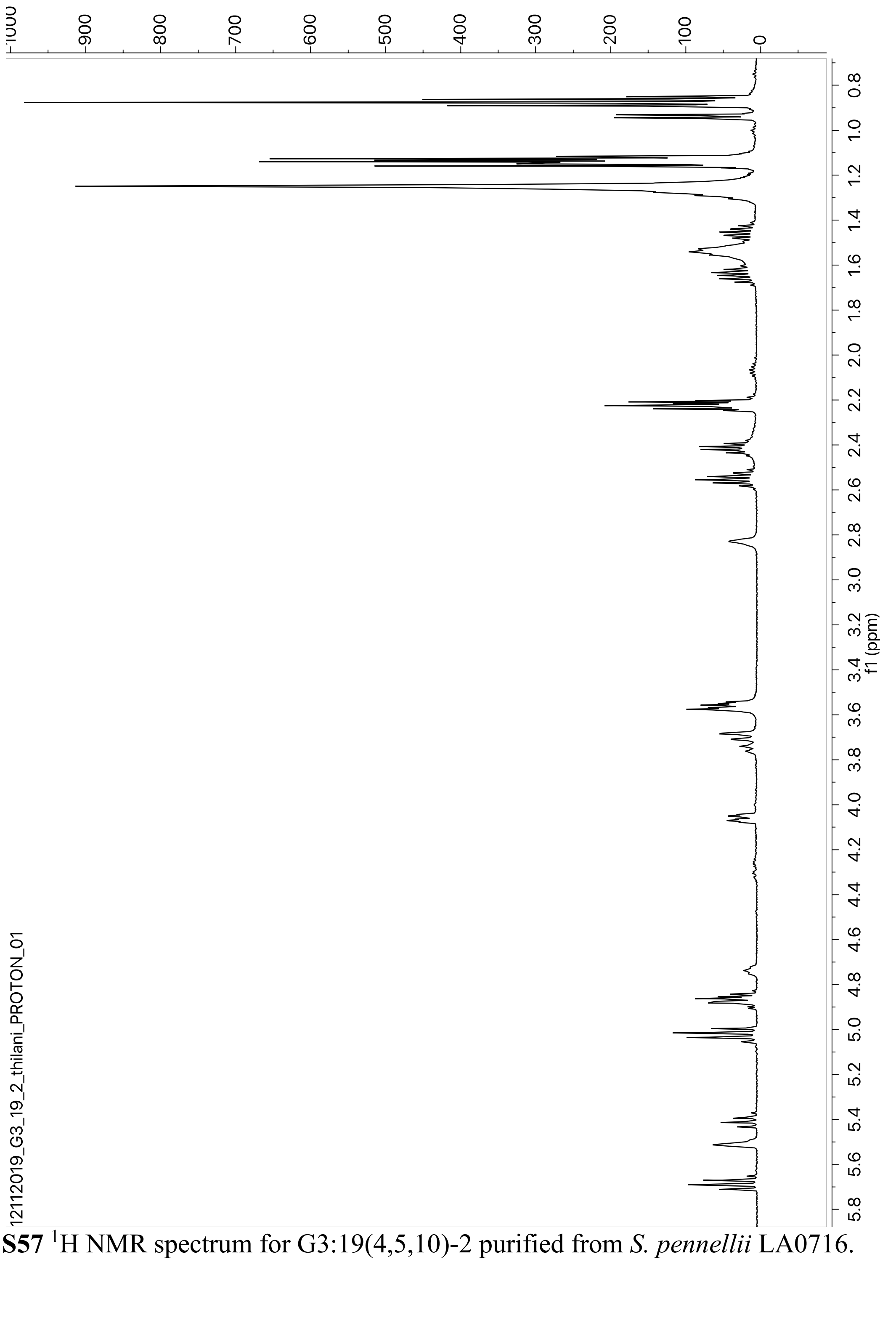

Figure S57 ^{1}H NMR spectrum for G3:19(4,5,10)-2 purified from *S. pennellii* LA0716.

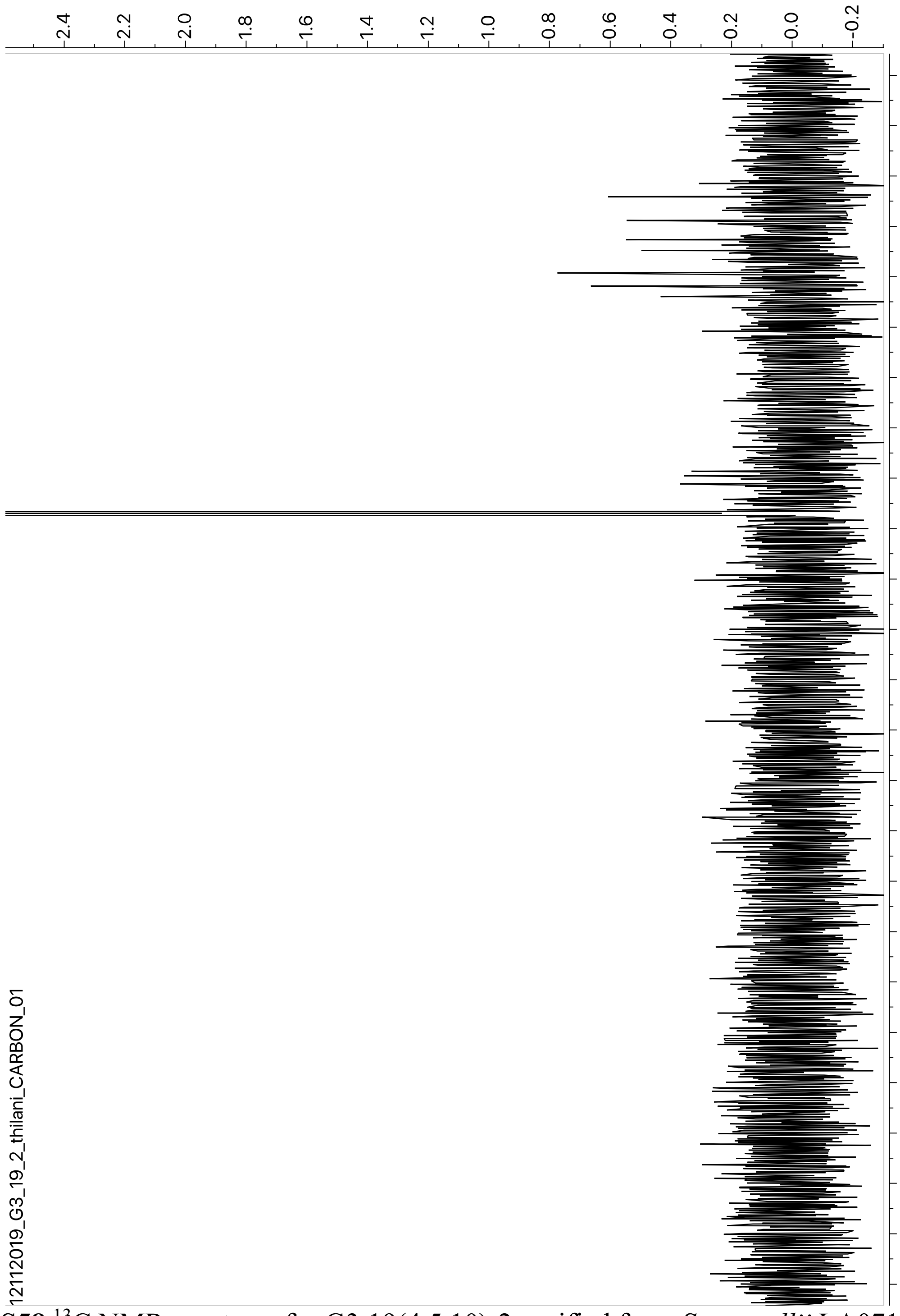

Figure S58 ^{13}C NMR spectrum for G3:19(4,5,10)-2 purified from *S. pennellii* LA0716.

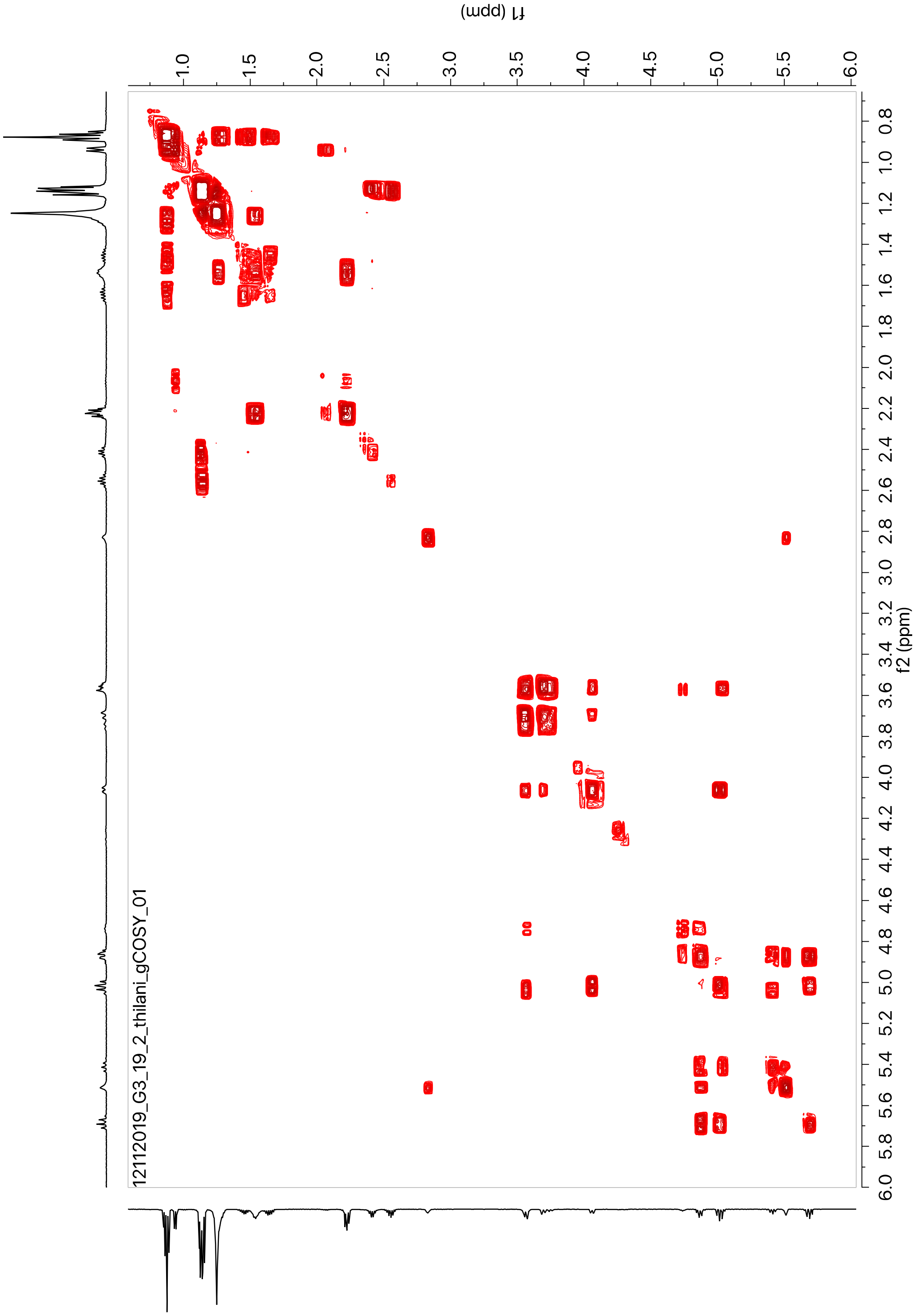

Figure S59 gCOSY NMR spectrum for G3:19(4,5,10)-2 purified from *S. pennellii* LA0716.

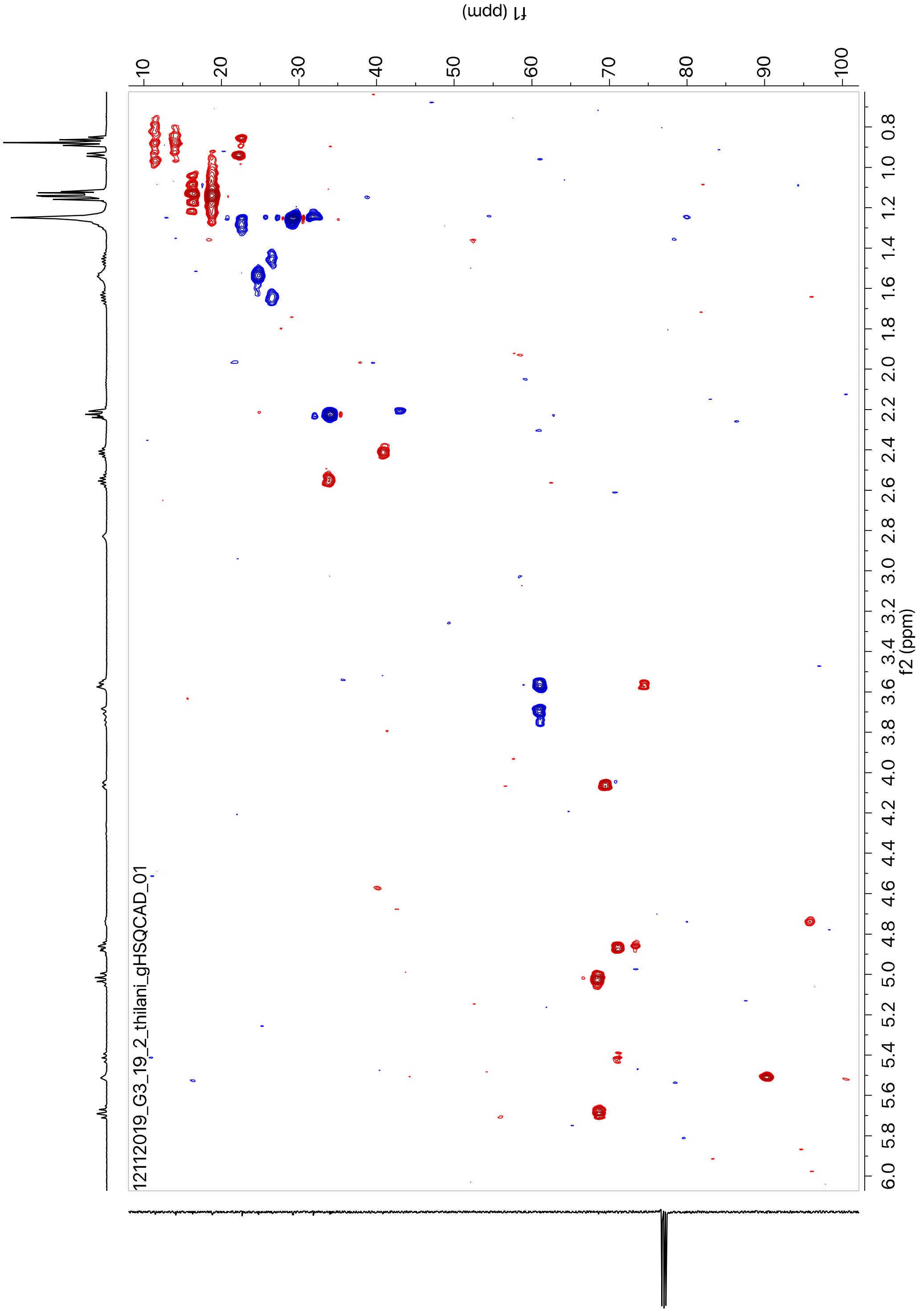

Figure S60 gHSQCAD NMR spectrum for G3:19(4,5,10)-2 purified from *S. pennellii* LA0716.

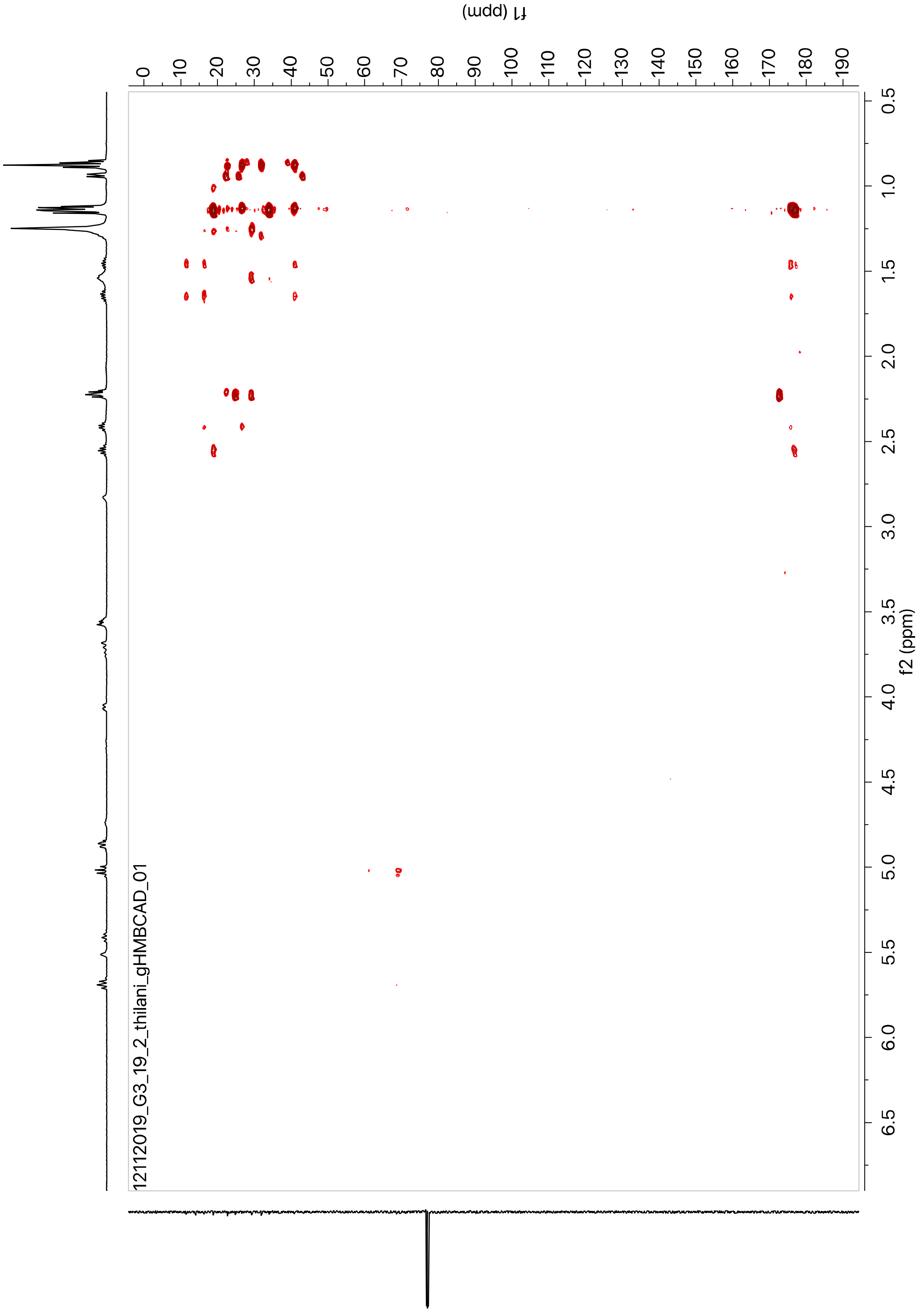

Figure S61 gHMBCAD NMR spectrum for G3:19(4,5,10)-2 purified from *S. pennellii* LA0716.

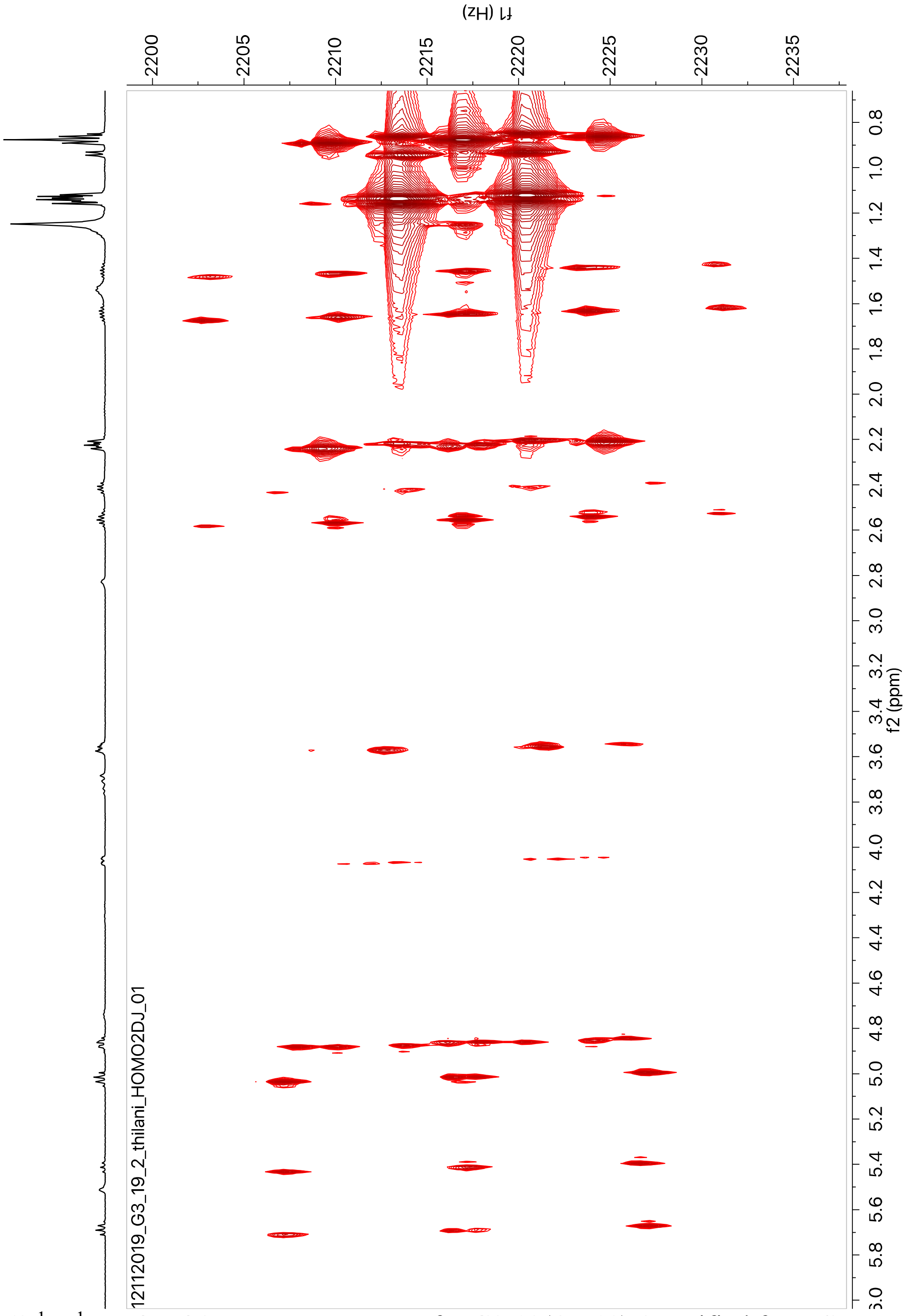

Figure S62 ^{1}H-^{1}H HOMO2DJ NMR spectrum for G3:19(4,5,10)-2 purified from *S. pennellii* LA0716.

Figure S63 S-plot of metabolite features resulting from the OPLS-DA model of north and south region accessions.

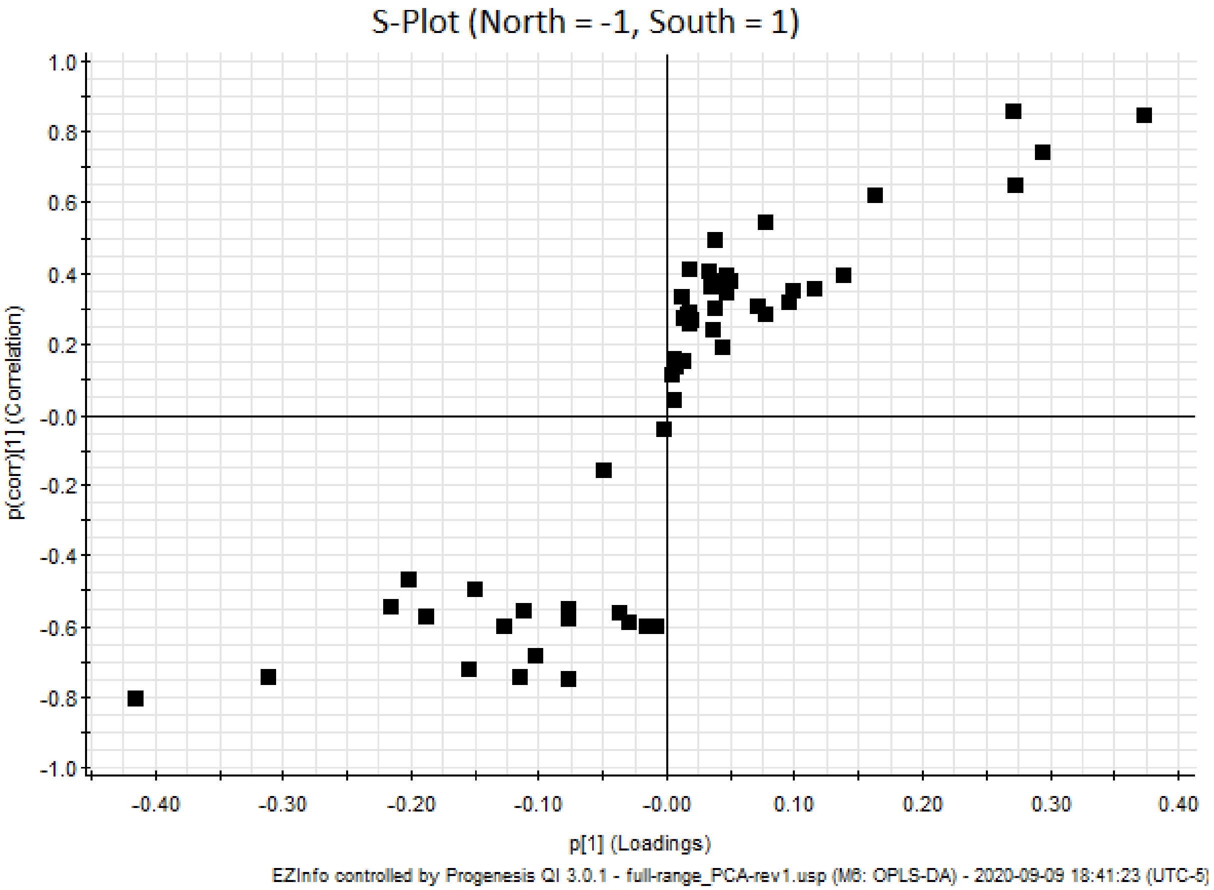

Table S16 Loadings and correlation values for 54 metabolite features from the North range/South range OPLS-DA model.

Compound	North-South load	North-South corr
**G3:15(5,5,5)b**	-4.20E-01	-8.09E-01
**G3:21(5,5,11)a**	-7.67E-02	-7.55E-01
**G3:15(5,5,5)a**	-3.15E-01	-7.51E-01
**G3:21(5,5,11)b**	-1.15E-01	-7.46E-01
**S3:21(5,5,11)**	-1.55E-01	-7.33E-01
**G3:16(5,5,6)a**	-1.04E-01	-6.89E-01
**S3:16(5,5,6)**	-7.95E-03	-6.09E-01
**S3:20(5,5,10)**	-1.89E-01	-6.05E-01
**G3:23(5,6,12)a**	-2.87E-02	-6.04E-01
**S3:15(5,5,5)**	-1.51E-02	-6.01E-01
**G3:23(5,6,12)b**	-3.79E-02	-5.90E-01
**G3:16(5,5,6)b**	-1.24E-01	-5.85E-01
**G3:22(5,5,12)a**	-7.71E-02	-5.80E-01
**G3:22(5,5,12)b**	-1.12E-01	-5.53E-01
**S3:23(5,6,12)**	-7.65E-02	-5.53E-01
**S3:22(5,5,12)**	-2.16E-01	-5.51E-01
**G3:20(5,5,10)a**	-1.52E-01	-5.11E-01
**G3:20(5,5,10)b**	-2.02E-01	-4.73E-01
**G3:14(4,5,5)**	-4.29E-02	-1.34E-01
**flavonoid C**	-2.53E-03	-7.77E-02
**flavonoid A**	2.27E-03	4.50E-02
**S3:21(4,5,12)**	1.23E-02	8.82E-02
**G3:19(4,5,10)-1a**	2.44E-02	9.94E-02
**S3:14(4,5,5)**	7.20E-03	2.26E-01
**S3:12(4,4,4)**	9.82E-03	2.45E-01
**S3:13(4,4,5)**	1.81E-02	2.59E-01
**G4:14(2,4,4,4)b**	1.77E-02	2.70E-01
**G4:15(2,4,4,5)**	1.32E-02	2.95E-01
**G3:17(4,5,8)-1a**	4.68E-02	3.01E-01
**S3:19(4,5,10)-1**	8.45E-02	3.13E-01
**G3:18(4,4,10)-1a**	1.15E-01	3.22E-01
**S3:20(4,4,12)**	4.13E-02	3.23E-01
**G4:14(2,4,4,4)a**	1.87E-02	3.34E-01
**G3:21(4,5,12)b**	4.16E-02	3.42E-01
**S3:18:(4,4,10)-1**	1.06E-01	3.50E-01
**G3:21(4,5,12)a**	2.99E-02	3.59E-01
**S3:17(4,5,8)**	2.33E-02	3.62E-01

Table S16 (cont'd)

Compound	North-South load	North-South corr
**G3:16(4,4,8)-1a**	8.70E-02	3.65E-01
**flavonoid B**	3.41E-02	3.74E-01
**S3:16(4,4,8)**	2.02E-02	3.77E-01
**flavonoid D**	1.70E-02	3.78E-01
**S3:19(4,5,10)-2**	4.70E-02	3.90E-01
**G3:17(4,5,8)-2b**	5.52E-02	4.03E-01
**G3:17(4,5,8)-1b/2a**	1.18E-01	4.07E-01
**S3:17(4,4,9)**	1.44E-02	4.13E-01
**G3:16(4,4,8)-1b/2a**	1.38E-01	4.16E-01
**G3:16(4,4,8)-2b**	5.74E-02	4.25E-01
**G3:20(4,4,12)**	6.97E-02	4.83E-01
**S3:18(4,4,10)-2**	3.86E-02	5.04E-01
**G3:18(4,4,10)-2b**	1.47E-01	5.52E-01
**G3:19(4,5,10)-1b/2a**	2.43E-01	5.70E-01
**G3:18(4,4,10)-1b/2a**	2.66E-01	6.63E-01
**G3:13(4,4,5)**	3.87E-01	8.66E-01
**G3:12(4,4,4)**	2.81E-01	8.75E-01

Figure S64 S-plot of metabolite features resulting from the OPLS-DA model of Pisco and Atico region accessions.

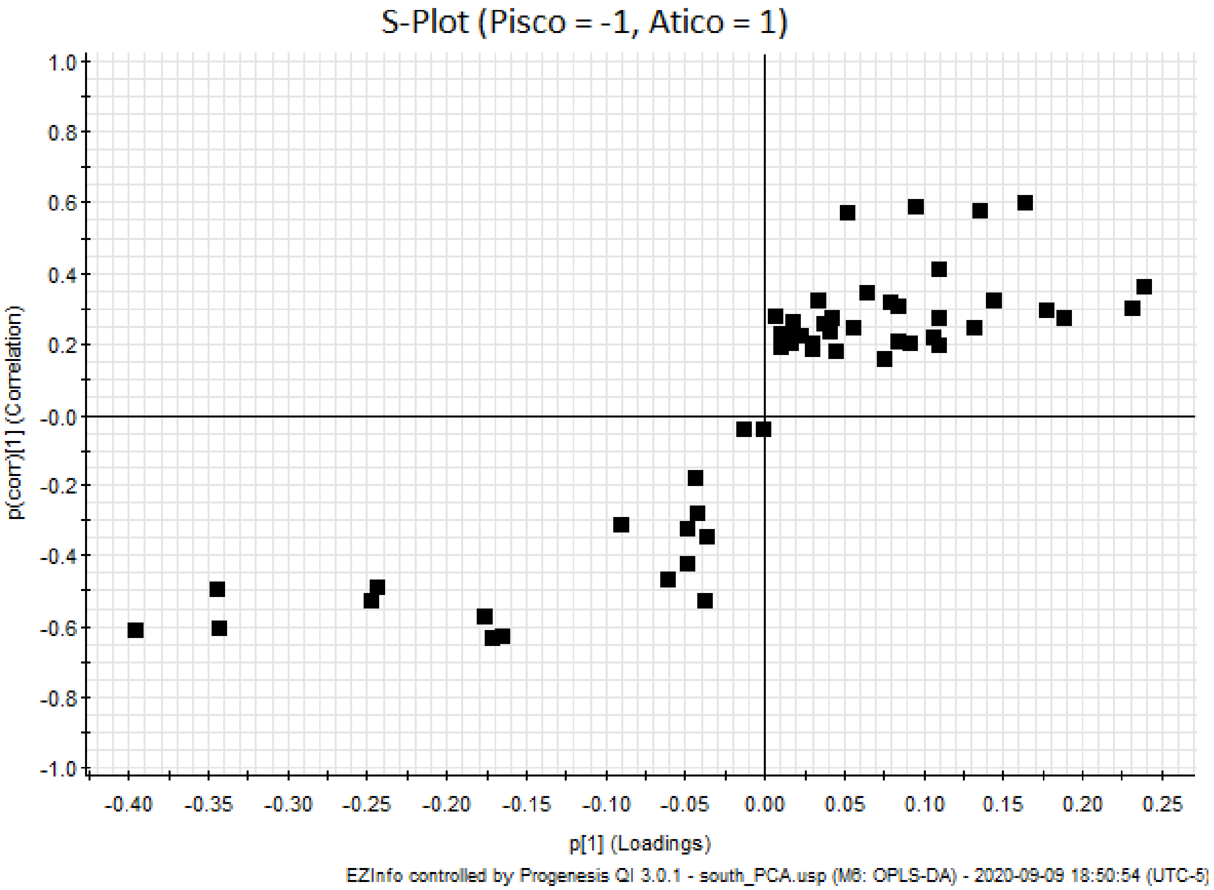

Table S17 Loadings and correlation values for 54 metabolite features from the Pisco region/Atico region OPLS-DA model.

Compound	Pisco-Atico load	Pisco-Atico corr
**G3:16(4,4,8)-1b/2a**	-4.00E-01	-7.11E-01
**G3:17(4,5,8)-2b**	-1.61E-01	-6.99E-01
**G3:17(4,5,8)-1b/2a**	-3.41E-01	-6.95E-01
**G3:16(4,4,8)-2b**	-1.56E-01	-6.86E-01
**G3:17(4,5,8)-1a**	-1.78E-01	-6.63E-01
**G3:16(4,4,8)-1a**	-2.59E-01	-6.35E-01
**S3:17(4,4,9)**	-3.41E-02	-5.81E-01
**S3:17(4,5,8)**	-6.27E-02	-5.70E-01
**G3:12(4,4,4)**	-2.43E-01	-5.68E-01
**S3:16(4,4,8)**	-5.15E-02	-5.62E-01
**G3:13(4,4,5)**	-3.16E-01	-5.25E-01
**G3:16(5,5,6)b**	-9.77E-02	-4.50E-01
**G4:15(2,4,4,5)**	-2.85E-02	-3.75E-01
**G4:14(2,4,4,4)a**	-3.73E-02	-3.50E-01
**G4:14(2,4,4,4)b**	-3.76E-02	-3.44E-01
**G3:14(4,5,5)**	-3.52E-02	-1.45E-01
**S3:18(4,4,10)-2**	-1.43E-02	-1.12E-01
**S3:16(5,5,6)**	-7.72E-04	-4.09E-02
**G3:16(5,5,6)a**	2.47E-02	1.87E-03
**G3:15(5,5,5)b**	8.78E-02	4.38E-03
**S3:19(4,5,10)-2**	7.59E-03	4.34E-02
**flavonoid C**	3.39E-03	5.54E-02
**flavonoid D**	7.23E-03	7.15E-02
**G3:15(5,5,5)a**	8.36E-02	1.01E-01
**S3:23(5,6,12)**	2.67E-02	1.14E-01
**flavonoid B**	3.11E-02	1.67E-01
**S3:22(5,5,12)**	8.00E-02	1.68E-01
**S3:21(5,5,11)**	3.64E-02	1.77E-01
**G3:18(4,4,10)-2b**	9.00E-02	1.98E-01
**G3:23(5,6,12)b**	1.40E-02	2.15E-01
**G3:23(5,6,12)a**	1.06E-02	2.27E-01
**G3:18(4,4,10)-1b/2a**	1.65E-01	2.61E-01
**G3:19(4,5,10)-1b/2a**	2.19E-01	3.11E-01
**G3:18(4,4,10)-1a**	1.81E-01	3.15E-01

Table S17 (cont'd)

Compound	Pisco-Atico load	Pisco-Atico corr
**G3:19(4,5,10)-1a**	1.30E-01	3.27E-01
**G3:20(5,5,10)b**	8.16E-02	3.32E-01
**S3:20(5,5,10)**	6.48E-02	3.36E-01
**G3:22(5,5,12)b**	6.05E-02	3.45E-01
**G3:22(5,5,12)a**	3.94E-02	3.46E-01
**S3:12(4,4,4)**	2.64E-02	3.48E-01
**S3:15(5,5,5)**	6.74E-03	3.54E-01
**S3:13(4,4,5)**	4.79E-02	3.64E-01
**S3:19(4,5,10)-1**	1.70E-01	3.71E-01
**G3:21(5,5,11)b**	3.96E-02	3.73E-01
**G3:21(5,5,11)a**	3.01E-02	3.77E-01
**S3:18:(4,4,10)-1**	2.00E-01	3.88E-01
**S3:14(4,5,5)**	2.16E-02	3.91E-01
**G3:20(5,5,10)a**	8.39E-02	3.93E-01
**S3:20(4,4,12)**	8.91E-02	4.04E-01
**S3:21(4,5,12)**	1.01E-01	4.22E-01
**G3:20(4,4,12)**	1.13E-01	4.38E-01
**flavonoid A**	3.69E-02	4.48E-01
**G3:21(4,5,12)b**	1.01E-01	4.72E-01
**G3:21(4,5,12)a**	7.07E-02	4.78E-01

Figure S65 S-plot of metabolite features resulting from the OPLS-DA model of LA2963 and and the other Atico group accessions. Class 1 = main Atico group accessions; Class 2 = LA2963.

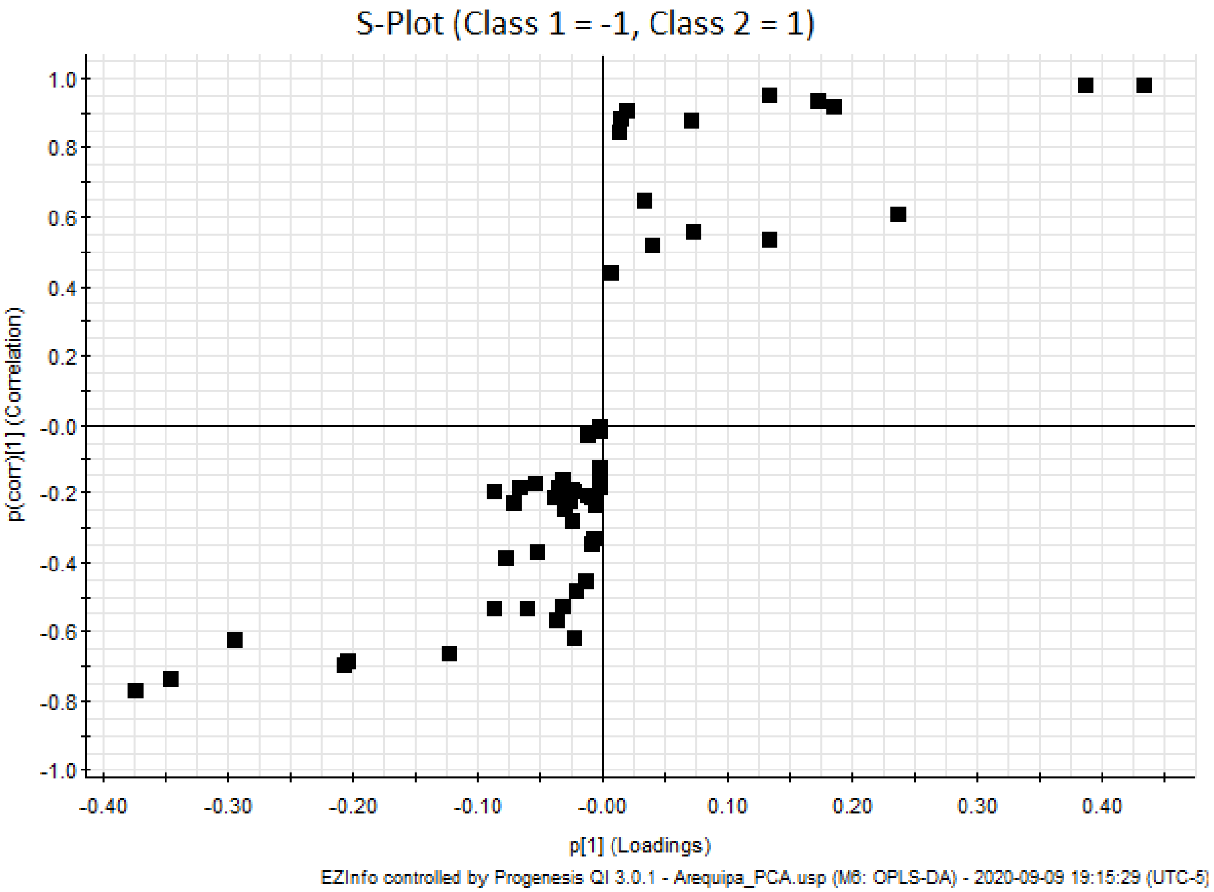

Table S18 Loadings and correlation values for 54 metabolite features from the intraregion Atico OPLS-DA model.

Compound	Atico-LA2963 load	Atico-LA2963 corr
G3:19(4,5,10)-1b/2a	-3.78E-01	-8.04E-01
G3:18(4,4,10)-2b	-2.17E-01	-7.63E-01
G3:20(4,4,12)	-1.30E-01	-7.49E-01
G3:18(4,4,10)-1b/2a	-3.38E-01	-7.39E-01
flavonoid A	-4.11E-02	-6.69E-01
flavonoid C	-2.22E-02	-6.52E-01
G3:19(4,5,10)-1a	-1.91E-01	-6.52E-01
G3:21(4,5,12)a	-6.74E-02	-6.36E-01
G3:21(4,5,12)b	-9.85E-02	-6.35E-01
G3:17(4,5,8)-2b	-2.37E-02	-5.89E-01
G3:18(4,4,10)-1a	-2.69E-01	-5.86E-01
G3:17(4,5,8)-1b/2a	-1.52E-02	-5.65E-01
flavonoid D	-2.98E-02	-5.21E-01
G3:20(5,5,10)a	-8.81E-02	-4.99E-01
flavonoid B	-5.79E-02	-4.76E-01
G3:16(4,4,8)-2b	-7.17E-03	-4.70E-01
G3:20(5,5,10)b	-8.84E-02	-4.14E-01
G3:16(4,4,8)-1b/2a	-7.23E-03	-3.92E-01
G3:21(5,5,11)a	-2.42E-02	-3.55E-01
G3:21(5,5,11)b	-3.11E-02	-3.48E-01
G3:22(5,5,12)a	-2.61E-02	-3.34E-01
G3:22(5,5,12)b	-3.84E-02	-3.21E-01
G3:16(4,4,8)-1a	-4.87E-03	-2.84E-01
G4:14(2,4,4,4)a	-1.83E-03	-2.78E-01
G3:23(5,6,12)a	-7.70E-03	-2.73E-01
G3:23(5,6,12)b	-1.01E-02	-2.66E-01
G4:14(2,4,4,4)b	-4.50E-03	-2.27E-01
S3:16(5,5,6)	-1.90E-03	-2.22E-01
S3:23(5,6,12)	-1.79E-02	-2.20E-01
G3:16(5,5,6)a	-1.96E-02	-2.15E-01
G3:15(5,5,5)b	-7.07E-02	-2.06E-01
G3:15(5,5,5)a	-5.50E-02	-1.88E-01
G3:14(4,5,5)	-2.56E-02	-1.43E-01
G4:15(2,4,4,5)	-5.00E-04	-9.65E-02

Table S18 (cont'd)

Compound	Atico-LA2963 load	Atico-LA2963 corr
**G3:16(5,5,6)b**	-2.49E-02	7.43E-02
**S3:21(5,5,11)**	-2.10E-02	1.84E-01
**S3:22(5,5,12)**	-3.17E-02	1.99E-01
**G3:17(4,5,8)-1a**	3.77E-03	2.85E-01
**G3:12(4,4,4)**	1.25E-01	5.20E-01
**G3:13(4,4,5)**	1.99E-01	5.22E-01
**S3:20(5,5,10)**	4.53E-02	5.57E-01
**S3:15(5,5,5)**	9.00E-03	6.36E-01
**S3:12(4,4,4)**	4.52E-02	7.82E-01
**S3:13(4,4,5)**	8.35E-02	8.27E-01
**S3:16(4,4,8)**	1.42E-02	8.70E-01
**S3:18(4,4,10)-2**	7.11E-02	8.78E-01
**S3:14(4,5,5)**	3.83E-02	8.82E-01
**S3:17(4,5,8)**	1.61E-02	9.10E-01
**S3:17(4,4,9)**	2.07E-02	9.36E-01
**S3:20(4,4,12)**	1.73E-01	9.41E-01
**S3:21(4,5,12)**	1.91E-01	9.55E-01
**S3:19(4,5,10)-2**	1.35E-01	9.59E-01
**S3:19(4,5,10)-1**	3.85E-01	9.92E-01
**S3:18:(4,4,10)-1**	4.30E-01	9.97E-01
